# HLA class I-specific nucleolin peptides induce therapeutic T cells in triple-negative breast cancer patients

**DOI:** 10.7150/ijbs.132389

**Published:** 2026-07-20

**Authors:** Suyanee Thongchot, Niphat Jirapongwattana, Thaweesak Chieochansin, Jaturawitt Prasopsiri, Wannasiri Chiraphapphaiboon, Piriya Luangwattananun, Thanyada Rungrotmongkol, Kamonpan Sanachai, Nitchakan Darai, Doonyapat Sa-nguanraksa, Pornchai O-Charoenrat, Peti Thuwajit, Pa-thai Yenchitsomanus, Chanitra Thuwajit

**Affiliations:** 1 Department of Immunology, Faculty of Medicine Siriraj Hospital, Mahidol University, Bangkok 10700 Thailand.; 2 Siriraj Center of Research Excellence for Cancer Immunotherapy, Research Department, Faculty of Medicine Siriraj Hospital, Mahidol University, Bangkok 10700 Thailand.; 3 Division of Molecular Medicine, Research Department, Faculty of Medicine Siriraj Hospital, Mahidol University, Bangkok 10700 Thailand.; 4 Hematología computacional y genómica (GrHeCo-Xen), Instituto de Investigación Sanitaria de Santiago de Compostela (IDIS), Santiago de Compostela 15706 Spain.; 5 Center of Excellence in Biocatalyst and Sustainable Biotechnology, Department of Biochemistry, Faculty of Science, Chulalongkorn University, Bangkok 10330 Thailand.; 6 Program in Bioinformatics and Computational Biology, College of Interdisciplinary and Integrative Studies, Chulalongkorn University, Bangkok 10330 Thailand.; 7 Department of Biochemistry, Faculty of Science, Khon Kaen University, Khon Kaen 40002 Thailand.; 8 Futuristic Science Research Center, School of Science, Walailak University, Nakhon Si Thammarat 80160 Thailand.; 9 Research Center for Theoretical Simulation and Applied Research in Bioscience and Sensing, Walailak University, Thasala, Nakhon Si Thammarat 80160 Thailand.; 10 Division of Head-Neck and Breast Surgery, Department of Surgery, Faculty of Medicine Siriraj Hospital, Mahidol University, Bangkok 10700 Thailand.; 11 Breast Center, MedPark Hospital, Bangkok 10110 Thailand.

**Keywords:** nucleolin, short peptide, HLA class I, triple-negative breast cancer, immunotherapy

## Abstract

Triple-negative breast cancer (TNBC) is an aggressive subtype with poor prognosis and limited treatment options owing to the lack of expression of common therapeutic targets such as hormone receptors and HER2. Recent advances in immunopeptidomics have enabled the identification of tumor-associated antigen peptides that can elicit tumor-specific immune responses. We identified four novel HLA class I-restricted peptides derived from nucleolin (NCL), a protein overexpressed in TNBC that is associated with poor outcomes. We predicted and validated pNCL-01 through pNCL-04 because of its strong binding affinity to common HLA alleles (A*02:01 and B*15:01), with pNCL-04 demonstrating broad HLA-binding potential across 20 HLA alleles. Peripheral blood mononuclear cells (PBMCs) from both healthy donors and patients with TNBC were pulsed with these peptides, generating NCL-specific CD8^+^ T cells that effectively targeted and killed NCL^+^/PD-L1^+^ TNBC cell lines *in vitro* (MDA-MB-231 and HCC70). These T cells exhibited robust IFN-γ secretion and expressed effector memory markers (CD107a), particularly in cancer tissues of patients with high NCL expression. Notably, pNCL-04 functioned as a shared immunogenic peptide, activating T cells in the majority of TNBC patients (7/10) which was predicted to bind up to 20 different HLA class I alleles, supporting its use as a broadly applicable immunogenic peptide. Furthermore, the combination of NCL-specific T cells with atezolizumab enhanced anti-tumor activity, suggesting the potential benefit of combining NCL-targeted immunotherapy with immune checkpoint blockade. In conclusion, this study demonstrates the potential of HLA-restricted NCL peptides in combination with atezolizumab as a rational peptide-based therapeutic strategy for clinical translation in TNBC patients with high NCL.

## Introduction

Triple-negative breast cancer (TNBC) is one of the deadliest forms of breast cancer and is characterized by poor relapse-free survival rates and prognosis. 1 The absence of targeted treatment options has resulted in survival challenges for patients with TNBC. 2 Immunotherapy has begun to have an impact on patients with advanced TNBC where conventional treatments are ineffective. Recently, immune checkpoint inhibitors (ICIs) have been gained approve for use in these patients. 3, 4 In addition, the generation of tumor antigen-specific T cells using peptide-pulsed peripheral blood mononuclear cells (PBMCs) has been successfully demonstrated in TNBC patients targeting human epidermal growth factor receptors 1 and 2 (HER-1, HER-2), and insulin-like growth factor receptor 1 (IGF-1R) peptides, resulting in a significant tumor response and patient survival extension. 5

Significant peptide-specific CD8^+^ cytotoxic T cells (CTL) or CD4^+^ helper T cell responses have been observed in tumor cells with minimal toxicity and side effects. 6-8 Priming PBMCs with tumor-associated antigen (TAA) peptides enables antigen-presenting cells (APCs) such as myeloid dendritic cells, plasmacytoid dendritic cells, B cells, within the PBMC population to process and present these peptides to T cells 9, thereby initiating antigen-specific immune responses and has proven to be a successful approach for eliminating various tumor variants, including in breast cancer models. 10-15 Furthermore, Taxol-resistance-associated gene-3 (TRAG-3) peptide-pulsed PBMCs demonstrated the capability to induce specific CTLs that significantly lysed MCF-7 cells. 16 Additionally, HER-2/neuprotooncogene (neu) peptide-pulsed PBMCs induced long-lasting HER-2-specific interferon-gamma (IFN-γ) producing CD8^+^ T cells that were capable of lysing HER-2 positive breast cancer cells 17, 18 and HER-1/IGF-1R-expressing TNBC cells. 5

Recent studies have identified nucleolin (NCL) as a potential TAA for TNBC. 19, 20 High NCL expression has been linked to poor outcomes in TNBC patients, 20 and the use of NCL as a tumor antigen presented by dendritic cells has been shown to successfully generate NCL-specific T cells capable of targeting and killing TNBC cells. 20 However, no T cell epitopes of NCL have been reported to date. Our study aimed to identify NCL epitopes restricted to human leukocyte antigens (HLAs), including HLA-A*02:01, which is commonly found worldwide, 21 HLA-A*02:17, HLA-B*15:01, which has been reported in Asian populations, 22, 23 and HLA-B*15:16. 23 We utilized five *in silico* computational platforms: NetMHC, **24** NetMHCpan, **24** NetMHCcons, **25** NetCTLpan, **26** and PickPocket **27** for NCL epitopes with multiple MHC binding predictions, and ensured the accuracy of prediction through molecular dynamics (MD) simulations. We investigated PBMCs from six healthy donors and 10 TNBC patients carrying different levels of NCL in cancer tissues, to determine whether the four candidate NCL peptides (pNCL-01-04) restricted to individual HLA could induce NCL-specific T cells. The obtained NCL-specific T cells successfully induced specific high programmed death-ligand 1 (PD-L1)/NCL-positive MDA-MB-231 (or HCC70) TNBC cell death, especially in combination with the anti-PD-L1 antibody atezolizumab (ATZ), compared to control normal mammary epithelial cell (MCF-10A). Interestingly, T cells from high NCL TNBC patients had high IFN-γ after activation by the NCL peptides. Importantly, a novel single NCL peptide (pNCL-04 demonstrated the ability to bind to 20 different HLA class I alleles, which are the most frequent alleles in the Asian population. This study not only supports the use of the NCL peptide vaccine but also highlights the potential of NCL peptide-pulsed T cell therapy in NCL-positive TNBC patients.

## Materials and Methods

### Cell lines

Human breast cancer cell lines and non-malignant breast epithelial cells were acquired from the American Type Culture Collection, ATCC (Manassas, VA). The MDA-MB-231 line (#HTB-26, ATCC, expressing HLA-A*02:01,-A*02:17, -B*40:02, -B*41:01, -C*02:02, and -C*17:01) was maintained in Dulbecco's Modified Eagle Medium (DMEM) (Thermo Fisher Scientific Inc., CA) supplemented with 10% fetal bovine serum (v/v; Thermo Fisher Scientific Inc.), 100 U/ml penicillin, and 100 µg/ml streptomycin (Sigma-Aldrich, St. Louis, MO). The HCC70 line (#CRL-2315, ATCC, carrying HLA-A*03:01, -A*30:02, -B*15:01, -B*15:16, -C*16:01, and -C*16:01; 28 https://web.expasy.org/cellosaurus/CVCL_1270) was cultured in Roswell Park Memorial Institute 1640 medium (RPMI-1640; Thermo Fisher Scientific Inc.). MCF-10A cells (#CRL-10317, ATCC, carrying HLA-A*01:01, -A*33:01, -B*40:01, -B*55:01, -C*03:03, and -C*07:02) were grown in DMEM supplemented with 5% horse serum (Invitrogen Corporation, Carlsbad, CA), 100 U/ml of penicillin, 100 µg/ml of streptomycin, 20 ng/ml epithelial growth factor (PeproTech, Cranbury, NJ), 0.5 mg/ml hydrocortisone (Sigma-Aldrich), 100 ng/ml cholera toxin (Sigma-Aldrich) and 10 μg/ml insulin (Sigma-Aldrich). All cells were incubated at 37°C in a 5% CO_2_ environment. Cell viability was measured at each passage using a standard trypsinization protocol and then passaged a maximum of 20 times before then experiments were performed. All cell lines were routinely screened every three months by the mycoplasma nested polymerase chain reaction primer set specific for mycoplasma detection (BioDesign Co., Ltd, Pathumthani, Thailand), ensuring that all cell lines remained mycoplasma-free.

### HLA typing

The HLA alleles of all healthy donors and TNBC patients were determined using the LABType XR Class I, A, B, and C Locus Typing kit (RSOX1AT, RSOX1BT, and RSOX1CT; Invitrogen Corporation) at the Department of Transfusion Medicine, Faculty of Medicine Siriraj Hospital, Mahidol University. This method provides high-resolution 4-digit HLA allele identification (**Supplementary Table S1**), which previously accepted for sufficient peptide-HLA interaction studies. 29 Additionally, HLA expression of cell lines (MDA-MB-231 and MCF-10A) was evaluated using CytoFLEX flow cytometry (Beckman Coulter, Brea, CA) employing a FITC-labeled goat anti-HLA-ABC W6/32 monoclonal antibody (14-9983-82; eBioscience Inc., San Diego, CA). The published data of HLA in HCC70 have been accessed (https://www.cellosaurus.org/CVCL_1270).

### Epitope peptide prediction and synthesis

NCL-derived 9-mer peptides with high binding affinities for HLA-A*02:01, HLA-A*02:17, HLA-B*15:01, and HLA-B*15:16 were predicted by the established computer programs following a two-step procedure using established computational programs. Initial predictions were made using five computer-based epitope prediction programs hosted on DTU Bioinformatics Services (http://www.cbs.dtu.dk/services/), including NetMHC (v4.0), NetMHCpan (v4.0), NetMHCcons (v1.1), NetCTLpan (v1.1), and PickPocket (v1.1). These tools aimed to identify peptides from the full-length NCL protein that demonstrate binding to the HLA class I molecules of either MDA-MB-231 or HCC70 cells. Second, peptides were shortlisted based on three out of five predefined criteria encompassing both qualitative and quantitative thresholds from the aforementioned tools to reduce false-positive predictions and improve robustness. The criteria were as follows: NetMHC (threshold for strong binder: %Rank ≤ 0.5 or IC50 < 500 nM and threshold for weak binder: %Rank ≤ 2.0 or IC50 < 2000 nM), NetMHCPan (threshold for strong binder: %Rank ≤ 0.5 or IC50 < 500 nM and threshold for weak binder: %Rank ≤ 2.0 or IC50 < 2000 nM), NetMHCcons (threshold for strong binder: %Rank ≤ 0.5 or IC50 < 500 nM and threshold for weak binder: %Rank ≤ 2.0 or IC50 < 2000 nM), NetCTLpan (threshold for epitope identification: %Rank ≤ 1.0) and PickPocket (prediction values: IC50 < 500 nM ).

All peptides, synthesized by GenScript Biotech Pte. Ltd. (Galaxis West Lobby, Singapore), were purified using C18 reverse-phase high-performance liquid chromatography and achieved a purity of greater than 93%. The lyophilized peptide powder was reconstituted in dimethyl sulfoxide (Sigma-Aldrich) to a final concentration of 14 mg/ml and stored at -20 °C until further use.

### Peptide binding affinity assay

HLA-A*02:01, a classic T2 peptide-binding assay was conducted. 30 HLA-A*02:01-positive and transporter associated with antigen processing (TAP)-deficient T2 cell lines (ATCC CRL-1992) were pre-incubated for 6 h at 37°C in RPMI 1640 serum-free medium. Subsequently, these cells were incubated overnight with varying concentrations of pNCL (0, 12.5, 25, and 50 μg/ml) in RPMI 1640 serum-free medium supplemented with human β2-microglobulin (3 μg/ml; Calbiochem, La Jolla, CA) in a humidified 26 °C incubator with 5% CO_2_ to facilitate low temperature-induced major histocompatibility complex folding. 31 After 18 h, the temperature was increased to 37°C for an additional 2 h to promote peptide-induced major histocompatibility complex folding. The cells were then centrifuged at 200 x *g* for 5 min, followed by staining with an APC-labeled goat anti-HLA-A*02 monoclonal antibody (clone BB7.2; eBioscience Inc.) for 30 min at 4°C. The mean fluorescence intensity (MFI) was measured and analyzed using a CytoFlex flow cytometer (Beckman Coulter). This procedure was repeated in at least three separate experiments, each conducted in triplicate. T2 cells without peptide addition served as a negative control, while phorbol myristate acetate (Sigma-Aldrich) and ionomycin (Sigma-Aldrich) were used as positive controls. The fluorescence index (FI) was calculated using the following formula to quantitatively determine the peptide binding affinity to HLA-A*02:01:

FI = 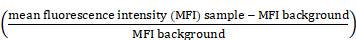


In the calculation formula, “MFI background” denotes the MFI value obtained from the samples without peptides, measured in triplicate, and then MFI was calculated. The FI > 1.5 indicated that the peptide had a high affinity, while 1.0 < FI < 1.5 indicated that the peptide had a moderate affinity, and 0.5 < FI < 1.0 indicated low affinity for the HLA-A*02:01 molecule. 32 In the present study, the reagents used in the T2 peptide-binding assay were employed to evaluate peptide-induced stabilization of HLA-A*02:01 on the cell surface, 33 rather than to assess T cell activation. Therefore, the assay readout reflects peptide-HLA binding affinity based on HLA stabilization, independent of T cell-mediated effects.

### MD for HLA and NCL peptide binding

MD simulations and binding free energy calculations were performed using the AMBER 22 software package [https://ambermd.org/doc12/Amber23.pdf] (The Amber Project, San Francisco, CA). 34-36 HLA models, along with binding 9-mer peptides, were constructed based on crystal structures obtained from the Research Collaboratory for Structural Bioinformatics Protein Data Bank (RCSB PDB; https://www.rcsb.org/). The specific structures used included HLA-A*02:01 (PDB 5C07), HLA-A*02:03 (PDB 3OX8), HLA-A*02:17 (PDB 5C07), HLA-A*24:02 (PDB 5HGH), HLA-B*13:01 (PDB 4JQX); 37 HLA-B*15:01 (PDB 1XR8), HLA-B*15:02 (PDB 6UZM), HLA-B*15:16 (PDB 1XR8), HLA-B*46:01 (PDB 4LCY); 38 and HLA-B*51:01 (PDB 1E27). For establishing a baseline, all HLA class I crystal structure models were treated as positive controls and were simplified to include the α1, α2, and β2 microglobulin domains along with 18 water molecules bound to them.

All MD simulations were carried out under the periodic boundary condition using the isothermal-isobaric ensemble and parameterized with the AMBER ff14SB force field, according to established protocols for HLA studies [10.1007/s00262-024-03627-3; 10.1016/j.heliyon.2024.e36654]. Long-range electrostatics were treated using the particle-mesh Ewald (PME) method, and a 12 Å nonbonded cutoff was applied throughout. Energy minimization was performed in two stages: (*i*) 1,000 steps (500 steepest-descent followed by 500 conjugate-gradient) using a 500 kcal·mol⁻¹·Å⁻² restraint on the complexes, and (*ii*) a 2,500-step unrestrained minimization of the full system. The system was then gradually heated from 10 to 310 K over 100 ps under the NVT ensemble while maintaining a 10 kcal·mol⁻¹·Å⁻² restraint on the solute. Equilibration was conducted under NPT conditions in four stages 50 ps with a 10 kcal·mol⁻¹·Å⁻² restraint, followed by 100 ps, 500 ps, and 200 ps with restraints reduced to 1 kcal·mol⁻¹·Å⁻². Production MD simulations were subsequently performed for 100 ns at 310 K under the NPT ensemble without restraints. All simulations were carried out using the pmemd.cuda.MPI engine on GPU nodes through a SLURM-controlled workflow. 39, 40 The binding free energies (Δ*G*) from the last 20 ns of the simulations were estimated using the solvated interaction energy (SIE) method 39, 40 and the MM/PB(GB)SA approaches. The entropy contributions in the MM/PB(GB)SA calculations were evaluated using normal-mode analysis. 41, 42 Side-chain orientation, anchor-residue distances, and Cα-Cα separation between the P2 and P9 positions were quantified using custom Python scripts and PyRosetta 4.0 (v2020.50).

### Peripheral blood mononuclear cell isolation and peptide stimulation protocol

PBMCs were collected following written informed consent from healthy donors and breast cancer patients, with the sample collection and usage approved by the Siriraj Institutional Review Board (COA no. Si 580/2018). Informed consent was obtained from all healthy donors and following the provision of written informed consent. This study included PBMCs from 6 healthy donors (three each with HLA-A*02 and HLA-B*15) and 10 TNBC patients exhibiting varied NCL levels in clinical tissue samples. PBMCs were isolated using centrifugation over a Ficoll-Paque density gradient and lymphocyte-separating medium (Corning, AZ). Subsequently, 5 x 10^6^ PBMCs per well were seeded in a 12-well culture plate (Corning) and cultured in AIM-V medium (Invitrogen Corporation) supplemented with 5% human AB serum (Thermo Fisher Scientific Inc.), interleukin-2 (IL-2, 20 U/ml; ImmunoTools GmbH, Friesoythe, Germany), and interleukin-7 (IL-7, 10 ng/ml, ImmunoTools GmbH). PBMCs were stimulated with 10 µg/ml of synthetic peptides. Every 3 days, half of the culture medium was replenished with fresh medium containing IL-2 (20 U/ml) and IL-7 (10 ng/ml). PBMCs were restimulated every 7 days with fresh medium containing these peptides. On day 3 following the three weeks of the experiment, the activated T cells were harvested and prepared for functional analysis.

DNA from PBMCs of both healthy donors and patients was extracted using the DNeasy Blood and Tissue Kits (Qiagen Inc., Valencia, CA). Subsequent class I HLA allele typing was conducted using the LABType XR class I, A, B, and C Locus Typing Kit at the Department of Transfusion Medicine, Faculty of Medicine Siriraj Hospital, Mahidol University (RSOX1AT, RSOX1BT, and RSOX1CT; Invitrogen Corporation; **Supplementary Table S1**).

### Surface and intracellular cytokine staining

The following antibodies were used for intracellular cytokine staining: FITC-conjugated anti-CD3 (#21620033), APC-conjugated anti-CD4 (#21270046), APC-conjugated anti-CD8 (#21620086), and PerCP-conjugated anti-CD8 (#21620085) obtained from ImmunoTools GmbH. APC-conjugated anti-IFN-γ (17-7319-82), eFluor450-conjugated anti-CD3 (#48003741), Alexa Fluor700-conjugated anti-CD4 (#56004942), APC-Cy7-conjugated anti-CD8 (A15448), PerCP-conjugated anti-CD69 (MA1-10277), and FITC-conjugated anti-CD107a (11-1079-42) were sourced from Thermo Fisher Scientific Inc. PE-conjugated anti-PD-1 (CD279 #367404) obtained from BioLegend (San Diego, CA). For each assay, 1 × 10^6^ events were recorded using a BD LSRFortessa Cell Analyzer (BD Biosciences, Franklin Lakes, NJ), with isotype antibodies serving as negative controls. Data were analyzed with FlowJo VX software (Tree Star Inc., Ashland, OR), presenting the geometric MFI for each marker, normalized against isotype control values.

Briefly, on the third day after the third round of peptide stimulation, 1 x 10^6^ PBMCs were treated with peptide epitopes (10 µg/ml) for 6 h, followed by a 2 h incubation with Brefeldin A (1 µg/ml; Sigma-Aldrich) and Monensin (1 µg/ml; Sigma-Aldrich) to inhibit further cytokine release. Subsequently, cells were washed with sterile 1x phosphate-buffered saline, and a 30-min incubation with anti-CD3, anti-CD8, anti-CD4, and anti-CD69 antibodies. The cells were fixed using a fixation/permeabilization solution kit (555028; BD GolgiPlug, Franklin Lakes, NJ) and stained with anti-IFN-γ antibody (17-7319-82; Thermo Fisher Scientific Inc.) for 30 min on ice in the dark. Analysis was performed on a CytoFLEX flow cytometer (Beckman Coulter) and BD LSRFortessa Cell Analyzer (BD Biosciences), using FlowJo version 10.3 software (Tree Star) for data processing.

### IFN-γ enzyme-linked immunosorbent spot assay

IFN-γ produced by peptide-specific effector T cells was quantified using an enzyme-linked immunosorbent spot assay (Human IFN-γ ELISpot Basic Kit; Mabtech Inc., Cincinnati, OH), executed per the instructions provided by the manufacturer. Initially, plates were coated with anti-IFN-γ antibody and stored at 4°C overnight. Subsequently, 5 x 10^3^ MDA-MB-231 and HCC70 cells were plated and incubated overnight at 37°C. Then, 1 x 10^5^ PBMCs were added to the cells, followed by further incubation for 24 h at 37°C. Phorbol myristate acetate (P1585; Sigma-Aldrich) and Ionomycin (I9657; Sigma-Aldrich), each at a concentration of 1 μg/mL, served as positive controls across all experiments, while PBMCs sensitized without target cells acted as the negative control. Detection of IFN-γ involved the addition of biotinylated IFN-γ detection antibody and streptavidin-alkaline phosphatase to each well, which was then developed with a color solution using the BCIP/NBT-plus substrate solution (3650-10; Mabtech Inc.). Spot quantification was performed using an ELISpot plate reader (Bioreader 5000 Fγ; Biosys Technologies, Dhaka, Bangladesh), with spot counting facilitated by CellCounter software, version 0.2.1 (https://nghiaho.com/). All experimental groups were assessed in triplicate.

### Western blot analysis

For Western blotting, 60 µg of protein from cultured tumor cells was quantified using the Bradford Kit (Bio-Rad Laboratories Srl, Segrate, Italy), separated by SDS-PAGE, and transferred onto polyvinylidene fluoride membranes (Whatman GmbH, Dassel, Germany) via electroblotting. The membranes were then incubated with primary antibodies, all diluted to 1:500 (rabbit polyclonal anti-NCL antibody #14574 from Cell Signaling Technology Inc., MA; rabbit anti-PD-L1 antibody ab205921 from Abcam Ltd., Cambridge, MA) overnight at 4°C. An anti-β-actin antibody (1:10000; sc-47778; Santa Cruz Biotechnology, CA) served as the loading control. For detection, the membranes were incubated with HRP-conjugated goat anti-rabbit (ab6721; Abcam Ltd.) or HRP-conjugated goat anti-mouse (7076S; Cell Signaling Technology Inc.) secondary antibodies for 1 h at room temperature. The detection of immunoreactive bands was achieved using Enhanced Chemiluminescence Plus solution (Thermo Fisher Scientific Inc.) and visualized on a Gel Document system (Syngene, Cambridge, UK). Band quantification was performed using ImageJ software (version 1.48v; National Institutes of Health, Bethesda, MD).

### Immunofluorescence staining

To evaluate NCL and PD-L1 expression levels in MDA-MB-231, HCC70, and MCF-10A cell lines, 5000 cells/well were cultured on sterile glass coverslips for 24 h. Subsequently, cells were fixed with ice-cold absolute methanol. Nonspecific protein binding was blocked using 3% bovine serum albumin (BSA)/1x PBS, then incubated overnight at 4°C in a humidified chamber with the primary antibodies, rabbit polyclonal anti-NCL antibody (1:50; Cell Signaling Technology Inc.) and rabbit anti-PD-L1 antibody (1:50; Abcam Ltd.). Following primary antibody incubation, cells were washed and incubated with appropriate secondary fluorescent antibodies at room temperature for 3 h. Nuclei were stained with Hoechst33342 (1:1000; Invitrogen Corporation). Confocal imaging was performed using a Zeiss LSM 800 microscope (Carl Zeiss, Jena, Germany) at the Division of Molecular Medicine, Faculty of Medicine Siriraj Hospital, Mahidol University. Equipment details were microscope model: AxioObserver7, objective lens: Plan-Apochromat 63x/1.4NA oil immersion, and laser: Diode 561 nm. The acquisition software was ZEN 2.3 software (Blue Edition, 2002-2011).

### 2-D tumor cell killing assay

The mWasabi or CellTracker Green CMFDA (5-chloromethylfluorescein diacetate) target cells: MDA-MB-231 (HLA-A*02:17; NCL^+^/PD-L1^+^), HCC70 (HLA-B*15:16; NCL^+^/PD-L1^+^), and MCF-10A (NCL^-^/PD-L1^-^) were seeded at 10^4^ cells per well into 96-well plates (Corning). Effector T cells were subsequently co-incubated with the target cancer cells at varying effector-to-target (E:T) ratios (50:1, 25:1, and 1:1) for 24 h in a 37°C incubator supplemented with 5% CO_2_. After incubation, target cell viability was quantitatively assessed using the Pierce Firefly Luciferase Glow Assay Kit (Thermo Fisher Scientific Inc.) and Lumat LB 9507 Ultra-Sensitive Luminometer (Berthold Technologies GmbH & Co. KG., Bad Wildbad, Germany). The extent of cancer cell lysis was determined using the following formula:

% Cancer cell lysis = 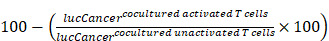


### 3-D tumor spheroid killing assay

To initiate the formation of 3-D tumor spheroids, mWasabi or CellTracker Green CMFDA fluorescence target cells were cultured in Ultra Low Attachment 96-Well Round-Bottom plates (Corning) at a density of 1 x10^4^ cells per well, mixed with 2.5% Matrigel (Corning). To promote spheroid formation, the plates were centrifuged for 3 min at 300 x *g* at 4ºC to initiate the formation of 3-D spheroids. T cells, labeled with CellTracker Orange CMRA Dye (C2927; Thermo Fisher Scientific Inc.), were then added to the cancer cell spheroids at effector-to-target ratios of 50:1, 25:1, and 1:1. Fluorescence signals emanating from interactions between effector T cells and target spheroids were captured using an inverted fluorescence microscope coupled with CellSense Standard software (version 1.15; Olympus UK Ltd., Middlesex, UK). The ImageJ software (National Institutes of Health) was used to quantify the change in MFI relative to the spheroid-inactivated T cells.

Additionally, time-lapse video clips illustrating the cell killing process at an E:T ratio of 50:1 were generated from a series of 20-frame images captured by confocal microscopy. (Zeiss LSM 800; Carl Zeiss; original magnification 63x). The data acquisition and analysis were facilitated by ZEN 2.3 software (Blue Edition, 2002-2011).

### Colony formation assay

MDA-MB-231, HCC70, and MCF-10A cells were seeded at a concentration of 10,000 cells per well into 96-well plates (Corning) and cultured overnight. On the following day, 10 µg/ml of ATZ (A2004; Selleckchem, Houston, TX) and effector T cells were added to the wells at the specified effector-to-target ratios and incubated for 24 h at 37°C. Afterwards, T cells were removed, and the cancer cells were washed with 1x PBS, fixed with absolute methanol for 15-30 min, and subsequently stained with 0.5% w/v crystal violet solution. The number of remaining cancer cell colonies was counted using photometric measurements facilitated by CellCounter software (https://nghiaho.com/). Experiments were performed in three duplicate wells and repeated three times to ensure reliability. Percent cytotoxicity was calculated using the following formula to quantify the cytolytic activity of effector T cells against cancer cell colonies:

% Cytotoxicity = 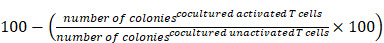


### Statistical analysis

Statistical analyses were conducted utilizing IBM SPSS Statistics (version 20; IBM Corp, Armonk, NY) and GraphPad Prism (version 7.04; GraphPad Software Inc, San Diego, CA). The data are presented as mean ± standard deviation (SD) derived from a minimum of three independent experiments. Student's t-test and two-sided ANOVA followed by Tukey's post-hoc test were performed for comparison of ≥ 2 samples. *P* less than 0.05 was considered a statistically significant difference.

## Results

### *In silico* and MD simulation analysis of NCL peptide candidates

To predict the HLA-A*02:01, HLA-A*02:17, HLA-B*15:01, and HLA-B*15:16-restricted T cell epitopes of NCL, we employed the software NetMHC, NetMHCpan, NetMHCcons, NetCTLpan, and PickPocket. These packages were used to predict the potential peptides with high binding ability. The top-ranking 9-amino acid peptides with the highest scores for each HLA were summarized as 11 peptides for HLA-A*02:01, 13 for HLA-A*02:17, 11 for HLA-B*15:01, and 12 for HLA-B*15:16 (**Table 1**). Finally, four peptides that passed at least 3 of these 5 algorithms were selected, ranking from the highest to lowest binding score as: pNCL-01_15-23_ (KMAPPPKEV) and pNCL-02_488-496_ (VLSNLSYSA) for HLA-A*02:01; and pNCL-03_486-494_ (TLVLSNLSY) and pNCL-04_524-532_ (YAFIEFASF) for HLA-B*15:01 (**Table 1**).

We analyzed the structures and binding affinity of these peptides to either HLA-A*02:17 or HLA-B*15:16, which represent the HLAs of MDA-MB-231 and HCC70, using MD simulation (**Table 1**). As the HLA-A*02:17 and HLA-B*15:16 models were not available in the Research Collaboratory for Structural Bioinformatics Protein Data Bank (RCSB PDB; https://www.rcsb.org/), we instead utilized 5C07 (complex with HLA-A*02:01 carrying YQFGPDFPIA) 43 and 1XR8 (HLA-B*15:01 in complex with UbcH6 and Epstein-Barr Virus EBNA-3). 44 The results demonstrated that the four pNCLs satisfied the criteria of HLA-A*02:01-restricted and HLA-B*15:01-restricted T cell epitopes similar to 5C07 and 1XR8 epitopes, as evidenced by binding free energy scores Δ*G*_MM/GBSA_, Δ*G*_MM/PBSA_, and Δ*G*_SIE_ (**Table 2**).

Specifically, the results showed that the Δ*G*_MM/GBSA_ of complexes between pNCLs and HLA-A*02:01: pNCL-01, pNCL-02, pNCL-03, and pNCL-04 were -10.50 ± 1.20 kcal/mol, -11.62 ± 3.45 kcal/mol, -12.37 ± 5.00 kcal/mol, and -15.52 ± 2.77 kcal/mol, in comparison with -14.61 ± 1.20 kcal/mol for the 5C07 control. For the Δ*G*_MM/GBSA_ of complexes between pNCLs and HLA-B*15:01 were -12.33 ± 6.72 kcal/mol, -22.19 ± 2.40 kcal/mol, -31.36 ± 3.57 kcal/mol, and -16.06 ± 2.91 kcal/mol, in comparison with 1XR8 control (-23.28 ± 4.17 kcal/mol) (**Table 2**).

Regarding the Δ*G*_MM/PBSA_ of complexes between pNCLs and HLA-A*02:01 were -26.02 ± 1.07 kcal/mol for pNCL-01, -29.76 ± 1.33 kcal/mol for pNCL-02, -24.90 ± 3.63 kcal/mol for pNCL-03, and -28.19 ± 1.54 kcal/mol for pNCL-04, compared to -29.25 ± 1.07 kcal/mol for the 5C07 control. For the Δ*G*_MM/PBSA_ with HLA-B*15:01 were -22.25 ± 6.47 kcal/mol, -30.12 ± 1.68 kcal/mol, -31.45 ± 3.12 kcal/mol, and -25.22 ± 1.14 kcal/mol, in comparison with -30.06 ± 2.96 kcal/mol for the 1XR8 control (**Table 2**).

**Figure 1:**
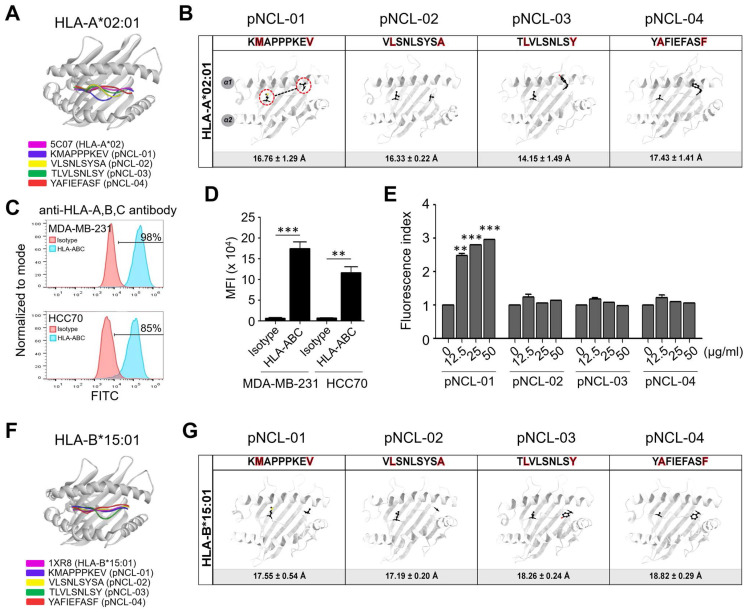
Computational modeling of nucleolin short peptide binding to HLA-A*02:01 and HLA-B*15:01 grooves. (A) Construction of the HLA-A*02:01 model (PDB 5C07, DOI: 10.2210/pdb5C07/pdb), incorporating four pNCL peptides using crystallographic data, is represented in gray. **(B)** The depiction of the peptide within the binding groove, shown as a backbone structure in gray, with the second and ninth amino acid positions highlighted in yellow to indicate anchor residue binding to HLA-A*02:01. The upper panel illustrates the peptide's orientation, while the lower panel affords a side view of peptide-HLA complex formation during molecular dynamics simulation. The distance between P2 and P9 peptide residues is given below. **(C and D)** Flow cytometry analysis of HLA-ABC expression levels in MDA-MB-231 and HCC70 cells. **(E)** T2 cell binding assay for HLA-A*02, visualized using FITC-anti-HLA-A, B, C antibody and quantified by mean fluorescence intensity. **(F-G)** Comparative computational models detailing the overlay of HLA-B*15:01 (PDB 1XR8, DOI: 10.2210/pdb1XR8/pdb) interaction with the four candidate peptides. The pictures indicated the replicates of three independent experiments. Statistical analysis was performed using one-way ANOVA followed by Tukey's multiple comparisons test. Data are expressed as mean ± standard deviation. Statistical significance: **, *P* < 0.01 and ***, *P* < 0.001; determined using a two-tailed unpaired t-test.

Furthermore, a recently developed approach utilizing a similar methodology called Δ*G*_SIE_ 45 showed HLA-A*02:01-restricted pNCLs, including pNCL-01 (-11.59 ± 0.39 kcal/mol), pNCL-02 (-12.61 ± 0.24 kcal/mol), pNCL-03 (-12.02 ± 0.49 kcal/mol), and pNCL-04 (-12.47 ± 1.45 kcal/mol), in comparison with the -12.88 ± 0.04 kcal/mol of the 5C07 control (**Table 2**). Meanwhile, the Δ*G*_SIE_ with HLA-B*15:01 for different pNCLs were -11.41 ± 1.30 kcal/mol for pNCL-01, -14.57 ± 0.59 kcal/mol for pNCL-02, -15.18 ± 0.32 kcal/mol for pNCL-03, and -13.28 ± 0.71 kcal/mol for pNCL-04, in comparison with the -14.60 ± 0.27 kcal/mol of the 1XR8 control. All peptides were confirmed to be accommodated in the center of the binding groove of HLA-A*02:01 (**Fig. 1A and B**) and of HLA-B*15:01 (**Fig. 1F and G**).

The attractive binding affinities of the peptide in the cleft and anchor side chain were demonstrated by the distance between the second (P2) to the ninth (P9) amino acidresidues interacting with the hydrophobic binding pockets, denoted as d[P2-P9] (**Table 2**). For HLA-A*02:01/pNCL-01, HLA-A*02:01/pNCL-02, HLA-A*02:01/pNCL-03, and HLA-A*02:01/pNCL-04, the distances were 16.76 ± 1.29 Å, 16.33 ± 0.22 Å, 14.15 ± 1.49 Å, and 17.43 ± 1.41 Å, respectively, while the positive control, HLA-A*02:01/5C07, exhibited a distance of 18.85 ± 0.30 Å (**Table 2**). Similarly, for HLA-B*15:01/pNCL-01, HLA-B*15:01/pNCL-02, HLA-B*15:01/pNCL-03, and HLA-B*15:01/pNCL-04, the distances were 17.55 ± 0.54 Å, 17.19 ± 0.20 Å, 18.26 ± 0.24 Å, and 18.82 ± 0.29 Å, in comparison to the positive control HLA-B*15:01/1XR8 (21.28 ± 0.75 Å; **Table 2**).

### *In vitro* analysis of NCL peptide binding affinity to the HLA-A*02:01

The T2 cell-peptide binding test assessed the binding affinity of NCL peptide candidates to the HLA-A*02:01. Four NCL peptides were found to bind to HLA-A*02:01 molecules with varying affinities (**Fig. 1E**). The cell surface expression of the HLA-A, HLA-B, and HLA-C receptors was observed at 86.5 ± 11.5% (mean fluorescence intensity [MFI], 18.5 x 10^4^) in MDA-MB-231 and 71.8 ± 13.2% (MFI, 13.4 x 10^4^) in HCC70 cells (**Fig. 1C and D**). Specifically, pNCL-01 demonstrated significantly higher affinity binding to HLA-A*02:01 in a dose-dependent manner, with the fluorescence intensities (FIs) of 2.37 ± 0.93 at 12.5 µg/ml, 3.25 ± 0.68 at 25 µg/ml, and 3.55 ± 0.16 at 50 µg/ml, compared to those of untreated cells (**Fig. 1E**). However, pNCL-02, pNCL-03, and pNCL-04 exhibited low binding affinity to HLA-A*02:01, with FIs of approximately 0.90 ± 0.3 at all concentrations (**Fig. 1E**).

### Measuring CD8^+^ T cell responses after sensitization with 9-amino acid NCL peptides

Flow cytometric analyses revealed that NCL-specific T cells contained a slightly higher proportion of CD3^+^/CD8^+^ T cells compared to the control T cells with no peptide treatment or unpulsed condition (UP) (**Supplementary Fig. S1A-F**). HLA-A*02 HDs no. 1-3 exhibited 47.8 ± 6.6% CD3^+^/CD8^+^ T cells, while HLA-B*15 HDs no. 4-6 had 46.4 ± 6.9% CD3^+^/CD8^+^ T cells. Similarly, CD3^+^/CD4^+^ T cells in HLA-A*02 HDs were 52.2 ± 5.9% and in HLA-B*15 HDs were 53.5 ± 7.8%, in comparison with the control T cells with no peptide treatment (HLA-A*02 HDs: 38.4 ± 1.3% CD3^+^/CD8^+^ T cells and 38.4 ± 3.8% CD3^+^/CD4^+^ T cells; HLA-B*15 HDs: 36.4 ± 6.9% CD3^+^/CD8^+^ T cells and 40.5 ± 6.9% CD3^+^/CD4^+^ T cells; **Supplementary Fig. S1A-F**).

**Figure 2:**
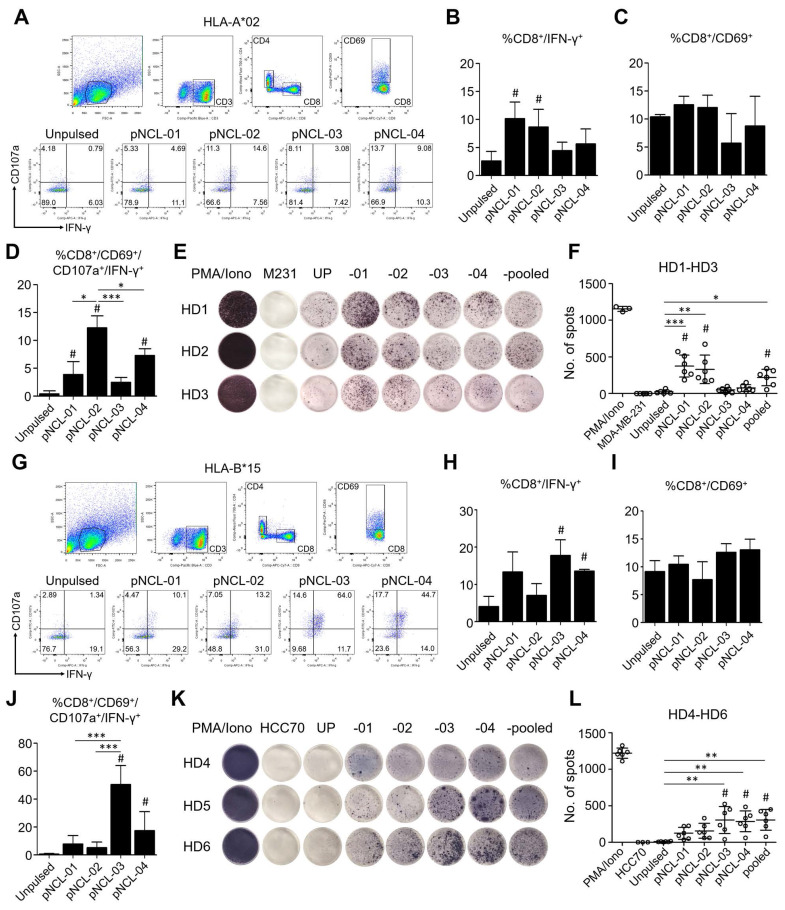
** Analysis of T cell responses from PBMCs of six healthy donors (HDs) via intracellular cytokine staining (ICS) and ELISpot assays. (A-F)** Focus on HLA-A*02. **(G-L)** Focus on HLA-B*15. **(A and G)** Flow cytometric phenotypic characterization of representative PBMC samples employing CD3-eFluor450, CD4-Alexa Fluor700, CD8-APC Cy7, CD69-PerCP, CD107a-FITC, and IFN-γ-APC. Data depicted are mean ± SD from 2-3 independent experiments per donor, with bars indicating mean ± SD. **(E-F and K-L)** ELISpot assays were conducted to evaluate IFN-γ production in response to pNCL peptides identified by PBMCs from healthy donors, HDs 1-3 **(E-F)** and from healthy donors, HDs 4-6 **(K-L)**. PBMCs sensitized with PMA/Ionomycin served as a positive control (PMA/Iono), while PBMCs without peptide exposure (unpulsed, UP) functioned as the negative control. MDA-MB-231 and HCC70 cells treated with NCL peptides were included as additional negative controls. Statistical significance: # denotes comparison to unpulsed; *, *P* < 0.05; **, *P* < 0.01; ***, *P* < 0.001; determined using a one-way ANOVA, followed by Tukey's post-hoc test, was used to calculate statistical differences. HD, healthy donor; pooled, pooled NCL peptides; UP, unpulsed control peptide.

By the end of the experiment study on day 21, significant induction of IFN-γ producing CD8^+^ T cells was observed in HLA-A*02 HDs' PBMCs pulsed with pNCL-01 and pNCL-02 stimulations as 10.5 ± 4.2% and 9.7 ± 3.8% (**Fig. 2A and B**). No significant increment of IFN-γ producing CD8^+^ T cells was observed after activation with pNCL-03 and pNCL-04. However, no increment in CD69^+^/CD8^+^ T cells was detected under any pNCL pulsing conditions (**Fig. 2C**). Moreover, a significant rise in the frequency of CD3^+^/CD8^+^/CD69^+^/CD107a^+^/IFN-γ^+^ T cells from HLA-A*02 HDs was noted following pulsing with pNCL-02 (12.9 ± 2.6%), pNCL-04 (6.1 ± 2.3%), and pNCL-01 (5.0 ± 1.2%), as opposed to the UP condition, which showed a baseline frequency of 3.0 ± 1.1% (**Fig. 2D**).

In HLA-B*15 HD-derived PBMCs, CD8^+^ T cells exhibited significant production of IFN-γ responses following stimulation with pNCL-03 (19.8 ± 3.5%) and pNCL-04 (15.1 ± 0.7%; **Fig. 2G and H**). No increment in CD69^+^/CD8^+^ T cells was noted across all pNCL pulsing scenarios (**Fig. 2I**). Of note, the percentage of CD3^+^/CD8^+^/CD69^+^/CD107a^+^/IFN-γ^+^ T cells from HLA-B*15 HDs was substantially higher in cultures pulsed with pNCL-03 (50.9 ± 12.1%) and pNCL-04 (16.8 ± 15.7%), compared to the unpulsed T cells (0.2 ± 1.2%), with a statistically significant difference (**Fig. 2J**).

PBMCs isolated from HLA-A*02 HDs (**Fig. 2E and F**) and HLA-B*15 HDs (**Fig. 2K and L**) underwent repeated stimulations with all four predicted peptides and pooled peptides. Initial testing at day 0 revealed no significant increase in IFN-γ^+^ spots (**Supplementary Fig. S2**). In the HLA-A*02 cohort, pNCL-01 and pNCL-02 were the most potent inducers of IFN-γ^+^ production, ranking highest and second highest, respectively, among the four candidate peptides (**Fig. 2E and F**). Conversely, in the HLA-B*15 group, pNCL-03, followed by pNCL-04 and pooled peptides, significantly induced the IFN-γ^+^ production compared to negative controls, which included both PBMCs pulsed without any peptide and target cancer cells alone (**Fig. 2K and L**). Notably, pNCL-pulsed PBMCs showed no IFN-γ production at day 0 (**Supplementary Fig. S2**). These findings indicate that pNCL-01 and -02 effectively elicited NCL-specific T cell responses in HLA-A*02 PBMCs, while pNCL-03 and pNCL-04 were capable of activating NCL-specific T cell responses in HLA-B*15-positive PBMCs.

### Upregulation of PD-1 on pNCL stimulated T cells

To further investigate the involvement of the PD-1/PD-L1 axis, PD-1 expression was evaluated on NCL peptide-stimulated T cells. PBMCs from HLA-A*02 and -B*15 HDs were stimulated with pNCL-01 to -04 and expanded for 21 days with three rounds of re-stimulation** (Supplementary Fig. S3)**. Flow cytometric analysis demonstrated an increased frequency of PD-1-expressing T cells within the CD3⁺ population compared to unpulsed controls. In the HLA-A*02 group, baseline PD-1 expression in unpulsed cells was low (1.26%), comparable to the isotype control (1.02%). Stimulation with pNCL-01 and pNCL-04 markedly increased PD-1 expression to 89.6% and 100.0%, respectively, whereas pNCL-02 and pNCL-03 showed minimal induction (1.49% and 1.43%) **(Supplementary Fig. S3A)**. In contrast, in the HLA-B*15 group, unpulsed cells exhibited higher baseline PD-1 expression (9.26%) compared with the isotype control (2.00%). Upon peptide stimulation, pNCL-01, pNCL-02, and pNCL-04 induced substantial PD-1 upregulation to 48.5%, 45.8%, and 49.8%, respectively, whereas pNCL-03 remained low (1.1%) **(Supplementary Fig. S3B)**.

### Cytotoxicity activity of pNCL-pulsed PBMCs against NCL-positive TNBC cell lines

To identify viable target cancer cells, the expression profiles of NCL and PD-L1 in MDA-MB-231 and HCC70 TNBC cell lines, as well as in MCF-10A noncancerous cells, were assessed through flow cytometry, western blot, and immunofluorescence staining (**Supplementary Fig. S4**). Both flow cytometry and western blot analyses revealed elevated levels of NCL and PD-L1 in both MDA-MB-231 and HCC70 cells, in contrast to negligible expression in MCF-10A cells (**Supplementary Fig. S4A and B**). Immunofluorescence staining revealed NCL presence in both the nucleus and cytoplasm, while PD-L1 localization was observed in the cytoplasm and cell membrane (**Supplementary Fig. S4C**).

**Figure 3:**
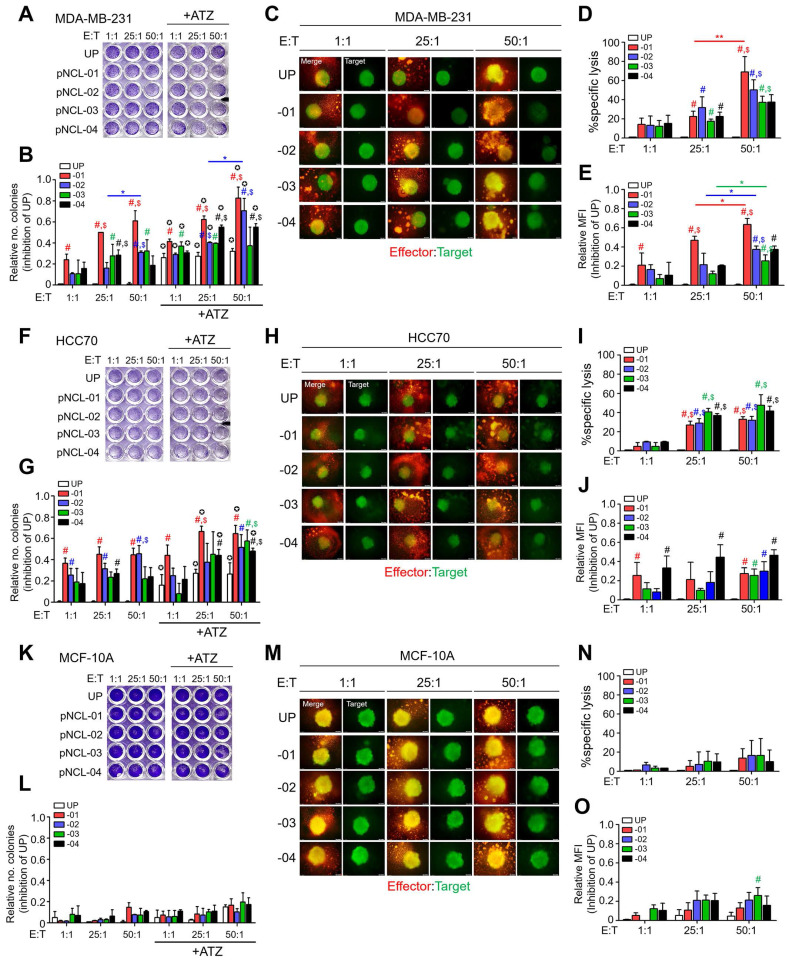
The 2-D and 3-D cytolytic efficacy of nucleolin short peptide-specific cytotoxic T cells derived from healthy donors HLA-A*02 against cancer cells, with and without atezolizumab treatment. (A-E) Targeting MDA-MB-231 cells. **(F-J)** Targeting HCC70 cells. **(K-O)** Targeting MCF-10A cells. Results from 2-6 independent experiments are reported. # = compared to unpulsed condition (UP) (of each ratio); $ = compared to 1:1 ratio (of each peptide); and µ = compared to untreated ATZ (of each ratio). Bars show mean ± SD. Statistical significance: # compared to UP; *, *P* ≤ 0.05; **, *P* ≤ 0.01; determined using a One-way ANOVA followed by the Tukey test.

**Figure 4:**
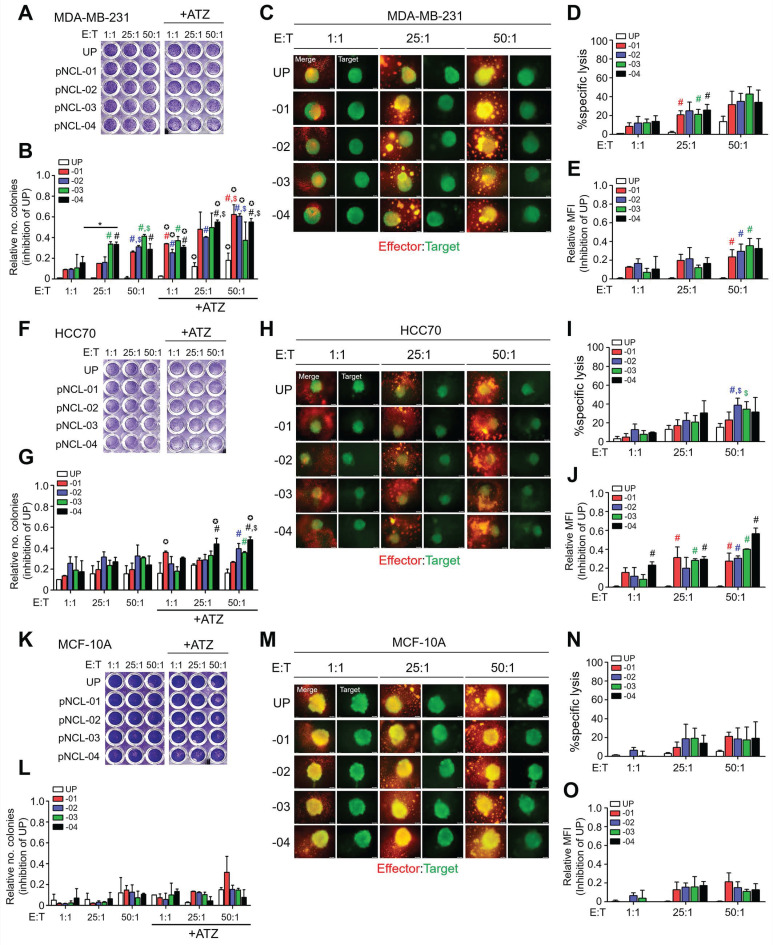
Assessment of 2-D and 3-D cytolytic activities of nucleolin short peptide-specific cytotoxic T cells from HLA-B*15 healthy donors (HDs), with and without atezolizumab treatment. (A-E) Target: MDA-MB-231 cells. **(F-J)** Target: HCC70 cells. **(K-O)** Target: MCF-10A cells. Results from 2-6 independent experiments are reported. # = compared to unpulsed (UP) condition (of each ratio); $ = compared to 1:1 ratio (of each peptide); and µ = compared to untreated ATZ (of each ratio). Bars show mean ± SD. Statistical significance: *, *P* ≤ 0.05; **, *P* ≤ 0.01; determined using a One-way ANOVA followed by the Tukey test.

To further validate the NCL specificity of the pNCL-pulsed PBMCs-derived NCL-specific T cells, NCL^+^/PD-L1^+^ HLA-A*02 positive MDA-MB-231 cells and NCL^+^/PD-L1^+^ HLA-B*15 positive HCC70 cells were employed as target cancer cells in a cytotoxicity assay (**Fig. 3 and Fig. 4**). A 24 h incubation involving three HLA-A*02-PBMCs with the MDA-MB-231 and HCC70 cell lines, along with the MCF-10A non-cancerous cell line, allowed for the measurement of cell death. This was quantified both by the number of colonies and the percentage of specific cell lysis utilizing mWasabi and CMRA fluorescence-labeled viable cells (**Fig. 3**). NCL-specific HLA-A*02 T cells, activated with the four peptides, exhibited a significant increase in the percentage of specific cancer cell lysis across ratios of 1:1, 25:1, and 50:1 against HLA-A*02-restricted MDA-MB-231 cells in a 2-D culture, displaying a dose-dependent fashion (**Fig. 3A and B**) and observed in 3-D culture as well (**Fig. 3C-E**). Similar dose-dependent findings were recorded at ratios of 25:1 and 50:1 for HLA-B*15-restricted HCC70 cells in 2-D culture (**Fig. 3F and G**) and in 3-D culture (**Fig. 3H-J**). Notably, cytotoxic activity against MCF-10A cells was only observed at a ratio of 50:1 (**Fig. 3K and L** for 2-D culture and **Fig. 3M-O** for 3-D culture).

Interestingly, T cells that were activated with pNCL-01 (illustrated by the red bar in **Fig. 3A and B**) demonstrated effective killing capabilities against MDA-MB-231 cells at a 1:1 ratio. Incorporation of the ATZ anti-PD-L1 antibody into the co-culture of cancer cells with NCL-specific T cells resulted in significantly enhanced death of the target cancer cells compared to NCL-specific T cells alone, as observed in both MDA-MB-231 (**Fig. 3A and B**) and HCC70 cells (**Fig. 3F and G**). These findings imply that pNCL-pulsed PBMCs yield NCL-specific T cells having high destructive activity against NCL/PD-L1-positive TNBC cells, an effect that was augmented by the ATZ anti-PD-L1 antibody. Particularly noteworthy is the HLA-A*02-restricted MDA-MB-231 scenario, where all four peptides prompted similar extents of cytotoxicity. However, pNCL-01 induced greater cell death compared to other pNCL treatments or unpulsed T cells, with statistical significance (*P* < 0.05; **Fig. 3A-E**).

Moreover, the cytotoxic effects exhibited by NCL-specific T cells derived from three healthy donors with HLA-B*15:01 and -B*15:02 genotypes against NCL/PD-L1-positive MDA-MB-231 and HCC70 TNBC cells highlighted a significant variance in the percentage of specific cell lysis at various effector-to-target ratios (1:1, 25:1, and 50:1), significantly differing markedly from the results seen with unpulsed T cells in both MDA-MB-231 (**Fig. 4A and B** for 2-D culture, and **Fig. 4C-E** for 3-D culture) and HCC70 cells (**Fig. 4F and G** for 2-D culture, and **Fig. 4H-J** for 3-D culture). Notably, the efficacy of specific cell lysis at these ratios was only observed against NCL-negative MCF-10A cells at the highest E:T ratio of 50:1 (**Fig. 4K and L** for 2-D culture, and **Fig. 4M-O** for 3-D culture). These findings underscore the ability of all tested NCL peptides to effectively orchestrate NCL-specific T cell-mediated cytotoxicity against both HLA-A*02-restricted MDA-MB-231 and HLA-B*15-restricted HCC70 cells.

### Candidate NCL peptides exhibited immunogenicity across TNBC patients with different HLAs

Utilizing the *in silico* tools NetMHC, NetMHCpan, NetMHCcons, NetCTLpan, and PickPocket, we investigated the potential for NCL peptides likely bound with different diverse HLA-A, HLA-B, and HLA-C alleles in a cohort of 10 TNBC patients (**Supplementary Tables S2-12**). Specifically, between 524-532 peptide sequences, notably, pNCL-04 (YAFIEFASF) demonstrated broad HLA-binding capacity, including representative alleles from HLA-A (e.g., A*24:02, A*24:10), HLA-B (e.g., B*13:01, B*15:02, B*35:01), and HLA-C (e.g., C*01:02, C*03:03, C*07:02), supporting its potential for cross-HLA immunogenicity (see **Supplementary Table S13** for the full list). The pNCL-04 may share immunogenicity across multiple HLA types in these ten patients (**Supplementary Table S13**), indicating its potential utility in TNBC treatments targeting a wide range of HLA alleles.

### Induction of effector peptide-pulsed T cells from PBMCs of TNBC patients

**Figure 5:**
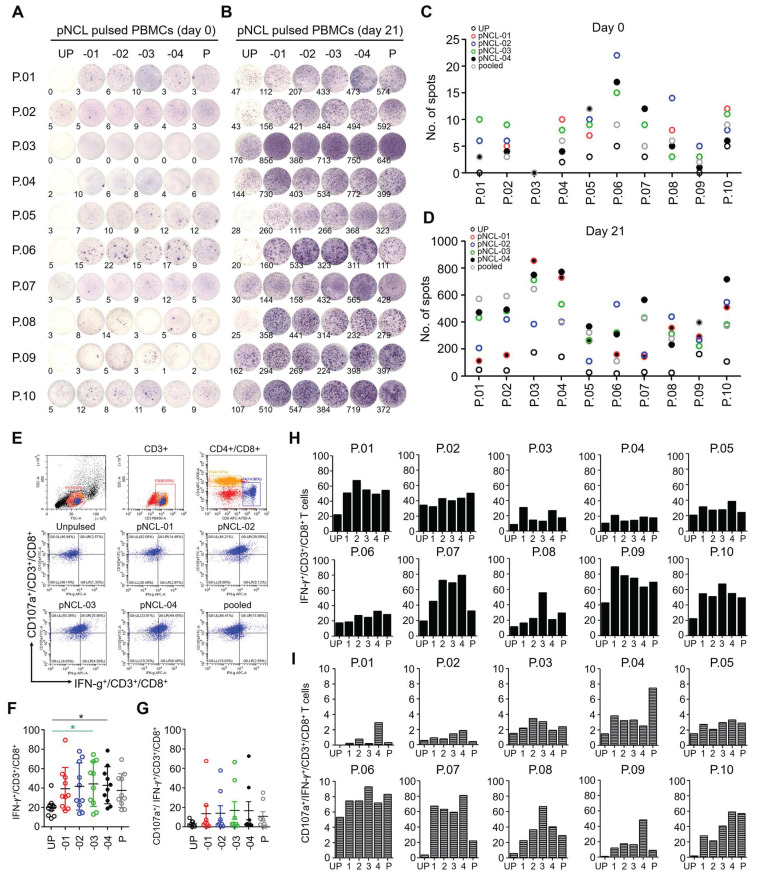
** Quantitative evaluation of the immune response: interferon-gamma (IFN-γ) production and spot-forming cells per 1 x 10^5^ cells. (A-D)** An ELISpot assay traced IFN-γ spot-forming units in NCL peptide-stimulated PBMCs from 10 TNBC patients, assessed at initiation (day 0) and after 3 weeks (day 21) of culture. **(E-I)** Flow cytometric analysis determined the intracellular cytokine expression of CD107a and IFN-γ, alongside the phenotyping of CD3^+^, CD4^+^, and CD8^+^ T cells, **(E-G)** presenting pooled data; and **(H-I)** showing results from a single experiment (n = 1). Bars show mean ± SD. Statistical significance: *, *P* ≤ 0.05; determined using a Two-tailed unpaired *t*-test. P, pooled NCL peptides; P. (number): patient identifier; UP, unpulsed peptide.

PBMCs from 10 patients with TNBC were stimulated with effector peptides pNCL-01, -02, -03, and -04 at the onset (day 0) and at day 21 of culture, followed by IFN-γ assessment (**Fig. 5A-D**, each data point represents an individual patient). On day 0, NCL peptide activation revealed NCL-specific IFN-γ spots in seven patients (P.01, P.02, P.03, P.06, P.07, P.08, and P.09) displaying elevated NCL protein expression levels in their tumor tissues (**Fig. 5A and C**; **Supplementary Table S14**;** Supplementary Fig. S5**). By day 21, robust high numbers of IFN-γ spots were elicited across all four NCL peptides (**Fig. 5B and D**). Notably, pNCL-04 stimulation generated a significant number of IFN-γ spots in the PBMCs of seven patients (P.01, P.02, P.04, P.05, P.07, P.09, and P.10), mirroring immunogenicity scores predicted by computational algorithms (**Supplementary Table S2**; **Fig. S6B, C, D, and F,** each data point represents an individual patient). Stimulation with pNCL-03 yielded the second highest number of IFN-γ production, surpassing responses in all other groups and unpulsed T cells (**Fig. 5B, D, and F**), indicating that pNCL-04 and pNCL-03 particularly specifically enhance NCL-specific T cell responses.

Moreover, the relationship between NCL expression levels in patient tissues and IFN-γ production following exposure to pNCLs on day 0 and day 21 was examined using Pearson analysis (**Supplementary Fig. S6-7**). On day 0, a positive correlation was identified between NCL expression in clinical cancer samples and IFN-γ production (**Supplementary Fig. S6**; pink line). By day 21, a positive correlation between NCL expression level in clinical patients' tissues and IFN-γ production was found, in which the positive relationship persisted notably for pNCL-03 and pNCL-04 (**Supplementary Fig. S6D and E**; black line). Further analysis, employing an immunohistochemistry score cutoff of ≥ 6, revealed a consistent positive correlation across all pNCLs related to NCL expression level in clinical cancer samples and IFN-γ production, both on day 0 and day 21. Especially, significant positive correlations were found in pNCL-03 and pNCL-04 after 21 days of pNCL-stimulated PBMCs of TNBC patients who have high NCL in their tissues (**Supplementary Fig. S7D and E**; black line), emphasizing the potential immunotherapeutic value of these peptides in high-NCL expressing TNBC cases.

To ascertain if HLA-A*02-restricted and HLA-B*15-restricted NCL peptides could activate PBMCs from NCL-positive patients, the expression of the degranulation marker CD107a and the activation marker CD69 was evaluated upon peptide stimulation. Additionally, the immunogenic potential of these NCL peptides was thoroughly assessed (**Fig. 5E-I**). After an 8-h resensitization period, intracellular IFN-γ production by CD8^+^ T cells (CD3^+^/CD8^+^/IFN-γ^+^) from TNBC patients was quantified using flow cytometry (**Fig. 5F and H**). The response of NCL-specific T cells from different patients under the same peptide stimulation was varied. The CD3^+^/CD8^+^/IFN-γ^+^ T cells were generated from all NCL peptide stimulation among different patients (**Fig. 5F and H**).

Compared to the negative control group, stimulation by all NCL peptides led to an increase in CD3^+^/CD8^+^/IFN-γ^+^ T cells (**Fig. 5H**), with a significant upregulation observed in cells stimulated by pNCL-03 and pNCL-04 after the 21-day culture period (**Fig. 5F**). To further examine the epitope specificity of the cytotoxic response induced by these peptide-activated PBMCs, CD3^+^/CD8^+^/CD107a^+^/IFN-γ^+^ T cells were consistently generated across all NCL peptide stimulations as opposed to the negative control or unpulsed T cells (**Fig. 5G and I**). However, significant statistical correlation was constrained by the limited follow-up time of the patient cohort (**Fig. 5G and I**; n = 1). In addition, CD4⁺ T cell responses were also evaluated, IFN-γ production by CD4⁺ T cells (CD3⁺/CD4⁺/IFN-γ⁺) was clearly increased in all peptide-stimulated conditions compared with the unpulsed control. All pNCL induced the strongest CD4⁺ T cell responses. The pooled peptide condition also showed an elevated response (**Supplementary Fig. S9**). These results demonstrate that CD4⁺ T cells are actively engaged upon NCL peptide stimulation and may contribute to the overall immune response in this PBMC-based system, although CD8⁺ T cells remain the primary cytotoxic effectors. The presence of cytotoxic CD4⁺ T cells observed in our study is consistent with recent reports demonstrating that CD4⁺ T cells can acquire direct cytotoxic functions and contribute to anti-tumor immunity. Notably, tumor-specific cytolytic CD4⁺ T cells have been shown to directly kill cancer cells through granzyme-dependent mechanisms, supporting their functional relevance beyond classical helper roles. 46 In addition, accumulating evidence indicates that cytotoxic CD4⁺ T cells represent an important effector population in anti-tumor immune responses and may act synergistically with conventional CD8⁺ T cells. 47

## Discussion

TNBC is a critical public health concern worldwide due to its aggressive nature, tendency for early recurrence, and poor prognosis. 1, 48 Its heterogeneity in lack of hormone receptor expression 2 makes chemotherapy the primary standard systemic treatment across neoadjuvant, adjuvant, and metastatic scenarios. 49 Despite extensive research, a specific target for chemotherapy for TNBC has not been identified. 49 However, recent advancements in sequencing, immunopeptidomics, and computational analysis facilitate the prediction of TAA peptides. 50, 51 Targeting these tumor antigens, by stimulating CD8^+^ cytotoxic and CD4^+^ helper T cell responses, has achieved effective anti-cancer immunity with minimal adverse effects. 6-8 In particular, HER-1 and IGF-1R peptides-pulsed PBMCs demonstrated significant anti-tumor efficacy and inhibited tumor growth in MDA-MB-231 TNBC cells. 5 Despite over 30 peptides having been processed safely to be effective in TNBC clinical trials, approval remains pending. 12 One important point is that the identification of an ideal universal TAAs remains a significant challenge. The perfect antigen should be highly expressed in cancers but minimally in normal tissues, be involved in tumorigenesis to avoid antigen variation or depletion, and must include epitopes that bind to HLA molecules. Furthermore, it should be recognized by the T cell repertoire and elicit specific T cell responses. 52, 53 NCL has recently been identified as exhibiting high expression correlated with poor outcomes in TNBC patients, suggesting its potential as a specific therapeutic target. 20 We examined PBMCs from six healthy donors and 10 TNBC patients with varying NCL expression to assess whether four HLA-restricted NCL peptides (pNCL-01 to -04) could induce NCL-specific T cells. These T cells effectively killed high PD-L1/NCL-positive TNBC cells (MDA-MB-231 or HCC70), especially when combined with atezolizumab, compared to low PD-L1 controls. T cells from high NCL patients produced elevated IFN-γ upon peptide stimulation. Notably, pNCL-04 bound to 20 common HLA class I alleles, supporting its broad applicability. The predicted broad HLA-binding profile of pNCL-04 may be explained by its structurally permissive sequence, particularly the presence of hydrophobic/aromatic residues at key anchor positions that can be accommodated by peptide-binding grooves of multiple HLA class I alleles. MD simulations further suggested that pNCL-04 maintains stable groove occupancy, compatible P2 to P9 anchor spacing, and adaptable side-chain orientations, supporting its potential for cross-allelic presentation. Nevertheless, these in silico findings indicate binding permissiveness rather than equivalent affinity across all alleles and require further experimental validation. Moreover, while the peptide may exhibit favorable binding energy *in silico*, its actual cell-surface stabilization and presentation, as demonstrated by the T2 assay, may be insufficient to support effective antigen presentation in the HLA-A*02 context. In addition, the peptide may not be efficiently processed and presented within the natural HLA ligand repertoire, further limiting its ability to induce a robust immune response. These findings are consistent with our functional data showing relatively weak responses in HLA-A02-positive donors. In contrast, pNCL-04 demonstrated strong immunogenicity in HLA-B15-positive healthy donors and TNBC patients, supporting its primary functional relevance in this context, in agreement with its original prediction and selection. These findings highlight the potential of NCL peptides for both vaccine development and T cell-based immunotherapy in high-NCL TNBC patients.

Peptide-pulsed PBMC-based immunotherapy has become one of the hot topics in research targeting therapies for solid tumors, partly due to the comprehensive array of antigen-presenting cells within PBMCs, including dendritic cells (DCs), B cells, monocytes/macrophages, and platelets, alongside its practicability. 54 Additionally, PBMC isolation is cost-effective, rapid, and straightforward to execute. 54 In the present investigation, whole PBMCs were used as effector cells to better reflect a physiologically relevant immune context that includes interactions between T cells and antigen-presenting cells. Importantly, our functional analyses consistently demonstrated that antigen-specific responses, including IFN-γ production, were predominantly observed in CD8⁺ T cells (CD3⁺/CD8⁺/IFN-γ⁺), supporting their primary role as cytotoxic effectors in this system. Moreover, we observed that the % CD3⁺/CD8⁺/IFN-γ⁺ T cells (41.84 ± 10.63%, **Fig. 5**) were much higher than those of CD3⁺/CD4⁺/IFN-γ⁺ T cells (26.37 ± 9.12 %, **Supplementary Fig. S9**). This is partially implying the impact of CD8+ T cells as key effector cells to destroy cancer cells. HLA class I-restricted NCL peptides were utilized to directly activate PBMCs, similarly to the previous reports. 55 Sequencing-based algorithms were deployed to predict NCL candidate peptides that have a high binding affinity to target cancer cell lines' HLA-A*02 molecule on target MDA-MB-231 cells or HLA-B*15 on target HCC70 cells and individual TNBC patients. The generation of NCL epitope peptide-pulsed PBMCs stimulated NCL-specific T cells which demonstrated strong potent anti-tumor immune responses against NCL-positive TNBC cells. This response was observed not only in healthy PBMCs, but also TNBC patients PBMCs with high NCL level in TNBC tissues.

To the best of our knowledge, no T cell epitopes of NCL have been documented thus far. In this study, we proposed four candidate NCL peptides, including: pNCL-01 (_15-23_KMAPPPKEV) and pNCL-02 (_488-496_VLSNLSYSA) for HLA-A*02; and pNCL-03 (_486-494_TLVLSNLSY) and pNCL-04 (_524-532_YAFIEFASF) for HLA-B*15, using a reverse immunology strategy. 56, 57 This comprehensive approach entailed a four-step process. Firstly, by using computer-based epitope prediction from the amino acid sequence of NCL, ≥ 3 from five *in silico* computational platforms, including NetMHC, NetMHCpan, NetMHCcons, NetCTLpan, and PickPocket, we identified NCL peptides with high binding affinity to HLA-A*02 and HLA-B*15. Notably, pNCL-01 and pNCL-02 showed a high affinity for HLA-A*02, commonly found worldwide, 21, 22 while pNCL-03 and pNCL-04 demonstrated a high affinity for HLA-B*15, commonly detected in the Asian population. 23 Secondly, a peptide-binding assay confirmed the affinity interaction between varying concentrations of peptides and the HLA molecule, which was successfully executed for HLA-A*02 on MDA-MB-231 cells due to the unavailability of HLA-B*15 T2 cells. Thirdly, MD simulations corroborated the peptide-HLA binding, revealing a higher binding free energy for each NCL peptide with HLA-A*02:01 or HLA-B*15:01 compared to positive controls from PDB: 5C07 43 and PDB: 1XR8, 44 respectively. Moreover, a side chain of the COOH-terminal anchor residue of all pNCL oriented into the HLA binding groove, showing a specific distance, ranging from 15 Å to 21 Å, between the two anchor residues, P2 to P9. 58

Finally, we evaluated the stimulation of isolated T cell response against the predicted NCL peptides *in vitro* and tested the functional effect of NCL-specific T cells against TNBC target cells expressing NCL endogenously. This approach enabled the collection and analysis of blood samples from 6 healthy donors and 10 TNBC patients. We then investigated the capability of the 4 candidate NCL peptides, when restricted to individual HLAs, to induce NCL-specific T cells. Notably, these 4 NCL immunogenic peptides exhibited high binding affinity and represented their most common binding, particularly with HLA-A*02, which ranks as the fifth most common HLA allele in Asia and the Thai population. 59 The pNCL-01 exhibited the highest binding affinity among all screened epitopes, suggesting a strong potential for stable complex formation with the corresponding HLA molecule. High-affinity peptide-MHC interactions are a critical determinant of effective antigen presentation, as they promote peptide-MHC complex stability on the surface of APCs, thereby enhancing recognition by cytotoxic CD3^+^CD8⁺ T cells. 60 Such suggests that it could serve as a tumor-associated epitope capable of activating T cells to selectively recognize and eliminate cancer cells expressing the corresponding antigen. 61

The 4 NCL peptides were shown to evoke an antigen-specific CD8^+^ T cell response, consequently, upon stimulation with NCL peptides, an increase in activated T cell markers was observed, specifically in CD107a and IFN-γ, thereby identifying the activation of CD3^+^CD8^+^ cytotoxic T cells. This is in line with research in chronic lymphocytic leukemia, where CD107a was highlighted as a cellular marker for activated T cells, underscoring the general applicability and significance of these markers in identifying effector T cells in cancer immunity. 62, 63

It is noteworthy that PBMCs from breast cancer patients, exhibiting high levels of NCL in their tumor mass, present elevated levels of NCL-specific T cells compared to those with lower NCL expression in cancer tissue. Significantly, the high immunogenicity of the four NCL peptides became evident at day 21 after three cycles of restimulation. These findings underscore the potential of utilizing HLA-restricted overexpressed tumor antigen peptides as a peptide vaccine to activate tumor-specific T cells for breast cancer patients. The 9-mer HLA binding NCL peptides demonstrated a high immunogenic potential in TNBC patients in consistent with previous findings. 55 It was found that pNCL-04 seems to be a shared HLA-restricted peptide of all 10 TNBC patients showing immunogenic potential-related scores. This shared immunogenic peptide represents a potential antigen epitope that is found in multiple cancer patients, not unique to one individual (unlike neoantigens), and thus is "shared" across patients with similar HLA types. 64, 65 As hypothesized, the shared immunogenic pNCL-04 successfully activated PBMCs from all 10 TNBC patients, transforming them into NCL-specific T cells that released high levels of IFN-γ production for each patient. While functional activation (IFN-γ production) was observed in 7 patients, *in silico* predictions demonstrated that pNCL-04 has binding potential across multiple HLA alleles present in all 10 patients, supporting its broad applicability from an antigen-presentation perspective. These findings affirm the feasibility of employing NCL peptides to be proposed as a common peptide for patients with high NCL expression. This concept is supported by research into other cancers, such as the ability of human papillomavirus type 18 E7 single peptides to bind to four different HLA class I alleles including A*02:01, A*24:02, A*11:01, and A*33:03 which are among the most frequent alleles in the human race. 66

Of note, this present study has some limitations. The first is the absence of TNBC patient-derived target cancer cells for use in killing assays to evaluate the cancer cell killing efficacy of the obtained NCL-specific T cells. Additionally, IFN-γ production did not consistently correlate with NCL expression levels in cancer tissues. However, coordinated expression of NCL in original tissues was linked to an enhanced T cell response upon activation with pNCL-03 and -04. Consistent with previous *in vitro* and phase I/IIa clinical trials, various agents targeting NCL, such as aptamers AS1411, anti-NCL antibodies, and the antagonistic pseudo-peptide N6L, demonstrated anti-tumor activity in several cancers, including advanced solid tumors, acute myeloid leukemia, metastatic refractory renal cell carcinoma, carcinoid/neuroendocrine tumors, and lung cancers. 67-71 Although T cell function was assessed using IFN-γ production and CD107a expression, a more comprehensive evaluation of T cell polyfunctionality, including TNF-α, IL-2, and cytotoxic effector molecules such as perforin and granzyme B, would further strengthen the functional characterization and should be addressed in future studies. Moreover, we evaluated PD-L1 expression in the resected tumor tissues obtained at the time of surgery. Importantly, none of the 10 TNBC patients had received any neoadjuvant therapy. We acknowledge that peripheral blood samples used for PBMC isolation were collected after surgery, during which some patients had received adjuvant therapy, including chemotherapy and/or radiotherapy. This adjuvant treatment may potentially influence immune cell composition and responsiveness. 72, 73 Though some chemotherapeutic agents (i.e. Doxorubicin and Docetaxel) 73, 74 and radiation 75 have been reported as the enhancer for anti-tumor immune responses, no clear association between the adjuvant treatment history and the magnitude of antigen-specific T cell responses in our study. However, all PBMC samples were processed under identical experimental conditions, and the pNCL-induced immune responses were consistently observed across patients (**Supplementary Table S14**). Importantly, the responses were NCL-specific, rather than indicative of generalized immune activation, suggesting that functional T cell reactivity remained preserved suggesting that the stimulatory effect of the peptides remains detectable despite prior adjuvant treatment. Due to the limited sample size, we did not perform subgroup analyses based on clinical characteristics such as PD-L1 expression status or prior treatment history. Our preliminary review of the available clinical follow-up data Up to our final check, all 10 TNBC patients remain alive and clinically stable in a range of 127.44 - 165.00 months overall survival (**Supplementary Table S14**). However, due to the limited sample size and relatively short follow-up period, we are currently unable to establish a meaningful correlation between *in vitro* T cell responses to NCL peptides and long-term clinical outcomes (e.g., disease-free survival or response to subsequent therapies).

The advent of cancer immunotherapy, particularly, immune checkpoint inhibitors, has fundamentally transformed the approach to treating diseases, driven by the elevated expression of PD-L1 in TNBC. 76, 77 ATZ and pembrolizumab have received approval for use in clinical trials for the advanced stage of PD-L1-positive TNBC patients; nevertheless, only approximately 30% of these patients had benefit from these treatments. 48, 78, 79 Hence, from this perspective, there was a strong rationale for exploring the imperative need to investigate potential immunotherapeutic targets in TNBC to identify therapeutic avenues that could be integrated with existing treatments to enhance TNBC patient survival rates. The application of anti-PD-L1 antibodies has been demonstrated herein to sensitize PD-L1/NCL-positive cancer cells to peptide-activated NCL-specific T cells, facilitating targeted cancer cell destruction without affecting normal epithelial mammary cells. Notably, ATZ alone demonstrated a measurable anti-tumor effect in our system **(Fig. 3 and 4)**, suggesting that, in addition to its established role in restoring T cell activity via PD-1/PD-L1 blockade, ATZ may also exert tumor-intrinsic effects. Previous studies have reported that PD-L1 can contribute to cancer cell survival and resistance signaling pathways, independent of immune modulation through the activation of oncogenic signaling pathways (PI3K/AKT and MAPK/ERK), regulation of epithelial-mesenchymal transition (EMT), and resistance to apoptosis mechanisms. 80 However, the enhanced cytotoxicity observed in the combination setting indicates that T cell-mediated mechanisms remain the primary driver of anti-tumor activity. 71, 72 PD-1 expression was markedly increased on CD8⁺ T cells following stimulation with pNCL compared to unpulsed controls, indicating activation-associated upregulation and supporting the involvement of the PD-1/PD-L1 axis in this system. Repeated stimulation with NCL peptides led to a marked upregulation of PD-1 on CD8⁺ T cells, indicating the generation of antigen-experienced T cells under sustained antigen exposure. 73, 74 This observation provides a mechanistic rationale for combining NCL peptide-induced T cell responses with PD-L1 blockade. The increased PD-1 expression suggests that NCL-specific T cells are susceptible to PD-1/PD-L1-mediated inhibitory signaling, particularly in the context of PD-L1⁺ TNBC cells. In this setting, engagement of PD-1 by tumor-expressed PD-L1 may dampen T cell effector function, thereby limiting cytotoxic activity. Therefore, blockade of PD-L1 by ATZ is expected to relieve this inhibitory axis, restoring T cell function and enhancing anti-tumor responses. This is consistent with our findings showing improved cytotoxicity in the combination setting. Collectively, these data support a biologically relevant rationale for combining NCL peptide-induced T cells with PD-L1 blockade to overcome checkpoint-mediated suppression and improve therapeutic efficacy. 75

Although NCL-targeted T cells demonstrated minimized cytotoxicity toward MCF-10A cells *in vitro,* to ensure the on-target/off-tumor side effect, data from the The Human Protein Atlas data base revealed high NCL levels in several representative normal tissues including bone marrow hematopoietic cells, intestinal epithelial compartments, breast glandular cells, lung alveolar cells, liver hepatocytes, thyroid glandular cells, and cardiomyocytes (**Supplementary Fig. S8A and B**). In addition, the Human Protein Atlas summary indicates low tissue specificity at the RNA level, including GTEx-derived tissue RNA data **(Supplementary Fig. S8A and B)**. Of great interest, the expression level of NCL in these normal organs is lower than that in breast cancer tissue (**Supplementary Fig. S8C**). Though, the potential for on-target, off-tumor toxicity cannot be excluded, the killing effect of NCL-specific T cells on breast cancer cells are high and value to use. Moreover, future studies incorporating primary normal human cells and *in vivo* models are warranted to further assess safety and therapeutic windows. This study is limited by the lack of *in vivo* validation. Although *in silico, ex vivo*, and *in vitro* approaches demonstrated the immunogenicity and anti-tumor activity of NCL-derived peptides, these findings do not fully reflect the complexity of the *in vivo* tumor microenvironment. Future studies using appropriate animal models are required to validate therapeutic efficacy and safety *in vivo*. Moreover, in the present study, experiments were conducted using PBMCs from 6 HDs and 10 TNBC patients, which allowed us to consistently observe peptide-specific T cell responses across multiple individuals. However, we acknowledge that the sample size remains limited and that *in vivo* validation was not included, as the study was designed as an initial proof-of-concept investigation focusing on peptide identification and functional *in vitro* characterization. Future studies will aim to expand the patient cohort and incorporate appropriate *in vivo* models to further validate the therapeutic potential of NCL peptides in TNBC. This approach suggests a promising direction for augmenting the efficacy of current immunotherapeutic strategies for TNBC treatment.

## Conclusion

The significant discoveries from this study underscore the potential of NCL as a promising target for devising and refining alternative cancer treatments for TNBC patients. The successful identification of HLA class I-restricted NCL peptides and their capacity to activate NCL-specific T cells from PBMCs are demonstrated herein. Furthermore, this research sheds light on the influence of ICIs on T cells activated by NCL peptides, and the endeavors to establish NCL as a common peptide could vastly improve immunotherapeutic efficacy.

## Supplementary Material

Supplementary figures and tables.

## Figures and Tables

**Table 1:** Characterization of *in silico*-predicted 9-amino acid NCL epitopes restricted to HLA-A*02:01, HLA-A*02:17, HLA-B*15:01, and HLA-B*15:16

No.	HLA	Position	Peptide sequences	Name in this study	NetMHC		NetMHCpan		NetMHCcons		NetCTLpan	PickPocket
					Affinity (nM)	%Rank	Affinity (nM)	%Rank	Affinity (nM)	%Rank	%Rank	Affinity (nM)
1	A*02:01	488	VLSNLSYSA	pNCL-02	33.89	0.50 (SB)	65.24	0.82 (WB)	32.24	1.50 (SB)	1.50	0.63
2		15	KMAPPPKEV	pNCL-01	236.91	1.80 (WB)	109.03	0.04 (SB)	264.44	4.00 (WB)	1.50	0.60
3		567	SQPSKTLFV	-	201.33	1.60 (WB)	574.04	0.65 (WB)	261.59	4.00 (WB)	3.00	0.52
4		500	TLQEVFEKA	-	664.71	3.50	164.28	0.24 (SB)	280.65	4.00 (WB)	2.00	0.54
5		369	KVFGNEIKL	-	711.09	3.50	538.18	0.26 (SB)	1170.65	6.00	4.00	0.51
6		343	GMTRKFGYV	-	317.31	2.50	785.25	5.04	492.62	5.00 (WB)	2.00	0.63
7		497	ATEETLQEV	-	1544.20	5.00	2471.02	1.14 (WB)	2157.34	8.00	8.00	0.44
8		404	VTQDELKEV	-	3055.55	7.50	3974.56	1.55 (WB)	3911.67	10.00	16.00	0.40
9		363	ALELTGLKV	-	3017.38	7.50	3144.23	1.88 (WB)	2664.09	8.00	6.00	0.52
10		486	TLVLSNLSY	-	12252.01	18.00	18209.68	11.63	21040.32	32.00	9.00	0.17
11		524	YAFIEFASF	-	5774.27	11.00	11147.34	14.58	11479.16	32.00	7.00	0.23
1	A*02:17	15	KMAPPPKEV	pNCL-01	489.96	0.70 (WB)	2058.36	0.11 (SB)	1700.36	3.00	0.80 (<-E)	0.49
2		567	SQPSKTLFV	-	1153.38	1.20 (WB)	2599.76	0.50 (SB)	1283.42	3.00	2.00	0.52
3		63	VVVSPTKKV	-	2139.72	1.80 (WB)	16798.72	10.64 (WB)	4151.52	5.00	16.00	0.33
4		394	LLAKNLPYK	-	2313.20	1.90 (WB)	13125.66	8.03	11417.23	10.00	16.00	0.33
5		369	KVFGNEIKL	-	7023.73	4.50	2332.43	0.18 (SB)	1525.99	3.00	1.50	0.41
6		488	VLSNLSYSA	pNCL-02	14881.86	8.50	4084.42	1.82 (WB)	3954.23	5.00	3.00	0.49
7		500	TLQEVFEKA	-	19838.92	12.00	5018.57	1.04 (WB)	6610.93	7.00	5.00	0.43
8		337	AVVDVRIGM	-	23903.31	15.00	7531.16	1.20 (WB)	11986.87	15.00	16.00	0.36
9		308	NLFVGNLNF	-	29313.42	20.00	3979.03	1.29 (WB)	15541.08	15.00	2.00	0.29
10		453	EIDGRSISL	-	20090.36	27.00	16178.84	1.83 (WB)	6095.65	7.00	16.00	0.27
11		362	ALELTGLKV	-	4065.64	3.00	13940.25	3.51	2204.54	4.00	10.00	0.48
12		486	TLVLSNLSY	pNCL-03	19774.85	12.00	25913.51	8.82	24481.50	32.00	8.00	0.18
13		524	YAFIEFASF	pNCL-04	35560.78	28.00	9786.84	3.89	16316.49	15.00	3.00	0.09
1	B*15:01	524	YAFIEFASF	pNCL-04	20.24	0.17 (SB)	25.05	0.46 (SB)	20.03	0.10 (SB)	0.15 (<-E)	0.54
2		486	TLVLSNLSY	pNCL-03	65.70	0.60 (WB)	42.63	0.11 (SB)	80.87	1.00 (WB)	0.80 (<-E)	0.37
3		393	TLLAKNLPY	-	121.08	0.90 (WB)	121.61	0.478 (SB)	140.42	1.50 (WB)	1.50	0.354
4		566	RSQPSKTLF	-	342.94	1.80 (WB)	711.49	0.49 (SB)	319.56	3.00 (WB)	3.00	0.34
5		307	NLFVGNLNF	-	373.17	1.80 (WB)	174.11	0.67 (WB)	176.24	2.00 (WB)	0.80 (<-E)	0.37
6		517	NQNGKSKGY	-	708.28	3.00	864.17	0.26 (SB)	384.09	3.00 (WB)	4.00	0.10
7		15	KMAPPPKEV	pNCL-01	7850.23	12.00	5019.00	1.39 (WB)	8387.64	16.00	9.00	0.17
8		405	TQDELKEVF	-	2194.07	5.50	3301.28	0.58 (WB)	8387.64	16.00	16.00	0.29
9		337	AVVDVRIGM	-	2652.30	6.00	1829.61	1.76 (WB)	3307.72	9.00	16.00	0.23
10		370	KVFGNEIKL	-	12945.00	18.00	7696.15	1.99 (WB)	14175.55	32.00	16.00	0.21
11		488	VLSNLSYSA	pNCL-02	3113.83	6.50	3921.46	6.39	N/A	N/A	8.00	0.18
1	B*15:16	566	RSQPSKTLF	-	N/A	N/A	177.03	0.02 (SB)	79.57	0.80 (WB)	0.80 (<-E)	0.52
2		524	YAFIEFASF	pNCL-04	N/A	N/A	103.55	0.15 (SB)	105.42	1.00 (WB)	0.40 (<-E)	0.45
3		96	KTVTPAKAV	-	N/A	N/A	1491.66	0.76 (WB)	421.09	4.00 (WB)	4.00	0.36
4		370	KVFGNEIKL	-	N/A	N/A	1975.89	0.34 (SB)	869.37	6.00	6.00	0.36
5		15	KMAPPPKEV	pNCL-01	N/A	N/A	10018.80	1.36 (WB)	4454.00	32.00	10.00	0.19
6		337	AVVDVRIGM	-	N/A	N/A	7866.30	1.82 (WB)	2277.27	15.00	10.00	0.26
7		63	VVVSPTKKV	-	N/A	N/A	9112.13	1.82 (WB)	3529.57	15.00	16.00	0.25
8		396	LAKNLPYKV	-	N/A	N/A	5777.83	1.98 (WB)	4406.07	16.00	10.00	0.18
9		486	TLVLSNLSY	pNCL-03	N/A	N/A	6714.67	2.22	2751.98	15.00	4.00	0.24
10		404	VTQDELKEV	-	N/A	N/A	10618.62	2.56	3997.24	15.00	10.00	0.26
11		90	AATPAKKTV	-	N/A	N/A	11463.40	2.72	3150.53	15.00	32.00	0.27
12		488	VLSNLSYSA	pNCL-02	N/A	N/A	23716.53	2.77	21154.46	50.00	32.00	0.06

The binding score was obtained from the DTU Health Tech (http://www.cbs.dtu.dk/). N/A: not applicable; NetMHC: Threshold for strong binder (%Rank) 0.5; Threshold for weak binder (%Rank) 2; NetMHCPan: Threshold for strong binder (%Rank) 0.5; Threshold for weak binder (%Rank) 2.0; NetMHCcons: Threshold for strong binder (%Rank) 0.5; Threshold for strong binder IC50 < 500 nM; Threshold for weak binder (%Rank) 2.0; Threshold for weak binder IC50 < 2000 nM; NetCTLpan: Threshold for epitope identification (%Rank) 1.0; PickPocket: Prediction values IC50 < 500 nM are considered; SB: strong binding; WB: weak binding. Underlined boxes indicate candidate peptides that fulfilled at least three out of five selection criteria.

**Table 2:** Profile of NCL peptides restricted to HLA-A*02:01 and HLA-B*15:01

Peptide name (amino acid position)	Amino acid sequences	HLA	d[P2-P9]^a^ (Å)	Orientation of side chain^b^ (P9)	Δ*G*_SIE_ (kcal/mol)	Δ*G*_MM/GBSA_ (kcal/mol)	Δ*G*_MM/PBSA_ (kcal/mol)
pNCL-01 (15-23)	KMAPPPKEV	A*02:01	16.76±1.29	↓	-11.59±0.39	-10.50±1.20	-26.02±2.06
	KMAPPPKEV	B*15:01	17.55±0.54	↓	-11.41±1.30	-12.33±6.72	-22.25±6.47
pNCL-02 (488-496)	VLSNLSYSA	A*02:01	16.33±0.22	↓	-12.61±0.24	-11.62±3.45	-29.76±1.33
	VLSNLSYSA	B*15:01	17.19±0.20	↓	-14.57±0.59	-22.19±2.40	-30.12±1.68
pNCL-03 (486-494)	TLVLSNLSY	A*02:01	14.15±1.49	↓	-12.02±0.49	-12.37±5.00	-24.90±3.63
	TLVLSNLSY	B*15:01	18.26±0.24	↓	-15.18±0.32	-31.36±3.57	-31.45±3.12
pNCL-04 (524-532)	YAFIEFASF	A*02:01	17.43±1.41	↓	-12.47±1.45	-15.52±2.77	-28.19±1.54
	YAFIEFASF	B*15:01	18.82±0.29	↓	-13.28±0.71	-16.06±2.91	-25.22±1.14
PDB: 5C07^c^	YQFGPDFPIA	A*02:01	18.85±0.30	↓	-12.88±0.04	-14.61±1.20	-29.25±1.07
PDB: 1XR8^d^	LEKARGSTY	B*15:01	21.28±0.75	↓	-14.60±0.27	-23.28±4.17	-30.06±2.96

^a^The distance between P2 and P9 peptide residues and the orientation of the side chain at P9 are determined by the molecular dynamics simulation as described in **Figure. 1B** and **1F**. **Å**: Angstrom. ^b^Arrows indicate the orientation of P9 residue concerning the floor of the binding groove of HLA-A*02:01 and HLA-B*15:01. Δ*G*: the binding free energy; SIE: solvated interaction energy, MM/GBSA: molecular mechanics with Generalized Born surface area, and MM/PBSA: molecular mechanics with Poisson-Boltzmann surface area. ^c^5C07: The complex with HLA-A*02:01 carrying YQFGPDFPIA. 43 ^d^1XR8: The HLA-B*15:01 in complex with UbcH6 and Epstein-Barr Virus EBNA-3). 44

## Data Availability

The data supporting the findings of this study are available from the corresponding author upon reasonable request.

## Abbreviations

BCA: Breast cancer
HD: Healthy donor
HLA: Human leukocyte antigen
IFN-γ: Interferon-gamma
IL: Interleukin
MD: Molecular dynamics
NCL: Nucleolin
PBMCs: Peripheral blood mononuclear cells
PD-L1: Programmed death-ligand 1
TNBC: Triple-negative breast cancer
UP: Unpulsed
