# Supplementary material for: HLA class I-specific nucleolin peptides induce therapeutic T cells in triple-negative breast cancer patients
Source: Int J Biol Sci. 2026 Jul 20;22(12):6814–35. doi: 10.7150/ijbs.132389 (PMC13412292; doi:10.7150/ijbs.132389)
Supplement: Supplementary file 1 — Supplementary figures and tables. [file ijbsv22p6814s1.pdf]

Supplement Figure legends

Supplement Fig. S1. The percentage of CD3, CD4, and CD8 positive T cells from PBMCs of three healthy donors (HDs) with HLA-A*02 and three with HLA-B*15. Phenotypic characterization via flow cytometry of representative PBMCs samples, using CD3-eFluor450, CD4-Alexa Fluor700, and CD8-APC Cy7. Data representative as mean ± SD of 2- 3 independent experiments of each donor. Statistical analysis was performed using one-way ANOVA with Tukey's post hoc test. Bars show mean ± SD. HD: healthy donor, UP: unpulsed peptide, pooled: pooled NCL peptide.

Supplement Fig. S2. The IFN-γ production of pNCL at day 0 by ELISpot assay from PBMCs of two healthy donors (HDs) with HLA-A*02 and HLA-B*15. (A) HLA-A*02 and **(B)** HLA-B*15. PBMCs sensitized with PMA/Ionomycin were used as a positive control (PMA/Iono), and PBMCs sensitized with no peptide (unpulsed, UP) were used as the negative control. HD: healthy donor, UP: unpulsed peptide, pooled: pooled NCL peptide.

Supplement Fig. 3. PD-1 upregulation on $CD8^+$ T cells following NCL peptide stimulation across different HLA backgrounds. Representative flow cytometry dot plots showing PD-1 expression on $CD8^+$ T cells after stimulation with pNCLs. PBMCs from TNBC patients were stimulated with individual pNCLs (pNCL-01, pNCL-02, pNCL-03, and pNCL-04) or UP, with isotype control as baseline, of **(A)** HLA-$A02^+$ patient and **(B)** HLA-$B15^+$ patient. Numbers indicate the percentage of PD-1^+ cells within the $CD3^+$ T cell population.

Supplement Fig. S4. NCL and PD-L1 expression in breast cancer cell lines. (A) NCL and PD-L1 expression detected by flow cytometry and **(B)** western blot analysis. β-actin was used as a control. **(C)** Immunofluorescence staining of NCL (green color) and PD-L1 (green color) in MCF-10A, MDA-MB-231, and HCC70 cells. Nuclei were stained with Hoechst (blue color). n = 3, statistical analysis was performed using an unpaired two-tailed *t*-test. Scale bars, 20 µm.

Supplement Fig. S5. Immunohistochemistry staining of NCL in ten TNBC patients' tissues. [19] Scale bar = 400 µM (circle) and 60 µM (square).

Supplement Fig. S6. The correlation between NCL expression level in original tissues and IFN-γ production at day 0 (pink line) and 21 (black line) was analyzed by Pearson analysis.

Supplement Fig. S7. Pearson correlation analysis showed a correlation of NCL expression level in original tissues with IFN-γ production at day 0 (pink line) and 21 (black line), compared by low and high NCL scoring (cut off ≥ 6).

Supplement Fig. S8. **Expression profile of NCL across normal tissues and cancers. (A-B)** RNA and protein expression of NCL across human normal tissues. RNA expression (nTPM) and protein expression (immunohistochemistry-based score) are shown across multiple organ systems, indicating broad but variable baseline expression. RNA expression of NCL across cancer types using TCGA datasets. Box plots illustrate expression distribution across tumors, highlighting inter-tumor variability and context-dependent enrichment. **(C)** Differential expression of NCL in breast cancer (BRCA), comparing tumor (T, red box) and normal (N, grey box) tissues. Each dot represents an individual sample; box plots show median and interquartile range, indicating higher NCL expression in tumor tissues relative to normal controls.

Supplement Fig. S9. Induction of IFN-γ-producing $CD3^+$/$CD4^+$ T cells following NCL peptide stimulation in a PBMC-based system. (A) Representative flow cytometry plots showing gating strategy and intracellular IFN-γ production in $CD4^+$ T cells after stimulation with individual pNCLs (pNCL-01, pNCL-02, pNCL-03, pNCL-04), pooled peptides, or UP. PBMCs were first gated on $CD3^+$ T cells, followed by identification of $CD4^+$ and $CD8^+$ subsets, and subsequent assessment of IFN-γ expression within the $CD3^+$/$CD4^+$ population. **(B)** Quantitative analysis of IFN-γ^+ cells within the $CD3^+$/$CD4^+$ T-cell population across different stimulation conditions. Each dot represents an individual donor, and bars indicate mean ± SD.

Supplementary Tables

Supplementary Table S1. HLA typing of breast cancer cells, healthy donors and patients.

Supplementary Table S2. Summarize of candidate predicted NCL epitopes restricted to patients HLAs No. P.01-P.10.

Supplementary Table S3. Characterization of in silico predicted NCL epitopes restricted to HLA of patient No. P.01.

Supplementary Table S4. Characterization of in silico predicted NCL epitopes restricted to HLA of patient No. P.02.

Supplementary Table S5. Characterization of in silico predicted NCL epitopes restricted to HLA of patient No. P.03.

Supplementary Table S6. Characterization of in silico predicted NCL epitopes restricted to HLA of patient No. P.04.

Supplementary Table S7. Characterization of in silico predicted NCL epitopes restricted to HLA of patient No. P.05.

Supplementary Table S8. Characterization of in silico predicted NCL epitopes restricted to HLA of patient No. P.06.

Supplementary Table S9. Characterization of in silico predicted NCL epitopes restricted to HLA of patient No. P.07.

Supplementary Table S10. Characterization of in silico predicted NCL epitopes restricted to HLA of patient No. P.08.

Supplementary Table S11. Characterization of in silico predicted NCL epitopes restricted to HLA of patient No. P.09.

Supplementary Table S12. Characterization of in silico predicted NCL epitopes restricted to HLA of patient No. P.10.

Supplementary Table S13. Characterization of in silico and MD prediction of NCL epitopes restricted to patients' HLA.

Supplementary Table S14. Clinical data of breast cancer patients.

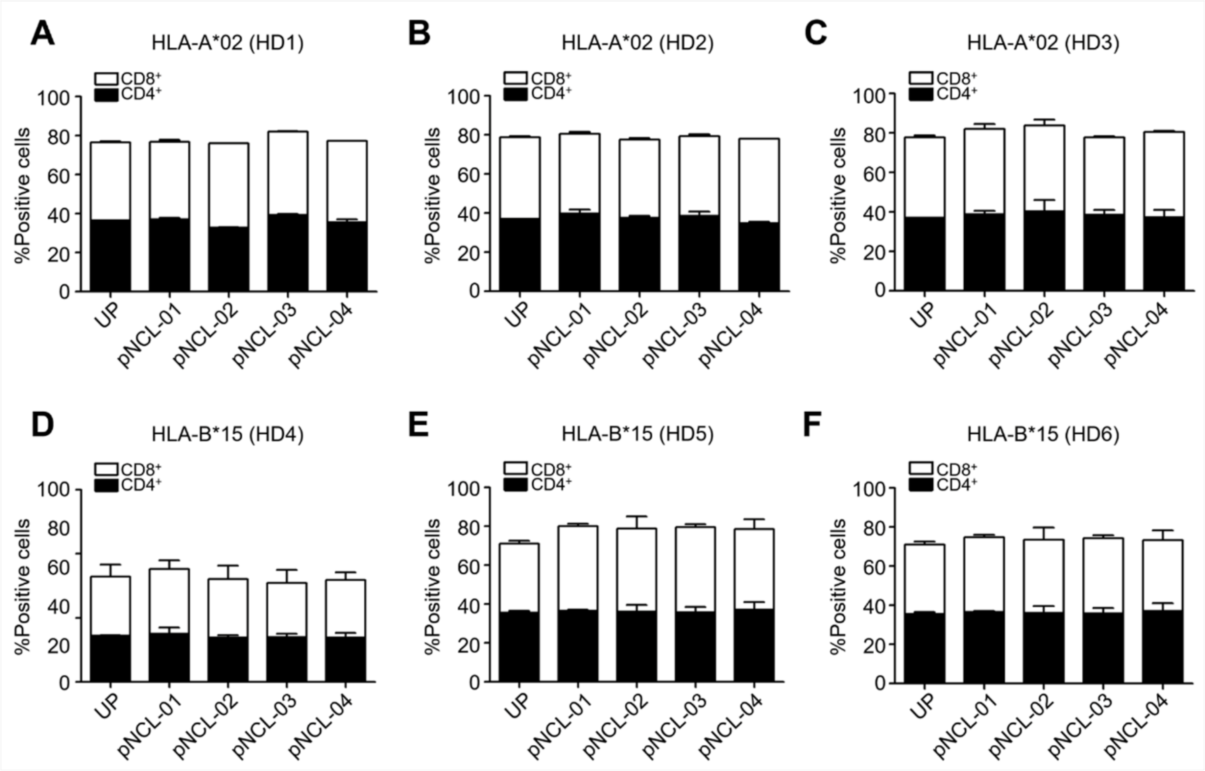

Supplementary Figure S1

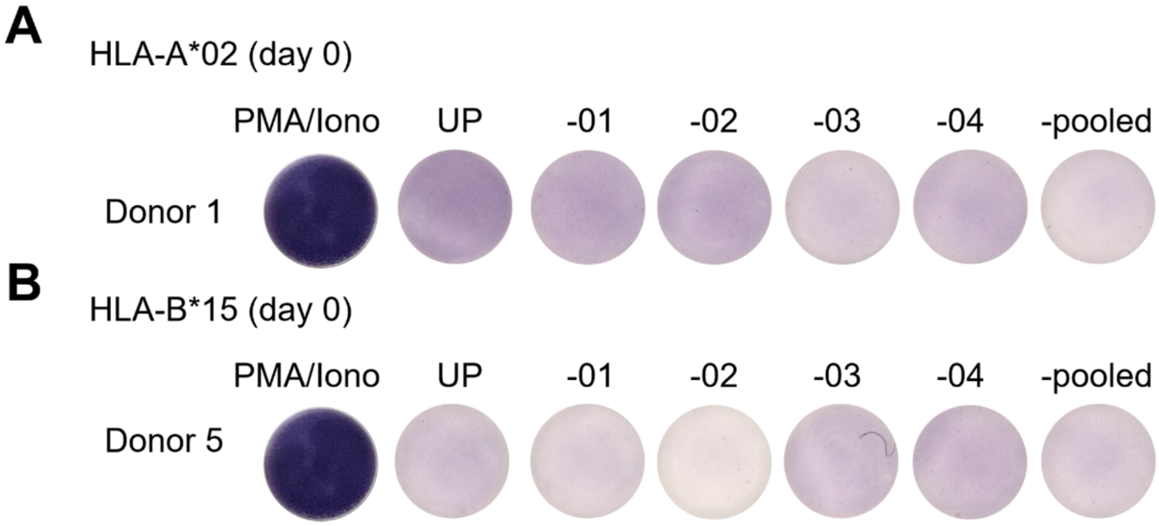

Supplementary Figure S2

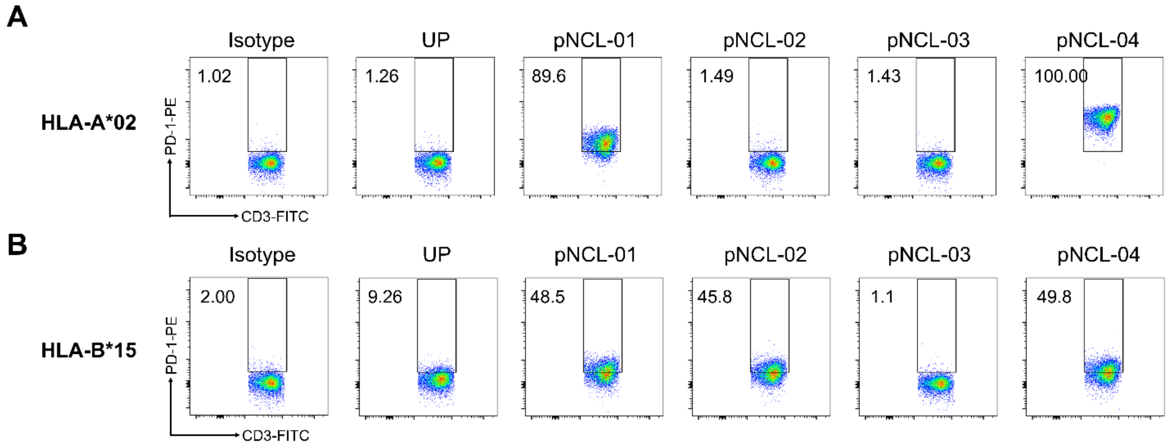

Supplementary Figure S3

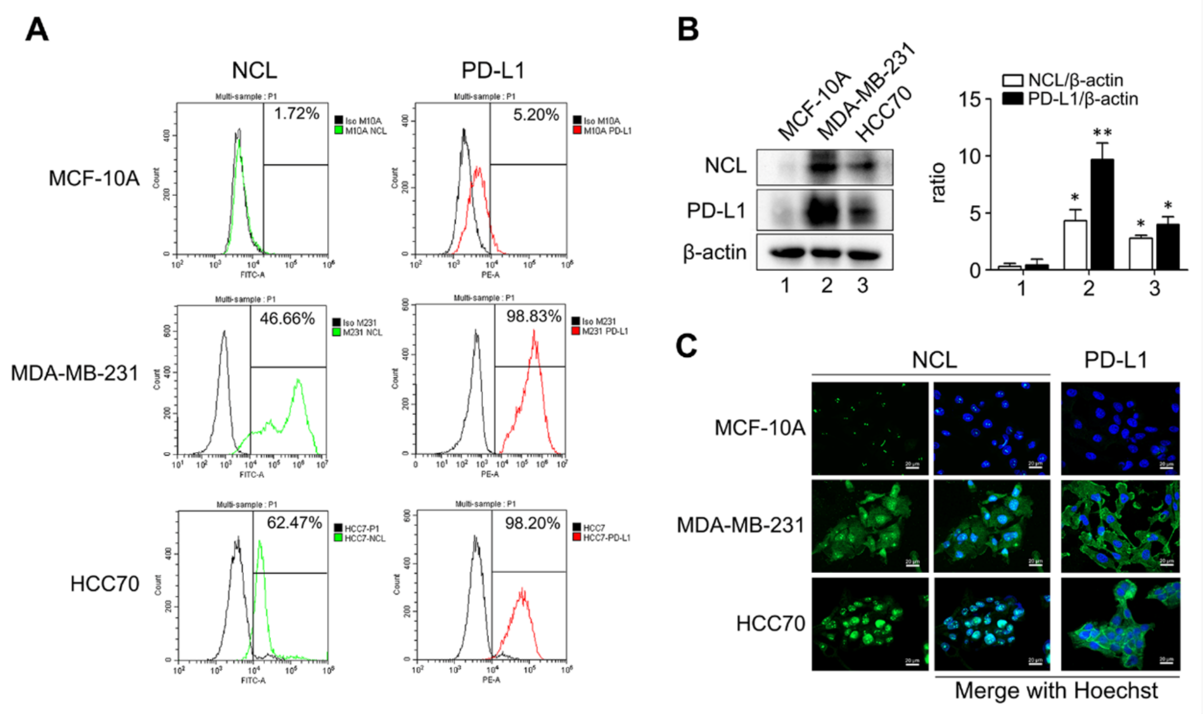

Supplementary Figure S4

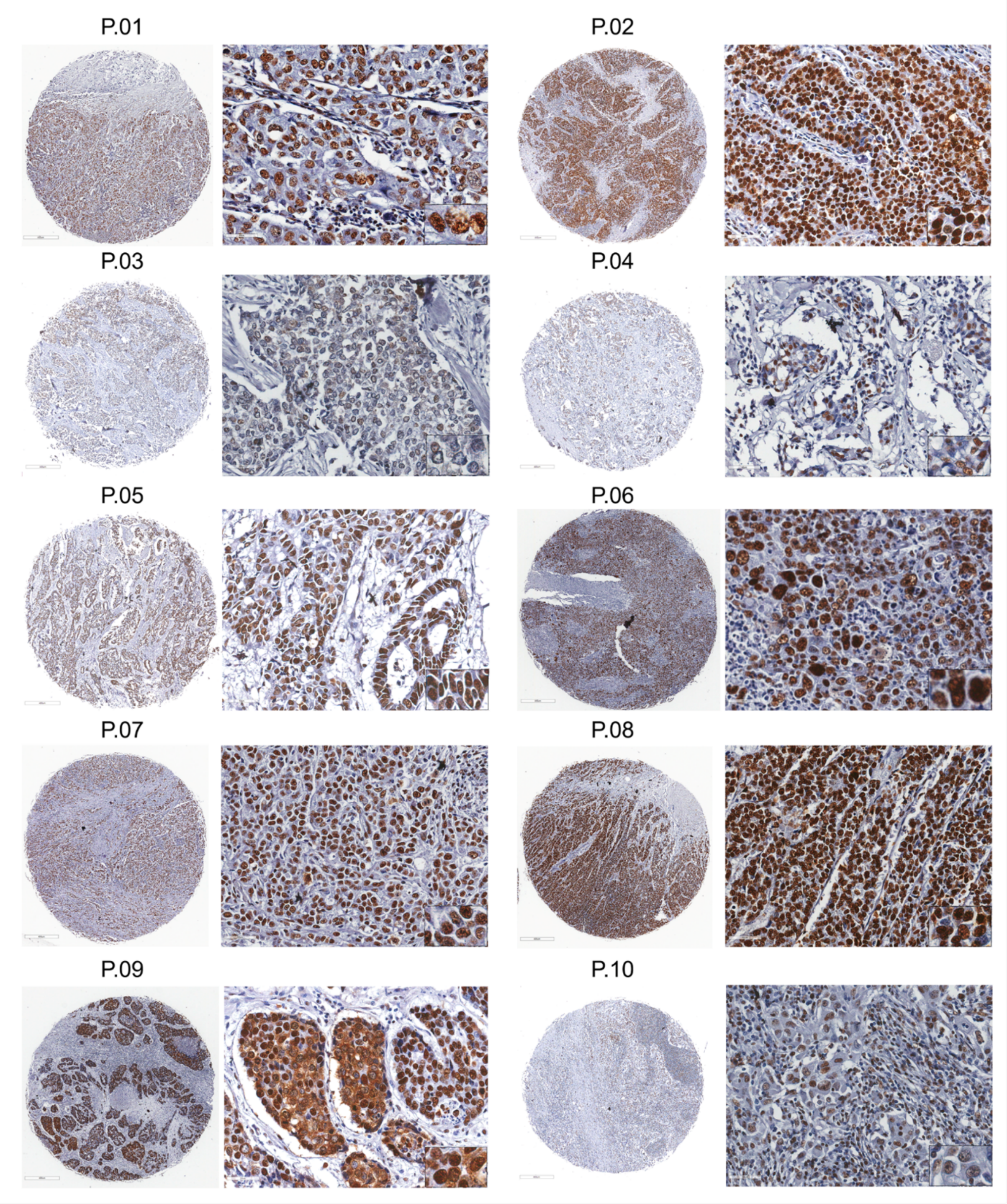

Supplementary Figure S5

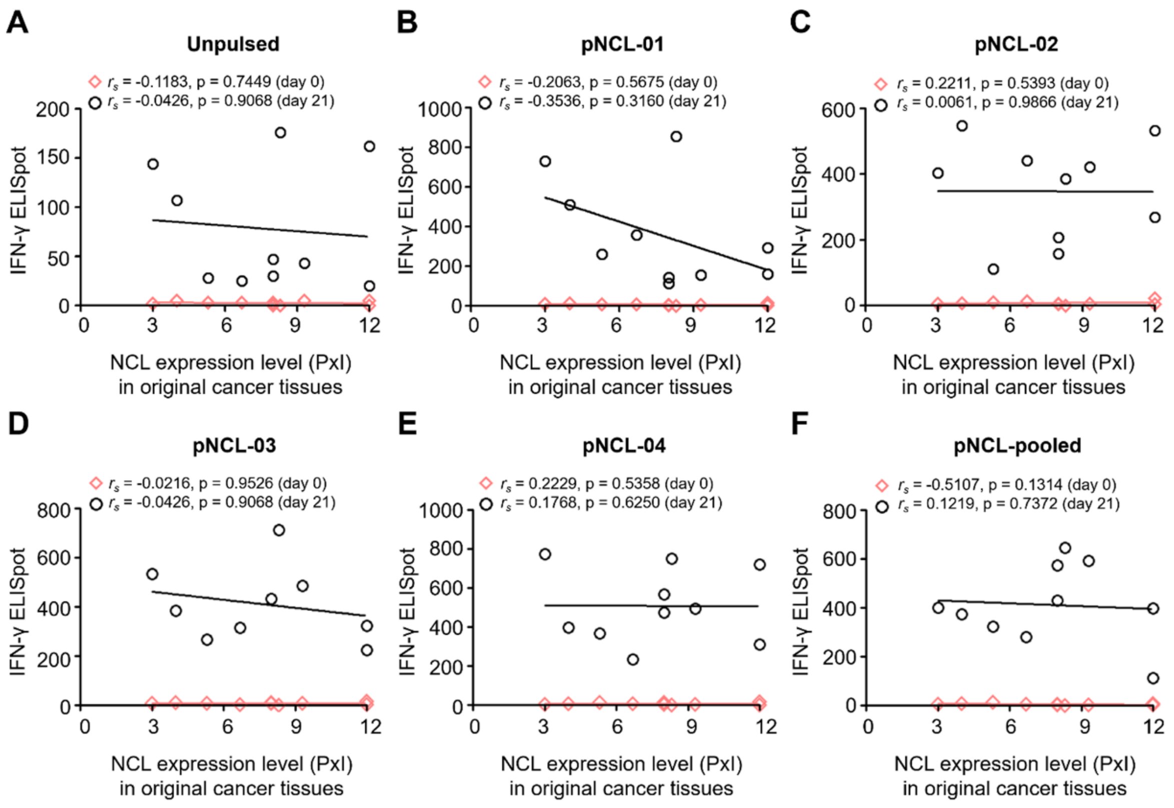

Supplementary Figure S6

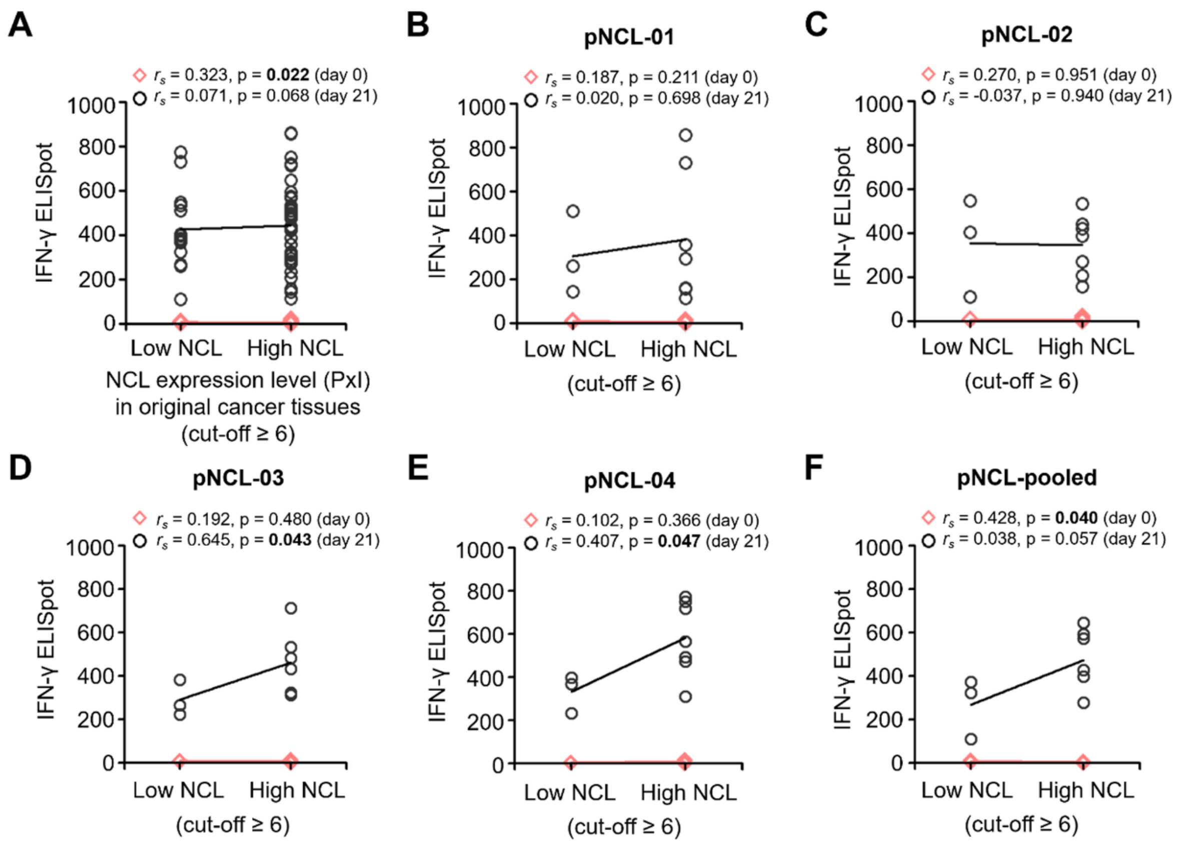

Supplementary Figure S7

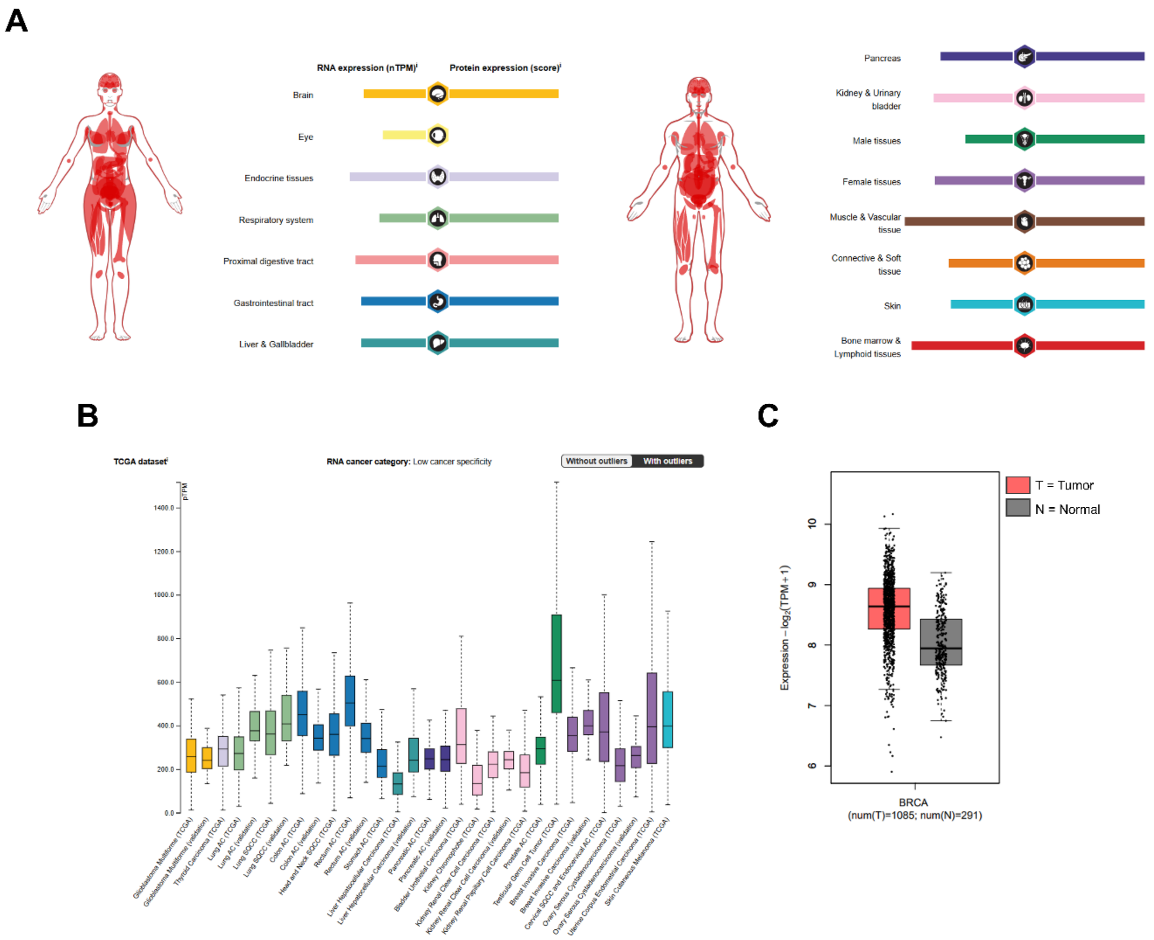

Supplementary Figure S8

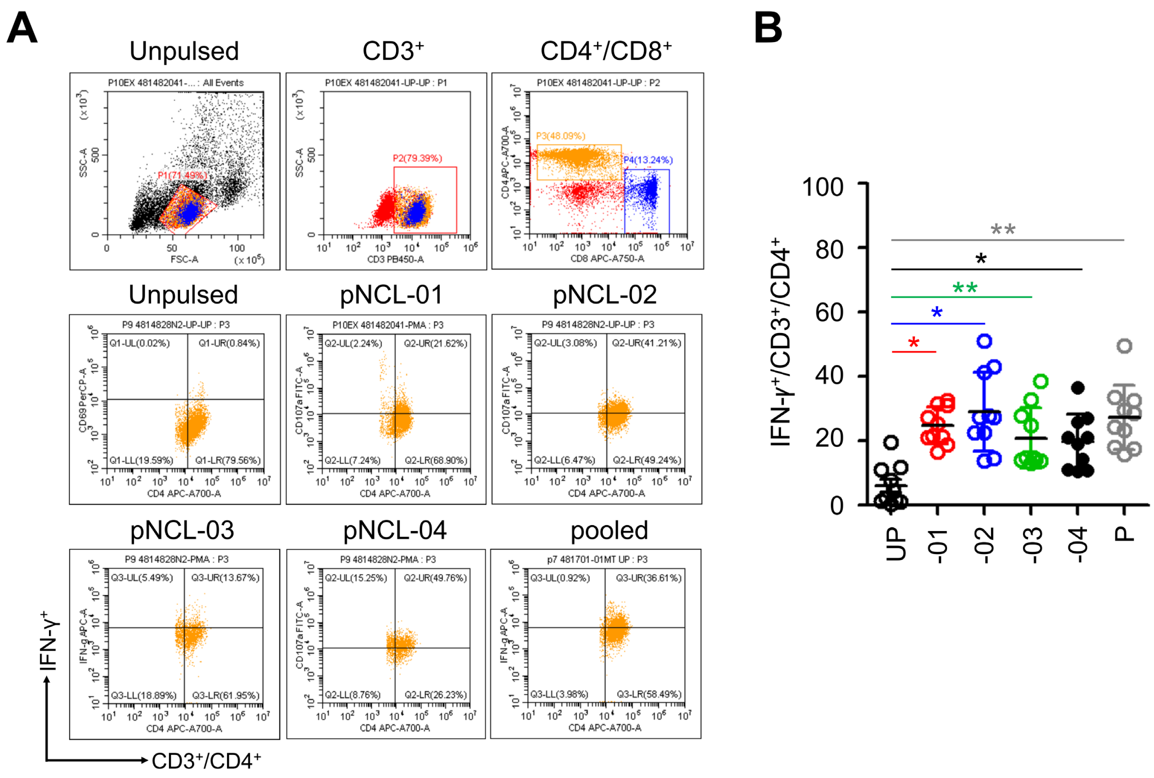

Supplementary Figure S9

Supplementary Table S1. HLA typing of breast cancer cells, healthy donors and patients.

Subjects	HLA typing					
	A*	A*	B*	B*	C*	C*
MCF-10A	01:01	33:01	55:01	40:01	07:02	03:03
MDA-MB-231	**02:17**	**02:17**	41:01	40:02	02:02	17:01
HCC70	30:02	03:01	78:01	**15:16**	16:01	16:01
Healthy donor 1	**02:03**	**02:06**	13:01	40:01	04:03	04:03
Healthy donor 2	**02:03**	33:03	46:01	58:01	01:02	03:02
Healthy donor 3	**02:07**	11:01	46:01	58:01	01:02	03:02
Healthy donor 4	**02:03**	11:01	**15:02**	35:01	04:01	08:01
Healthy donor 5	11:01	11:01	**15:01**	**15:01**	03:04	08:01
Healthy donor 6	11:01	11:01	13:01	**15:02**	03:04	08:01
Patient 01 (P.01)	03:01	11:01	07:02	46:01	01:02	07:02
Patient 02 (P.02)	11:01	30:01	13:01	13:02	03:04	06:02
Patient 03 (P.03)	24:10	30:01	13:02	18:02	06:02	07:04
Patient 04 (P.04)	**02:03**	**02:03**	38:02	38:02	07:02	07:02
Patient 05 (P.05)	**02:11**	24:02	40:06	51:01	04:01	15:02
Patient 06 (P.06)	**02:01**	24:02	35:01	39:06	03:04	07:02
Patient 07 (P.07)	11:01	24:02	**15:02**	**15:32**	08:01	12:03
Patient 08 (P.08)	**02:03**	**02:03**	46:01	46:01	01:02	01:02
Patient 09 (P.09)	**02:07**	24:02	46:01	46:01	01:02	01:02
Patient 10 (P.10)	11:01/02	11:02	13:01	27:10	04:06	12:02

Department of Transfusion Medicine, Faculty of Medicine Siriraj Hospital, Mahidol University; HLA of HCC70 was followed ttps://web.expasy.org/cellosaurus/CVCL_1270).

Bold represent HDs who have HLAs patrial match with MDA-MB-231 and HCC70 cells.

Supplementary Table S2. Summarize of candidate predicted NCL epitopes restricted to patients HLAs No. P.01-P.10.

Host	Peptide sequence	HLA	*in silico* prediction								MD simulation					
			NetMHC		NetMHCpan		NetMHCCons		NetCTLpan	Pick pocket	ΔG_{SIE}	MM/ GBSA	$\Delta G_{MM/GBSA}$	MM/ PBSA	delta MM/ PBSA	Distance (Å)
			Affinity (nM)	%Rank	Affinity (nM)	%Rank	Affinity (nM)	%Rank	%Rank	Affinity (nM)					$\Delta G_{MM/PBSA}$	(P2-P9)[a]
P.01	pNCL-01 KMAPPPKEV	B*46:01	10980.94	1.70 (WB)	21473.16	3.872 (WB)	N/A	N/A	9	0.251	-12.82	-64.144	-12.81	-62.579	-11.24	18.88
	pNCL-04 YAFIEFASF	B*46:01	107.5	0.01 (SB)	130.58	0.008 (SB)	196.38	0.03 (SB)	0.05 (<-E)	0.52	-12.47	-61.333	-3.37	-51.554	6.4	20.86
		C*01:02	N/A	N/A	4336.64	1.132 (SB)	2496.64	0.80 (WB)	0.10 (<-E)	0.287	N/D	N/D	N/D	N/D	N/D	N/D
		C*07:02	4322.16	1.40 (WB)	890.35	0.423 (SB)	N/A	N/A	0.10 (<-E)	0.084	N/D	N/D	N/D	N/D	N/D	N/D
P.02	pNCL-04 YAFIEFASF	B*13:01	N/A	N/A	1442.33	1.090 (WB)	1509.57	1.50 (WB)	0.40 (<-E)	0.334	-9.68	-43.799	-1.91	-42.984	-1.09	18.91
		C*03:04	N/A	N/A	6.98	0.029 (SB)	10.68	0.15 (SB)	0.15 (<-E)	0.397	N/D	N/D	N/D	N/D	N/D	N/D
		C*06:02	11392.17	4	4411.93	1.477 (WB)	N/A	N/A	0.05 (<-E)	0.255	N/D	N/D	N/D	N/D	N/D	N/D
P.03	pNCL-04 YAFIEFASF	A*24:10	N/A	N/A	114.74	0.585 (WB)	145.31	2.00 (SB)	0.80 (<-E)	0.325	-13.37	-56.239	-9.57	-60.373	-13.7	20.19
		B*18:02	N/A	N/A	437.33	0.240 (WB)	446.66	0.50 (SB)	0.30 (<-E)	0.424	N/D	N/D	N/D	N/D	N/D	N/D
		C*06:02	11392.17	4	4411.93	1.477 (WB)	N/A	N/A	0.05 (<-E)	0.255	N/D	N/D	N/D	N/D	N/D	N/D
		C*07:04	N/A	N/A	13673.02	2.704 (WB)	N/A	N/A	0.10 (<-E)	0.164	N/D	N/D	N/D	N/D	N/D	N/D
P.04	pNCL-01 KMAPPPKEV	A*02:03	17.68	0.50 (SB)	19.05	0.470 (SB)	18.97	1.50 (SB)	0.80 (<-E)	0.731	-10.08	-44.396	6.96	-23.952	27.4	20.27
	pNCL-02 VLSNLSYSA	A*02:03	26.21	0.70 (WB)	29.79	0.721 (WB)	14.48	1.00 (SB)	1	0.608	N/D	N/D	N/D	N/D	N/D	N/D
	pNCL-04 YAFIEFASF	C*07:02	4322.16	1.40 (WB)	890.35	0.423 (WB)	N/A	N/A	0.01 (<-E)	0.084	N/D	N/D	N/D	N/D	N/D	N/D
P.05	pNCL-01 KMAPPPKEV	A*02:11	5.37	0.25 (WB)	9.53	0.535 (SB)	5.8	1.50 (SB)	0.80 (<-E)	0.552	N/D	N/D	N/D	N/D	N/D	N/D
	pNCL-02 VLSNLSYSA	A*02:11	25.64	0.70 (WB)	9.22	0.517 (SB)	5.83	1.50 (SB)	1	0.588	N/D	N/D	N/D	N/D	N/D	N/D
	pNCL-04 YAFIEFASF	A*24:02	1367.83	1.40 (WB)	677.82	0.757 (WB)	N/A	N/A	0.80 (<-E)	0.321	N/D	N/D	N/D	N/D	N/D	N/D
		B*51:01	3063.3	0.60 (WB)	905.54	0.157 (SB)	4062.65	1.50 (WB)	0.80 (<-E)	0.381	-14.85	-68.546	-17.82	-62.202	-11.47	20.13
		C*15:02	4700.12	2.5	3020.42	2.307 (WB)	N/A	N/A	0.80 (<-E)	0.308	N/D	N/D	N/D	N/D	N/D	N/D

P.06	pNCL-01	A*02:01	236.91	1.80 (SB)	109.03	1.146 (SB)	264.44	4.00 (WB)	1.5	0.607	N/D	N/D	N/D	N/D	N/D	N/D
	KMAPPPKEV	C*03:03	874.82	1.30 (WB)	3837.39	2.982 (WB)	N/A	N/A	5	0.264	N/D	N/D	N/D	N/D	N/D	N/D
	pNCL-02<br>VLSNLSYSA	A*02:01	33.89	0.50 (SB)	65.24	0.794 (WB)	32.24	1.50 (SB)	1.5	0.632	-12.77	-61.808	-10.7	-54.639	-3.53	21.45
	pNCL-03<br>TLVLSNLSY	B*35:01	921.84	1.60 (WB)	236.52	0.461 (SB)	487.32	3.00 (WB)	1.5	0.315	N/D	N/D	N/D	N/D	N/D	N/D
	pNCL-04	A*24:02	1367.83	1.40 (WB)	677.82	0.757 (WB)	N/A	N/A	0.80 (<-E)	0.321	N/D	N/D	N/D	N/D	N/D	N/D
	YAFIEFASF	B*35:01	14.53	0.09 (SB)	6.38	0.020 (SB)	9.44	0.20 (SB)	0.10 (<-E)	0.549	N/D	N/D	N/D	N/D	N/D	N/D
		C*03:03	4.62	0.03 (SB)	6.98	0.029 (SB)	6.29	0.12 (SB)	0.15 (<-E)	0.397	N/D	N/D	N/D	N/D	N/D	N/D
		C*07:02	4322.16	1.40 (WB)	890.35	0.423 (WB)	N/A	N/A	0.01 (<-E)	0.084	N/D	N/D	N/D	N/D	N/D	N/D
P.07	pNCL-01<br>KMAPPPKEV	C*12:03	66.27	0.25 (SB)	654.4	1.248 (SB)	238.6	3.00 (WB)	10	0.99	N/D	N/D	N/D	N/D	N/D	N/D
	pNCL-03<br>TLVLSNLSY	B*15:02	313.79	0.17 (SB)	21.03	0.029 (SB)	65.13	0.17 (SB)	0.30 (<-E)	0.353	N/D	N/D	N/D	N/D	N/D	N/D
	pNCL-04	A*24:02	1367.83	1.40 (WB)	677.82	0.757 (WB)	N/A	N/A	0.80 (<-E)	0.31	N/D	N/D	N/D	N/D	N/D	N/D
	YAFIEFASF	B*15:02	565.34	0.30 (SB)	13.96	0.017 (SB)	197.45	0.80 (WB)	0.05 (<-E)	0.469	-15.77	-78.847	-20.48	-62.403	-4.04	17.79
		B*15:32	N/A	N/A	66.59	0.017 (SB)	51.92	0.10 (SB)	0.05 (<-E)	0.546	N/D	N/D	N/D	N/D	N/D	N/D
		C*08:01	N/A	N/A	582.09	0.076 (SB)	1914.61	1.50 (WB)	0.20 (<-E)	0.384	N/D	N/D	N/D	N/D	N/D	N/D
		C*12:03	12.5	0.03 (SB)	10.44	0.034 (SB)	11.6	0.10 (SB)	0.05 (<-E)	0.329	N/D	N/D	N/D	N/D	N/D	N/D
P.08	pNCL-01<br>KMAPPPKEV	A*02:03	17.68	0.50 (SB)	19.05	0.470 (SB)	18.97	1.50 (SB)	0.80 (<-E)	0.703						
	pNCL-02<br>VLSNLSYSA	A*02:03	26.21	0.70 (WB)	19.05	0.470 (SB)	14.48	1.00 (SB)	1	0.608	N/D	N/D	N/D	N/D	N/D	N/D
	pNCL-04	B*46:01	107.5	0.01 (SB)	130.58	0.008 (SB)	196.38	0.03 (SB)	0.05 (<-E)	0.52						
	YAFIEFASF	C*01:02	N/A	N/A	4336.64	1.132 (WB)	2496.64	0.80 (WB)	0.10 (<-E)	0.287	N/D	N/D	N/D	N/D	N/D	N/D
P.09	pNCL-01<br>KMAPPPKEV	B*46:01	10980.94	1.70 (WB)	21473.16	3.872 (WB)	N/A	N/A	9	0.251	N/D	N/D	N/D	N/D	N/D	N/D
	pNCL-04	A*24:02	1367.83	1.40 (WB)	677.82	0.757 (WB)	N/A	N/A	0.80 (<-E)	0.321	N/D	N/D	N/D	N/D	N/D	N/D
	YAFIEFASF	B*46:01	107.5	0.01 (SB)	130.58	0.008 (SB)	196.38	0.03 (SB)	0.05 (<-E)	0.52						
		C*01:02	N/A	N/A	4336.64	1.132 (WB)	2496.64	0.80 (WB)	0.10 (<-E)	0.287	N/D	N/D	N/D	N/D	N/D	N/D
P.10	pNCL-04	B*13:01	N/A	N/A	1442.33	1.090 (WB)	1509.57	1.50 (WB)	0.40 (<-E)	0.334	N/D	N/D	N/D	N/D	N/D	N/D

	YAFIEFASF	B*27:10	N/A	N/A	20604.62	7.967	N/A	N/A	3	0.201	N/D	N/D	N/D	N/D	N/D	N/D
		C*04:06	N/A	N/A	1072.27	0.185 (SB)	N/A	N/A	0.20 (<-E)	0.255	N/D	N/D	N/D	N/D	N/D	N/D
		C*12:02	N/A	N/A	11	0.009 (SB)	36.94	0.25 (SB)	0.01 (<-E)	0.478						

Underline: The binding score was obtained from the DTU Health Tech (http://www.cbs.dtu.dk/). N/A: not applicable; NetMHC: Threshold for strong binder (%Rank) 0.5; Threshold for weak binder (%Rank) 2; NetMHCPan: Threshold for strong binder (%Rank) 0.5; Threshold for weak binder (%Rank) 2.0; NetMHCcons: Threshold for strong binder (%Rank) 0.5; Threshold for strong binder IC50 < 500 nM; Threshold for weak binder (%Rank) 2.0; Threshold for weak binder IC50 < 2000 nM; NetCTLpan: Threshold for epitope identification (%Rank) 1.0; PickPocket: Prediction values IC50 < 0.500 nM are considered; SB: strong binding; WB: weak binding. Grey highlight boxes indicate candidate peptides that fulfilled at least three out of five selection criteria.

Supplementary Table S3. Characterization of *in silico* predicted NCL epitopes restricted to HLA of patient No. P.01.

Peptide sequence	Host	HLA	Peptide length	NetMHC		NetMHCpan		NetMHCcons		NetCTLpan	PickPocket
				Affinity (nM)	%Rank	Affinity (nM)	%Rank	Affinity (nM)	%Rank	%Rank	Affinity (nM)
pNCL-01	MDA-MB-231	A*02:01	9	236.91	0.50 (SB)	109.03	0.047 (SB)	264.44	4.00 (WB)	1.5	0.607
KMAPPPKEV		A*02:17	9	489.96	0.70 (WB)	2058.36	0.111 (SB)	1700.36	3	0.80 (<-E)	0.496
	HCC70	B*15:01	9	7850.23	12	5019	1.391 (WB)	8387.64	16	9	0.177
		B*15:16	9	N/A	N/A	10018.8	1.359 (WB)	4454	32	10	0.199
	P.01	A*03:01	9	12095.66	10	1464.41	2.163	N/A	N/A	7	0.251
		A*11:01	9	24221.97	21	26106.57	23.642	N/A	N/A	16	0.15
		B*07:02	9	7573.24	6	13319.8	8.5	N/A	N/A	7	0.129
		B*46:01	9	10980.94	1.70 (WB)	21473.16	3.872 (WB)	N/A	N/A	9	0.251
		C*01:02	9	N/A	N/A	3810.83	0.993 (SB)	N/A	N/A	5	0.135
		C*07:02	9	13025.21	4.5	8153.48	4.081 (WB)	N/A	N/A	16	0.201
pNCL-02	MDA-MB-231	A*02:01	9	33.89	0.50 (SB)	65.24	0.822 (WB)	32.24	1.50 (SB)	1.5	0.632
VLSNLSYSA		A*02:17	9	14881.86	8.5	4084.42	1.823 (WB)	3954.23	5	3	0.492
	HCC70	B*15:01	9	3113.83	6.5	3921.46	6.396	N/A	N/A	8	0.183
		B*15:16	9	N/A	N/A	23716.53	2.776	21154.46	50	32	0.065
	P.01	A*03:01	9	17072.29	15	12735.76	9.302	N/A	N/A	7	0.201
		A*11:01	9	12281.48	10	17325.74	13.321	N/A	N/A	16	0.221
		B*07:02	9	22588.48	18	18769.79	13.2	N/A	N/A	32	0.182
		B*46:01	9	22787.08	9	31136.69	10.734	N/A	N/A	16	0.104
		C*01:02	9	34385.58	23	20971.68	8.575	N/A	N/A	32	0.019
		C*07:02	9	N/A	N/A	18089.7	11.972	N/A	N/A	32	0.065
pNCL-03	MDA-MB-231	A*02:01	9	12252.01	18	18209.68	11.633	21040.32	32	9	0.178
TLVLSNLSY		A*02:17	9	19774.85	12	25913.51	8.828	24481.5	32	8	0.179
	HCC70	B*15:01	9	65.7	0.60 (WB)	42.63	0.111 (SB)	80.87	1.00 (WB)	0.80 (<-E)	0.378
		B*15:16	9	N/A	N/A	6714.67	2.224	2751.98	15	4	0.244
	P.01	A*03:01	9	1163.23	2.5	1464.41	2.163	N/A	N/A	1	0.298
		A*11:01	9	1241.77	3	1510.81	2.866	N/A	N/A	3	0.246
		B*07:02	9	23023.75	19	28136.6	27.061	N/A	N/A	7	0.056
		B*46:01	9	17538.3	4.5	8822.92	0.735 (SB)	N/A	N/A	1.5	0.213

		C*01:02	9	N/A	N/A	30132.46	17.613	N/A	N/A	0.80 (<-E)	0.029
		C*07:02	9	25855.82	13	8946.06	4.553	N/A	N/A	9	0.073
pNCL-04	MDA-MB-231	A*02:01	9	5774.27	11	11147.34	14.589	11479.16	32	7	0.232
YAFIEFASF		A*02:17	9	35560.78	28	9786.84	3.892	16316.49	15	3	0.091
	HCC70	B*15:01	9	20.24	0.17 (SB)	25.05	0.460 (SB)	20.03	0.10 (SB)	0.15 (<-E)	0.546
		B*15:16	9	N/A	N/A	103.55	0.156 (SB)	105.42	1.00 (WB)	0.40 (<-E)	0.458
	P.01	A*03:01	9	23031.47	23	22592.39	19.721	N/A	N/A	8	0.205
		A*11:01	9	18826.94	25	20123.87	15.908	N/A	N/A	9	0.247
		B*07:02	9	14224.56	10	7704.32	5.001	N/A	N/A	2	0.317
		B*46:01	9	107.5	0.01 (SB)	130.58	0.008 (SB)	196.38	0.03 (SB)	0.05 (<-E)	0.52
		C*01:02	9	N/A	N/A	4336.64	1.132 (SB)	2496.64	0.80 (WB)	0.10 (<-E)	0.287
		C*07:02	9	4322.16	1.40 (WB)	890.35	0.423 (SB)	N/A	N/A	0.10 (<-E)	0.084

Underline: The binding score was obtained from the DTU Health Tech (http://www.cbs.dtu.dk/). N/A: not applicable; NetMHC: Threshold for strong binder (%Rank) 0.5; Threshold for weak binder (%Rank) 2; NetMHCPan: Threshold for strong binder (%Rank) 0.5; Threshold for weak binder (%Rank) 2.0; NetMHCcons: Threshold for strong binder (%Rank) 0.5; Threshold for strong binder IC50 < 500 nM; Threshold for weak binder (%Rank) 2.0; Threshold for weak binder IC50 < 2000 nM; NetCTLpan: Threshold for epitope identification (%Rank) 1.0; PickPocket: Prediction values IC50 < 0.500 nM are considered; SB: strong binding; WB: weak binding. Grey highlight boxes indicate candidate peptides that fulfilled at least three out of five selection criteria.

Supplementary Table S4. Characterization of *in silico* predicted NCL epitopes restricted to HLA of patient No. P.02.

Peptide sequence	Host	HLA	Peptide length	NetMHC		NetMHCpan		NetMHCcons		NetCTLpan	PickPocket
				Affinity (nM)	%Rank	Affinity (nM)	%Rank	Affinity (nM)	%Rank	%Rank	Affinity (nM)
pNCL-01	MDA-MB-231	A*02:01	9	236.91	1.80 (WB)	109.03	0.047 (SB)	264.44	4.00 (WB)	1.5	0.607
KMAPPPKEV		A*02:17	9	489.96	0.70 (WB)	2058.36	0.111 (SB)	1700.36	3	0.80 (<-E)	0.496
	HCC70	B*15:01	9	7850.23	12	5019	1.391 (WB)	8387.64	16	9	0.177
		B*15:16	9	N/A	N/A	10018.8	1.359 (WB)	4454	32	10	0.199
	P.02	A*11:01	9	24221.97	21	26106.57	23.642	N/A	N/A	16	0.15
		A*30:01	9	3330.88	5.5	1283.28	4.347 (SB)	N/A	N/A	4	0.244
		B*13:01	9	N/A	N/A	4029.38	3.479 (SB)	N/A	N/A	16	0.173
		B*13:02	9	N/A	N/A	3320.52	0.673 (SB)	N/A	N/A	16	0.235
		C*03:04	9	N/A	N/A	3837.39	2.982 (WB)	N/A	N/A	5	0.264
		C*06:02	9	2118.59	9	2796.92	0.905 (SB)	N/A	N/A	16	0.165
pNCL-02	MDA-MB-231	A*02:01	9	33.89	0.50 (SB)	65.24	0.822 (WB)	32.24	1.50 (SB)	1.5	0.632
VLSNLSYSA		A*02:17	9	14881.86	8.5	4084.42	1.823 (WB)	3954.23	5	3	0.492
	HCC70	B*15:01	9	3113.83	6.5	3921.46	6.396	N/A	N/A	8	0.183
		B*15:16	9	N/A	N/A	23716.53	2.776	21154.46	50	32	0.065
	P.02	A*11:01	9	12281.48	10	17325.74	13.321	N/A	N/A	16	0.221
		A*30:01	9	6556.65	9.5	5251.04	13.157	N/A	N/A	32	0.18
		B*13:01	9	N/A	N/A	5793.42	5.292	N/A	N/A	32	0.145
		B*13:02	9	N/A	N/A	7518.05	2.128	N/A	N/A	32	0.146
		C*03:04	9	N/A	N/A	21540.17	13.53	N/A	N/A	16	0.191
		C*06:02	9	37826.63	30	23411.87	14.656	N/A	N/A	32	0.066
pNCL-03	MDA-MB-231	A*02:01	9	12252.01	18	18209.68	11.633	21040.32	32	9	0.178
TLVLSNLSY		A*02:17	9	19774.85	12	25913.51	8.828	24481.5	32	8	0.179
	HCC70	B*15:01	9	65.7	0.60 (WB)	42.63	0.111 (SB)	80.87	1.00 (WB)	0.80 (<-E)	0.378
		B*15:16	9	N/A	N/A	6714.67	2.224	2751.98	15	4	0.244
	P.02	A*11:01	9	1241.77	3	1510.81	2.866	N/A	N/A	3	0.246
		A*30:01	9	11844.03	17	15202.31	35.408	N/A	N/A	16	0.144
		B*13:01	9	N/A	N/A	10794.81	10.888	N/A	N/A	4	0.196

		B*13:02	9	N/A	N/A	29187.15	25.105	N/A	N/A	5	0.09
		C*03:04	9	N/A	N/A	15059.87	9.03	N/A	N/A	9	0.061
		C*06:02	9	35250.48	24	26739.8	19.437	N/A	N/A	3	0.047
pNCL-04	MDA-MB-231	A*02:01	9	5774.27	11	11147.34	14.589	11479.16	32	7	0.232
YAFIEFASF		A*02:17	9	35560.78	28	9786.84	3.892	16316.49	15	3	0.091
	HCC70	B*15:01	9	20.24	0.17 (SB)	25.05	0.460 (SB)	20.03	0.10 (SB)	0.15 (<-E)	0.546
		B*15:16	9	N/A	N/A	103.55	0.156 (SB)	105.42	1.00 (WB)	0.40 (<-E)	0.458
	P.02	A*11:01	9	18826.94	15	20123.87	15.908	N/A	N/A	9	0.247
		A*30:01	9	9827.9	14	12921.47	29.936	N/A	N/A	16	0.165
		B*13:01	9	N/A	N/A	1442.33	1.090 (WB)	1509.57	1.50 (WB)	0.40 (<-E)	0.334
		B*13:02	9	N/A	N/A	16349	7.52	N/A	N/A	0.80 (<-E)	0.272
		C*03:04	9	N/A	N/A	6.98	0.029 (SB)	10.68	0.15 (SB)	0.15 (<-E)	0.397
		C*06:02	9	11392.17	4	4411.93	1.477 (WB)	N/A	N/A	0.05 (<-E)	0.255

Underline: The binding score was obtained from the DTU Health Tech (http://www.cbs.dtu.dk/). N/A: not applicable; NetMHC: Threshold for strong binder (%Rank) 0.5; Threshold for weak binder (%Rank) 2; NetMHCPan: Threshold for strong binder (%Rank) 0.5; Threshold for weak binder (%Rank) 2.0; NetMHCcons: Threshold for strong binder (%Rank) 0.5; Threshold for strong binder IC50 < 500 nM; Threshold for weak binder (%Rank) 2.0; Threshold for weak binder IC50 < 2000 nM; NetCTLpan: Threshold for epitope identification (%Rank) 1.0; PickPocket: Prediction values IC50 < 0.500 nM are considered; SB: strong binding; WB: weak binding. Grey highlight boxes indicate candidate peptides that fulfilled at least three out of five selection criteria.

Supplementary Table S5. Characterization of *in silico* predicted NCL epitopes restricted to HLA of patient No. P.03.

Peptide sequence	Host	HLA	Peptide length	NetMHC		NetMHCpan		NetMHCcons		NetCTLpan	PickPocket
				Affinity (nM)	%Rank	Affinity (nM)	%Rank	Affinity (nM)	%Rank	%Rank	Affinity (nM)
pNCL-01	MDA-MB-231	A*02:01	9	236.91	1.80 (WB)	109.03	0.047 (SB)	264.44	4.00 (WB)	1.5	0.607
KMAPPPKEV		A*02:17	9	489.96	0.70 (WB)	2058.36	0.111 (SB)	1700.36	3	0.80 (<-E)	0.496
	HCC70	B*15:01	9	7850.23	12	5019	1.391 (WB)	8387.64	16	9	0.177
		B*15:16	9	N/A	N/A	10018.8	1.359 (WB)	4454	32	10	0.199
	P.03	A*24:10	9	N/A	N/A	11956.17	11.725	N/A	N/A	16	0.133
		A*30:01	9	3330.88	5.5	1283.28	4.347 (SB)	N/A	N/A	4	0.244
		B*13:02	9	N/A	N/A	3320.52	0.673 (SB)	N/A	N/A	16	0.235
		B*18:02	9	N/A	N/A	36601.09	69.495	N/A	N/A	32	0.038
		C*06:02	9	21188.59	9	2796.92	0.905 (SB)	N/A	N/A	16	0.165
		C*07:04	9	N/A	N/A	5787.53	0.407 (SB)	N/A	N/A	16	0.253
pNCL-02	MDA-MB-231	A*02:01	9	33.89	0.50 (SB)	65.24	0.822 (WB)	32.24	1.50 (SB)	1.5	0.632
VLSNLSYSA		A*02:17	9	14881.86	8.5	4084.42	1.823 (WB)	3954.23	5	3	0.492
	HCC70	B*15:01	9	3113.83	6.5	3921.46	6.396	N/A	N/A	8	0.183
		B*15:16	9	N/A	N/A	23716.53	2.776	21154.46	50	32	0.065
	P.03	A*24:10	9	N/A	N/A	21971.64	22.652	N/A	N/A	32	0.158
		A*30:01	9	6556.65	9.5	5251.04	13.157	N/A	N/A	32	0.18
		B*13:02	9	N/A	N/A	7518.05	2.128	N/A	N/A	32	0.146
		B*18:02	9	N/A	N/A	17568.88	14.377	N/A	N/A	32	0.141
		C*06:02	9	37826.63	30	23411.87	14.656	N/A	N/A	32	0.066
		C*07:04	9	N/A	N/A	18367.99	5.387	N/A	N/A	32	0.072
pNCL-03	MDA-MB-231	A*02:01	9	12252.01	18	18209.68	11.633	21040.32	32	9	0.178
TLVLSNLSY		A*02:17	9	19774.85	12	25913.51	8.828	24481.5	32	8	0.179
	HCC70	B*15:01	9	65.7	0.60 (WB)	42.63	0.111 (SB)	80.87	1.00 (WB)	0.80 (<-E)	0.378
		B*15:16	9	N/A	N/A	6714.67	2.224	2751.98	15	4	0.244
	P.03	A*24:10	9	N/A	N/A	19581.31	19.643	N/A	N/A	16	0.06
		A*30:01	9	11844.03	17	15202.31	35.408	N/A	N/A	5	0.144

		B*13:02	9	N/A	N/A	29187.15	25.105	N/A	N/A	3	0.09
		B*18:02	9	N/A	N/A	6564.53	3.75	N/A	N/A	3	0.345
		C*06:02	9	35250.48	24	26739.8	19.437	N/A	N/A	16	0.047
		C*07:04	9	N/A	N/A	34134.25	28.922	N/A	N/A	16	0.018
pNCL-04	MDA-MB-231	A*02:01	9	5774.27	11	11147.34	14.589	11479.16	32	7	0.232
YAFIEFASF		A*02:17	9	35560.78	28	9786.84	3.892	16316.49	15	3	0.091
	HCC70	B*15:01	9	20.24	0.17 (SB)	25.05	0.460 (SB)	20.03	0.10 (SB)	0.15 (<-E)	0.546
		B*15:16	9	N/A	N/A	103.55	0.156 (SB)	105.42	1.00 (WB)	0.40 (<-E)	0.458
	P.03	A*24:10	9	N/A	N/A	114.74	0.585 (WB)	145.31	2.00 (SB)	0.80 (<-E)	0.325
		A*30:01	9	9827.9	14	12921.47	29.936	N/A	N/A	16	0.156
		B*13:02	9	N/A	N/A	16349	7.52	N/A	N/A	0.80 (<-E)	0.272
		B*18:02	9	N/A	N/A	437.33	0.240 (WB)	446.66	0.50 (SB)	0.30 (<-E)	0.424
		C*06:02	9	11392.17	4	4411.93	1.477 (WB)	N/A	N/A	0.05 (<-E)	0.255
		C*07:04	9	N/A	N/A	13673.02	2.704 (WB)	N/A	N/A	0.10 (<-E)	0.164

Underline: The binding score was obtained from the DTU Health Tech (http://www.cbs.dtu.dk/). N/A: not applicable; NetMHC: Threshold for strong binder (%Rank) 0.5; Threshold for weak binder (%Rank) 2; NetMHCPan: Threshold for strong binder (%Rank) 0.5; Threshold for weak binder (%Rank) 2.0; NetMHCcons: Threshold for strong binder (%Rank) 0.5; Threshold for strong binder IC50 < 500 nM; Threshold for weak binder (%Rank) 2.0; Threshold for weak binder IC50 < 2000 nM; NetCTLpan: Threshold for epitope identification (%Rank) 1.0; PickPocket: Prediction values IC50 < 0.500 nM are considered; SB: strong binding; WB: weak binding. Grey highlight boxes indicate candidate peptides that fulfilled at least three out of five selection criteria.

Supplementary Table S6. Characterization of *in silico* predicted NCL epitopes restricted to HLA of patient No. P.04.

Peptide sequence	Host	HLA	Peptide length	NetMHC		NetMHCpan		NetMHCcons		NetCTLpan	PickPocket
				Affinity (nM)	%Rank	Affinity (nM)	%Rank	Affinity (nM)	%Rank	%Rank	Affinity (nM)
pNCL-01	MDA-MB-231	A*02:01	9	236.91	1.80 (WB)	109.03	0.047 (SB)	264.44	4.00 (WB)	1.5	0.607
KMAPPPKEV		A*02:17	9	489.96	0.70 (WB)	2058.36	0.111 (SB)	1700.36	3	0.80 (<-E)	0.496
	HCC70	B*15:01	9	7850.23	12	5019	1.391 (WB)	8387.64	16	9	0.177
		B*15:16	9	N/A	N/A	10018.8	1.359 (WB)	4454	32	10	0.199
	P.04	A*02:03	9	17.68	0.50 (SB)	19.05	0.470 (SB)	18.97	1.50 (SB)	0.80 (<-E)	0.731
		B*38:02	9	N/A	N/A	29761.79	18.319	N/A	N/A	16	0.071
		C*07:02	9	13025.21	4.5	8153.48	4.081 (WB)	N/A	N/A	16	0.201
pNCL-02	MDA-MB-231	A*02:01	9	33.89	0.50 (SB)	65.24	0.822 (WB)	32.24	1.50 (SB)	1.5	0.632
VLSNLSYSA		A*02:17	9	14881.86	8.5	4084.42	1.823 (WB)	3954.23	5	3	0.492
	HCC70	B*15:01	9	3113.83	6.5	3921.46	6.396	N/A	N/A	8	0.183
		B*15:16	9	N/A	N/A	23716.53	2.776	21154.46	50	32	0.065
	P.04	A*02:03	9	26.21	0.70 (WB)	29.79	0.721 (WB)	14.48	1.00 (SB)	1	0.608
		B*38:02	9	N/A	N/A	29020.25	17.107	N/A	N/A	32	0.127
		C*07:02	9	34385.58	23	18089.7	11.972	N/A	N/A	32	0.065
pNCL-03	MDA-MB-231	A*02:01	9	12252.01	18	18209.68	11.633	21040.32	32	9	0.178
TLVLSNLSY		A*02:17	9	19774.85	12	25913.51	8.828	24481.5	32	8	0.179
	HCC70	B*15:01	9	65.7	0.60 (WB)	42.63	0.111 (SB)	80.87	1.00 (WB)	0.80 (<-E)	0.378
		B*15:16	9	N/A	N/A	6714.67	2.224	2751.98	15	4	0.244
	P.04	A*02:03	9	10054.96	21	17257.27	31.533	N/A	N/A	16	0.195
		B*38:02	9	N/A	N/A	35754.45	31.824	N/A	N/A	5	0.125
		C*07:02	9	25855.82	13	8946.06	4.553	N/A	N/A	9	0.073
pNCL-04	MDA-MB-231	A*02:01	9	5774.27	11	11147.34	14.589	11479.16	32	7	0.232
YAFIEFASF		A*02:17	9	35560.78	28	9786.84	3.892	16316.49	15	3	0.091
	HCC70	B*15:01	9	20.24	0.17 (SB)	25.05	0.460 (SB)	20.03	0.10 (SB)	0.15 (<-E)	0.546
		B*15:16	9	N/A	N/A	103.55	0.156 (SB)	105.42	1.00 (WB)	0.40 (<-E)	0.458
	P.04	A*02:03	9	6759.13	16	10183.83	21.532	N/A	N/A	8	0.194
		B*38:02	9	N/A	N/A	16134.95	4.839	N/A	N/A	0.80 (<-E)	0.289

		C*07:02	9	4322.16	1.40 (WB)	890.35	0.423 (WB)	N/A	N/A	0.01 (<-E)	0.084

Underline: The binding score was obtained from the DTU Health Tech (http://www.cbs.dtu.dk/). N/A: not applicable; NetMHC: Threshold for strong binder (%Rank) 0.5; Threshold for weak binder (%Rank) 2; NetMHCPan: Threshold for strong binder (%Rank) 0.5; Threshold for weak binder (%Rank) 2.0; NetMHCcons: Threshold for strong binder (%Rank) 0.5; Threshold for strong binder IC50 < 500 nM; Threshold for weak binder (%Rank) 2.0; Threshold for weak binder IC50 < 2000 nM; NetCTLpan: Threshold for epitope identification (%Rank) 1.0; PickPocket: Prediction values IC50 < 0.500 nM are considered; SB: strong binding; WB: weak binding. Grey highlight boxes indicate candidate peptides that fulfilled at least three out of five selection criteria.

Supplementary Table S7. Characterization of *in silico* predicted NCL epitopes restricted to HLA of patient No. P.05.

Peptide sequence	Host	HLA	Peptide length	NetMHC		NetMHCpan		NetMHCcons		NetCTLpan	PickPocket
				Affinity (nM)	%Rank	Affinity (nM)	%Rank	Affinity (nM)	%Rank	%Rank	Affinity (nM)
pNCL-01	MDA-MB-231	A*02:01	9	236.91	1.80 (WB)	109.03	0.047 (SB)	264.44	4.00 (WB)	1.5	0.607
KMAPPPKEV		A*02:17	9	489.96	0.70 (WB)	2058.36	0.111 (SB)	1700.36	3	0.80 (<-E)	0.496
	HCC70	B*15:01	9	7850.23	12	5019	1.391 (WB)	8387.64	16	9	0.177
		B*15:16	9	N/A	N/A	10018.8	1.359 (WB)	4454	32	10	0.199
	P.05	A*02:11	9	5.37	0.25 (WB)	9.53	0.535 (SB)	5.8	1.50 (SB)	0.80 (<-E)	0.552
		A*24:02	9	24141.9	16	22743.47	13.418	N/A	N/A	16	0.155
		B*40:06	9	N/A	N/A	30759	20.522	N/A	N/A	16	0.17
		B*51:01	9	25284.81	15	28460.53	15.44	N/A	N/A	16	0.062
		C*04:01	9	21266.67	13	11418.58	1.247 (SB)	N/A	N/A	16	0.212
		C*15:02	9	313.06	0.30 (SB)	723.68	0.646 (SB)	492.62	3.00 (WB)	8	0.265
pNCL-02	MDA-MB-231	A*02:01	9	33.89	0.50 (SB)	65.24	0.822 (WB)	32.24	1.50 (SB)	1.5	0.632
VLSNLSYSA		A*02:17	9	14881.86	8.5	4084.42	1.823 (WB)	3954.23	5	3	0.492
	HCC70	B*15:01	9	3113.83	6.5	3921.46	6.396	N/A	N/A	8	0.183
		B*15:16	9	N/A	N/A	23716.53	2.776	21154.46	50	32	0.065
	P.05	A*02:11	9	25.64	0.70 (WB)	9.22	0.517 (SB)	5.83	1.50 (SB)	1	0.588
		A*24:02	9	24272.87	17	25514.28	16.252	N/A	N/A	32	0.18
		B*40:06	9	N/A	N/A	20352.04	8.253	N/A	N/A	32	0.2
		B*51:01	9	31387.68	32	32937.7	23.599	N/A	N/A	32	0.051
		C*04:01	9	19541.52	11	23816.31	10.239	N/A	N/A	32	0.137
		C*15:02	9	33070.18	23	17431.04	12.987	N/A	N/A	32	0.135
pNCL-03	MDA-MB-231	A*02:01	9	12252.01	18	18209.68	11.633	21040.32	32	9	0.178
TLVLSNLSY		A*02:17	9	19774.85	12	25913.51	8.828	24481.5	32	8	0.179
	HCC70	B*15:01	9	65.7	0.60 (WB)	42.63	0.111 (SB)	80.87	1.00 (WB)	0.80 (<-E)	0.378
		B*15:16	9	N/A	N/A	6714.67	2.224	2751.98	15	4	0.244
	P.05	A*02:11	9	35602.75	30	16350.24	28.467	N/A	N/A	16	0.183
		A*24:02	9	37119.94	38	31119.19	24.175	N/A	N/A	8	0.068
		B*40:06	9	N/A	N/A	37417.54	40.971	N/A	N/A	8	0.051
		B*51:01	9	29674.97	26	36419.38	32.919	N/A	N/A	4	0.069

Peptide sequence	Host	HLA	Peptide length	NetMHC		NetMHCpan		NetMHCcons		NetCTLpan	PickPocket
				Affinity (nM)	%Rank	Affinity (nM)	%Rank	Affinity (nM)	%Rank	%Rank	Affinity (nM)
		C*04:01	9	17861.18	9	26436.3	14.679	N/A	N/A	10	0.041
		C*15:02	9	40199.43	40	25440.67	22.376	N/A	N/A	9	0.062
pNCL-04	MDA-MB-231	A*02:01	9	5774.27	11	11147.34	14.589	11479.16	32	7	0.232
YAFIEFASF		A*02:17	9	35560.78	28	9786.84	3.892	16316.49	15	3	0.091
	HCC70	B*15:01	9	20.24	0.17 (SB)	25.05	0.460 (SB)	20.03	0.10 (SB)	0.15 (<-E)	0.546
		B*15:16	9	N/A	N/A	103.55	0.156 (SB)	105.42	1.00 (WB)	0.40 (<-E)	0.458
	P.05	A*02:11	9	19518.28	14	6810.15	16.546	N/A	N/A	10	0.199
		A*24:02	9	1367.83	1.40 (WB)	677.82	0.757 (WB)	N/A	N/A	0.80 (<-E)	0.321
		B*40:06	9	N/A	N/A	15001.65	5.388	N/A	N/A	4	0.265
		B*51:01	9	3063.3	0.60 (WB)	905.54	0.157 (SB)	4062.65	1.50 (WB)	0.80 (<-E)	0.381
		C*04:01	9	10067.16	2.5	12809.42	1.636	N/A	N/A	0.20 (<-E)	0.122
		C*15:02	9	4700.12	2.5	3020.42	2.307 (WB)	N/A	N/A	0.80 (<-E)	0.308

Underline: The binding score was obtained from the DTU Health Tech (http://www.cbs.dtu.dk/). N/A: not applicable; NetMHC: Threshold for strong binder (%Rank) 0.5; Threshold for weak binder (%Rank) 2; NetMHCPan: Threshold for strong binder (%Rank) 0.5; Threshold for weak binder (%Rank) 2.0; NetMHCcons: Threshold for strong binder (%Rank) 0.5; Threshold for strong binder IC50 < 500 nM; Threshold for weak binder (%Rank) 2.0; Threshold for weak binder IC50 < 2000 nM; NetCTLpan: Threshold for epitope identification (%Rank) 1.0; PickPocket: Prediction values IC50 < 0.500 nM are considered; SB: strong binding; WB: weak binding. Grey highlight boxes indicate candidate peptides that fulfilled at least three out of five selection criteria.

Supplementary Table S8. Characterization of *in silico* predicted NCL epitopes restricted to HLA of patient No. P.06.

Peptide sequence	Host	HLA	Peptide length	NetMHC		NetMHCpan		NetMHCcons		NetCTLpan	PickPocket
				Affinity (nM)	%Rank	Affinity (nM)	%Rank	Affinity (nM)	%Rank	%Rank	Affinity (nM)
pNCL-01	MDA-MB-231	A*02:01	9	236.91	1.80 (WB)	109.03	0.047 (SB)	264.44	4.00 (WB)	1.5	0.607
KMAPPPKEV		A*02:17	9	489.96	0.70 (WB)	2058.36	0.111 (SB)	1700.36	3	0.80 (<-E)	0.496
	HCC70	B*15:01	9	7850.23	12	5019	1.391 (WB)	8387.64	16	9	0.177
		B*15:16	9	N/A	N/A	10018.8	1.359 (WB)	4454	32	10	0.199
	P.06	A*02:01	9	236.91	1.80 (SB)	109.03	1.146 (SB)	264.44	4.00 (WB)	1.5	0.607
		A*24:02	9	24141.9	16	22743.47	13.418	N/A	N/A	16	0.155
		B*35:01	9	30884.05	35	35353.61	38.223	N/A	N/A	32	0
		B*39:06	9	N/A	N/A	26570.22	20.711	N/A	N/A	16	0.128
		C*03:03	9	874.82	1.30 (WB)	3837.39	2.982 (WB)	N/A	N/A	5	0.264
		C*07:02	9	13025.21	4.5	8153.48	4.081 (WB)	N/A	N/A	16	0.201
pNCL-02	MDA-MB-231	A*02:01	9	33.89	0.50 (SB)	65.24	0.822 (WB)	32.24	1.50 (SB)	1.5	0.632
VLSNLSYSA		A*02:17	9	14881.86	8.5	4084.42	1.823 (WB)	3954.23	5	3	0.492
	HCC70	B*15:01	9	3113.83	6.5	3921.46	6.396	N/A	N/A	8	0.183
		B*15:16	9	N/A	N/A	23716.53	2.776	21154.46	50	32	0.065
	P.06	A*02:01	9	33.89	0.50 (SB)	65.24	0.794 (WB)	32.24	1.50 (SB)	1.5	0.632
		A*24:02	9	24272.87	17	25514.28	16.252	N/A	N/A	32	0.18
		B*35:01	9	20096.23	17	20521.63	12.918	N/A	N/A	32	0.112
		B*39:06	9	N/A	N/A	5443.15	2.213	N/A	N/A	32	0.327
		C*03:03	9	36706.18	23	21540.17	13.53	N/A	N/A	16	0.191
		C*07:02	9	34385.58	23	18089.7	11.972	N/A	N/A	32	0.065
pNCL-03	MDA-MB-231	A*02:01	9	12252.01	18	18209.68	11.633	21040.32	32	9	0.178
TLVLSNLSY		A*02:17	9	19774.85	12	25913.51	8.828	24481.5	32	8	0.179
	HCC70	B*15:01	9	65.7	0.60 (WB)	42.63	0.111 (SB)	80.87	1.00 (WB)	0.80 (<-E)	0.378
		B*15:16	9	N/A	N/A	6714.67	2.224	2751.98	15	4	0.244
	P.06	A*02:01	9	12252.01	18	18209.68	23.793	21040.32	32	9	0.178
		A*24:02	9	37119.94	38	31119.19	24.175	N/A	N/A	8	0.068
		B*35:01	9	921.84	1.60 (WB)	236.52	0.461 (SB)	487.32	3.00 (WB)	1.5	0.315
		B*39:06	9	N/A	N/A	33588	37.033	N/A	N/A	10	0.04

Peptide sequence	Host	HLA	Peptide length	NetMHC		NetMHCpan		NetMHCcons		NetCTLpan	PickPocket
				Affinity (nM)	%Rank	Affinity (nM)	%Rank	Affinity (nM)	%Rank	%Rank	Affinity (nM)
		C*03:03	9	36429.23	23	15059.87	9.03	N/A	N/A	9	0.061
		C*07:02	9	25855.82	13	8946.06	4.553	N/A	N/A	9	0.073
pNCL-04	MDA-MB-231	A*02:01	9	5774.27	11	11147.34	14.589	11479.16	32	7	0.232
YAFIEFASF		A*02:17	9	35560.78	28	9786.84	3.892	16316.49	15	3	0.091
	HCC70	B*15:01	9	20.24	0.17 (SB)	25.05	0.460 (SB)	20.03	0.10 (SB)	0.15 (<-E)	0.546
		B*15:16	9	N/A	N/A	103.55	0.156 (SB)	105.42	1.00 (WB)	0.40 (<-E)	0.458
	P.06	A*02:01	9	5774.27	11	11147.34	15.886	11479.16	32	7	0.232
		A*24:02	9	1367.83	1.40 (WB)	677.82	0.757 (WB)	N/A	N/A	0.80 (<-E)	0.321
		B*35:01	9	14.53	0.09 (SB)	6.38	0.020 (SB)	9.44	0.20 (SB)	0.10 (<-E)	0.549
		B*39:06	9	N/A	N/A	11843.65	5.414	N/A	N/A	3	0.17
		C*03:03	9	4.62	0.03 (SB)	6.98	0.029 (SB)	6.29	0.12 (SB)	0.15 (<-E)	0.397
		C*07:02	9	4322.16	1.40 (WB)	890.35	0.423 (WB)	N/A	N/A	0.01 (<-E)	0.084

Underline: The binding score was obtained from the DTU Health Tech (http://www.cbs.dtu.dk/). N/A: not applicable; NetMHC: Threshold for strong binder (%Rank) 0.5; Threshold for weak binder (%Rank) 2; NetMHCPan: Threshold for strong binder (%Rank) 0.5; Threshold for weak binder (%Rank) 2.0; NetMHCcons: Threshold for strong binder (%Rank) 0.5; Threshold for strong binder IC50 < 500 nM; Threshold for weak binder (%Rank) 2.0; Threshold for weak binder IC50 < 2000 nM; NetCTLpan: Threshold for epitope identification (%Rank) 1.0; PickPocket: Prediction values IC50 < 0.500 nM are considered; SB: strong binding; WB: weak binding. Grey highlight boxes indicate candidate peptides that fulfilled at least three out of five selection criteria.

Supplementary Table S9. Characterization of *in silico* predicted NCL epitopes restricted to HLA of patient No. P.07.

Peptide sequence	Host	HLA	Peptide length	NetMHC		NetMHCpan		NetMHCcons		NetCTLpan	PickPocket
				Affinity (nM)	%Rank	Affinity (nM)	%Rank	Affinity (nM)	%Rank	%Rank	Affinity (nM)
pNCL-01	MDA-MB-231	A*02:01	9	236.91	1.80 (WB)	109.03	0.047 (SB)	264.44	4.00 (WB)	1.5	0.607
KMAPPPKEV		A*02:17	9	489.96	0.70 (WB)	2058.36	0.111 (SB)	1700.36	3	0.80 (<-E)	0.496
	HCC70	B*15:01	9	7850.23	12	5019	1.391 (WB)	8387.64	16	9	0.177
		B*15:16	9	N/A	N/A	10018.8	1.359 (WB)	4454	32	10	0.199
	P.07	A*11:01	9	24221.97	21	26106.57	23.642	N/A	N/A	16	0.105
		A*24:02	9	24141.9	16	22743.47	13.418	N/A	N/A	16	0.155
		B*15:02	9	13725.49	5.5	17878.96	15.402	N/A	N/A	16	0.083
		B*15:32	9	N/A	N/A	14134.51	11.958 (WB)	N/A	N/A	16	0.192
		C*08:01	9	N/A	N/A	15949.93	3.478 (WB)	N/A	N/A	6	0.202
		C*12:03	9	66.27	0.25 (SB)	654.4	1.248 (SB)	238.6	3.00 (WB)	10	0.99
pNCL-02	MDA-MB-231	A*02:01	9	33.89	0.50 (SB)	65.24	0.822 (WB)	32.24	1.50 (SB)	1.5	0.632
VLSNLSYSA		A*02:17	9	14881.86	8.5	4084.42	1.823 (WB)	3954.23	5	3	0.492
	HCC70	B*15:01	9	3113.83	6.5	3921.46	6.396	N/A	N/A	8	0.183
		B*15:16	9	N/A	N/A	23716.53	2.776	21154.46	50	32	0.065
	P.07	A*11:01	9	12281.48	10	17325.74	13.321	N/A	N/A	16	0.221
		A*24:02	9	24272.87	17	25514.28	16.252	N/A	N/A	32	0.18
		B*15:02	9	24401.37	13	7760.12	6.322	N/A	N/A	10	0.132
		B*15:32	9	N/A	N/A	10332.67	8.04	N/A	N/A	9	0.194
		C*08:01	9	N/A	N/A	24707.16	7.986	N/A	N/A	16	0.212
		C*12:03	9	26001.15	18	11477.17	11.845	N/A	N/A	32	0.111
pNCL-03	MDA-MB-231	A*02:01	9	12252.01	18	18209.68	11.633	21040.32	32	9	0.178
TLVLSNLSY		A*02:17	9	19774.85	12	25913.51	8.828	24481.5	32	8	0.179
	HCC70	B*15:01	9	65.7	0.60 (WB)	42.63	0.111 (SB)	80.87	1.00 (WB)	0.80 (<-E)	0.378
		B*15:16	9	N/A	N/A	6714.67	2.224	2751.98	15	4	0.244
	P.07	A*11:01	9	1241.77	3	1510.81	2.866	N/A	N/A	3	0.246
		A*24:02	9	37119.94	38	31119.19	24.175	N/A	N/A	8	0.068
		B*15:02	9	313.79	0.17 (SB)	21.03	0.029 (SB)	65.13	0.17 (SB)	0.30 (<-E)	0.353

Peptide sequence	Host	HLA	Peptide length	NetMHC		NetMHCpan		NetMHCcons		NetCTLpan	PickPocket
				Affinity (nM)	%Rank	Affinity (nM)	%Rank	Affinity (nM)	%Rank	%Rank	Affinity (nM)
		B*15:32	9	N/A	N/A	430.62	0.306 (SB)	N/A	N/A	1	0.386
		C*08:01	9	N/A	N/A	31430.85	14.649	N/A	N/A	8	0.078
		C*12:03	9	21182.17	14	3051.98	3.831	N/A	N/A	10	0.061
pNCL-04	MDA-MB-231	A*02:01	9	5774.27	11	11147.34	14.589	11479.16	32	7	0.232
YAFIEFASF		A*02:17	9	35560.78	28	9786.84	3.892	16316.49	15	3	0.091
	HCC70	B*15:01	9	20.24	0.17 (SB)	25.05	0.460 (SB)	20.03	0.10 (SB)	0.15 (<-E)	0.546
		B*15:16	9	N/A	N/A	103.55	0.156 (SB)	105.42	1.00 (WB)	0.40 (<-E)	0.458
	P.07	A*11:01	9	18826.94	15	20123.87	15.908	N/A	N/A	9	0.247
		A*24:02	9	1367.83	1.40 (WB)	677.82	0.757 (WB)	N/A	N/A	0.80 (<-E)	0.31
		B*15:02	9	565.34	0.30 (SB)	13.96	0.017 (SB)	197.45	0.80 (WB)	0.05 (<-E)	0.469
		B*15:32	9	N/A	N/A	66.59	0.017 (SB)	51.92	0.10 (SB)	0.05 (<-E)	0.546
		C*08:01	9	N/A	N/A	582.09	0.076 (SB)	1914.61	1.50 (WB)	0.20 (<-E)	0.384
		C*12:03	9	12.5	0.03 (SB)	10.44	0.034 (SB)	11.6	0.10 (SB)	0.05 (<-E)	0.329

Underline: The binding score was obtained from the DTU Health Tech (http://www.cbs.dtu.dk/). N/A: not applicable; NetMHC: Threshold for strong binder (%Rank) 0.5; Threshold for weak binder (%Rank) 2; NetMHCPan: Threshold for strong binder (%Rank) 0.5; Threshold for weak binder (%Rank) 2.0; NetMHCcons: Threshold for strong binder (%Rank) 0.5; Threshold for strong binder IC50 < 500 nM; Threshold for weak binder (%Rank) 2.0; Threshold for weak binder IC50 < 2000 nM; NetCTLpan: Threshold for epitope identification (%Rank) 1.0; PickPocket: Prediction values IC50 < 0.500 nM are considered; SB: strong binding; WB: weak binding. Grey highlight boxes indicate candidate peptides that fulfilled at least three out of five selection criteria.

Supplementary Table S10. Characterization of *in silico* predicted NCL epitopes restricted to HLA of patient No. P.08.

Peptide sequence	Host	HLA	Peptide length	NetMHC		NetMHCpan		NetMHCcons		NetCTLpan	PickPocket
				Affinity (nM)	%Rank	Affinity (nM)	%Rank	Affinity (nM)	%Rank	%Rank	Affinity (nM)
pNCL-01 KMAPPPKEV	MDA-MB-231	A*02:01	9	236.91	1.80 (WB)	109.03	0.047 (SB)	264.44	4.00 (WB)	1.5	0.607
		A*02:17	9	489.96	0.70 (WB)	2058.36	0.111 (SB)	1700.36	3	0.80 (<-E)	0.496
	HCC70	B*15:01	9	7850.23	12	5019	1.391 (WB)	8387.64	16	9	0.177
		B*15:16	9	N/A	N/A	10018.8	1.359 (WB)	4454	32	10	0.199
	P.08	A*02:03	9	17.68	0.50 (SB)	19.05	0.470 (SB)	18.97	1.50 (SB)	0.80 (<-E)	0.703
		B*46:01/00	9	10980.94	1.70 (WB)	21473.16	3.872 (WB)	N/A	N/A	9	0.251
		C*01:02	9	N/A	N/A	3810.83	0.993 (SB)	N/A	N/A	5	0.135
pNCL-02 VLSNLSYSA	MDA-MB-231	A*02:01	9	33.89	0.50 (SB)	65.24	0.822 (WB)	32.24	1.50 (SB)	1.5	0.632
		A*02:17	9	14881.86	8.5	4084.42	1.823 (WB)	3954.23	5	3	0.492
	HCC70	B*15:01	9	3113.83	6.5	3921.46	6.396	N/A	N/A	8	0.183
		B*15:16	9	N/A	N/A	23716.53	2.776	21154.46	50	32	0.065
	P.08	A*02:03	9	26.21	0.70 (WB)	19.05	0.470 (SB)	14.48	1.00 (SB)	1	0.608
		B*46:01/00	9	22782.08	9	21473.16	3.872 (WB)	N/A	N/A	16	0.104
		C*01:02	9	N/A	N/A	3810.83	0.993 (SB)	N/A	N/A	32	0.019
pNCL-03 TLVLSNLSY	MDA-MB-231	A*02:01	9	12252.01	18	18209.68	11.633	21040.32	32	9	0.178
		A*02:17	9	19774.85	12	25913.51	8.828	24481.5	32	8	0.179
	HCC70	B*15:01	9	65.7	0.60 (WB)	42.63	0.111 (SB)	80.87	1.00 (WB)	0.80 (<-E)	0.378
		B*15:16	9	N/A	N/A	6714.67	2.224	2751.98	15	4	0.244
	P.08	A*02:03	9	10054.96	21	17257.27	31.533	N/A	N/A	1	0.195
		B*46:01/00	9	17538.3	4.5	8822.92	0.735 (SB)	N/A	N/A	16	0.213
		C*01:02	9	N/A	N/A	30132.46	17.613	N/A	N/A	32	0.029
pNCL-04 YAFIEFASF	MDA-MB-231	A*02:01	9	5774.27	11	11147.34	14.589	11479.16	32	7	0.232
		A*02:17	9	35560.78	28	9786.84	3.892	16316.49	15	3	0.091
	HCC70	B*15:01	9	20.24	0.17 (SB)	25.05	0.460 (SB)	20.03	0.10 (SB)	0.15 (<-E)	0.546
		B*15:16	9	N/A	N/A	103.55	0.156 (SB)	105.42	1.00 (WB)	0.40 (<-E)	0.458
	P.08	A*02:03	9	6759.13	16	10183.83	21.532	N/A	N/A	8	0.194
		B*46:01/00	9	107.5	0.01 (SB)	130.58	0.008 (SB)	196.38	0.03 (SB)	0.05 (<-E)	0.52
		C*01:02	9	N/A	N/A	4336.64	1.132 (WB)	2496.64	0.80 (WB)	0.10 (<-E)	0.287

Underline: The binding score was obtained from the DTU Health Tech (http://www.cbs.dtu.dk/). N/A: not applicable; NetMHC: Threshold for strong binder (%Rank) 0.5; Threshold for weak binder (%Rank) 2; NetMHCPan: Threshold for strong binder (%Rank) 0.5; Threshold for weak binder (%Rank) 2.0; NetMHCcons: Threshold for strong binder (%Rank) 0.5; Threshold for strong binder IC50 < 500 nM; Threshold for weak binder (%Rank) 2.0; Threshold for weak binder IC50 < 2000 nM; NetCTLpan: Threshold for epitope identification (%Rank) 1.0; PickPocket: Prediction values IC50 < 0.500 nM are considered; SB: strong binding; WB: weak binding. Grey highlight boxes indicate candidate peptides that fulfilled at least three out of five selection criteria.

Supplementary Table S11. Characterization of *in silico* predicted NCL epitopes restricted to HLA of patient No. P.09.

Peptide sequence	Host	HLA	Peptide length	NetMHC		NetMHCpan		NetMHCcons		NetCTLpan	PickPocket
				Affinity (nM)	%Rank	Affinity (nM)	%Rank	Affinity (nM)	%Rank	%Rank	Affinity (nM)
pNCL-01	MDA-MB-231	A*02:01	9	236.91	1.80 (WB)	109.03	0.047 (SB)	264.44	4.00 (WB)	1.5	0.607
KMAPPPKEV		A*02:17	9	489.96	0.70 (WB)	2058.36	0.111 (SB)	1700.36	3	0.80 (<-E)	0.496
	HCC70	B*15:01	9	7850.23	12	5019	1.391 (WB)	8387.64	16	9	0.177
		B*15:16	9	N/A	N/A	10018.8	1.359 (WB)	4454	32	10	0.199
	P.09	A*02:07	9	27895	5	10197.06	1.223 (SB)	N/A	N/A	2	0.607
		A*24:02	9	24141.9	16	22743.47	13.418	N/A	N/A	16	0.155
		B*46:01	9	10980.94	1.70 (WB)	21473.16	3.872 (WB)	N/A	N/A	9	0.251
		C*01:02	9	N/A	N/A	3810.83	0.993 (SB)	N/A	N/A	5	0.135
pNCL-02	MDA-MB-231	A*02:01	9	33.89	0.50 (SB)	65.24	0.822 (WB)	32.24	1.50 (SB)	1.5	0.632
VLSNLSYSA		A*02:17	9	14881.86	8.5	4084.42	1.823 (WB)	3954.23	5	3	0.492
	HCC70	B*15:01	9	3113.83	6.5	3921.46	6.396	N/A	N/A	8	0.183
		B*15:16	9	N/A	N/A	23716.53	2.776	21154.46	50	32	0.065
	P.09	A*02:07	9	34596.04	11	15466.93	2.323	5806.35	2.00 (WB)	2	0.632
		A*24:02	9	24272.87	17	25514.28	16.252	N/A	N/A	32	0.18
		B*46:01	9	22787.08	9	31136.69	10.734	N/A	N/A	16	0.104
		C*01:02	9	N/A	N/A	20971.68	8.575	N/A	N/A	32	0.019
pNCL-03	MDA-MB-231	A*02:01	9	12252.01	18	18209.68	11.633	21040.32	32	9	0.178
TLVLSNLSY		A*02:17	9	19774.85	12	25913.51	8.828	24481.5	32	8	0.179
	HCC70	B*15:01	9	65.7	0.60 (WB)	42.63	0.111 (SB)	80.87	1.00 (WB)	0.80 (<-E)	0.378
		B*15:16	9	N/A	N/A	6714.67	2.224	2751.98	15	4	0.244
	P.09	A*02:07	9	34596.04	11	15466.93	2.323	N/A	N/A	4	0.178
		A*24:02	9	24272.87	17	25514.28	16.252	N/A	N/A	8	0.068
		B*46:01	9	22787.08	9	31136.69	10.734	N/A	N/A	1.5	0.213
		C*01:02	9	N/A	N/A	20971.68	8.575	N/A	N/A	0.80 (<-E)	0.029
pNCL-04	MDA-MB-231	A*02:01	9	5774.27	11	11147.34	14.589	11479.16	32	7	0.232
YAFIEFASF		A*02:17	9	35560.78	28	9786.84	3.892	16316.49	15	3	0.091
	HCC70	B*15:01	9	20.24	0.17 (SB)	25.05	0.460 (SB)	20.03	0.10 (SB)	0.15 (<-E)	0.546

Peptide sequence	Host	HLA	Peptide length	NetMHC		NetMHCpan		NetMHCcons		NetCTLpan	PickPocket
				Affinity (nM)	%Rank	Affinity (nM)	%Rank	Affinity (nM)	%Rank	%Rank	Affinity (nM)
		B*15:16	9	N/A	N/A	103.55	0.156 (SB)	105.42	1.00 (WB)	0.40 (<-E)	0.458
	P.09	A*02:07	9	39002.38	21	24071.74	5.513	N/A	N/A	3	0.232
		A*24:02	9	1367.83	1.40 (WB)	677.82	0.757 (WB)	N/A	N/A	0.80 (<-E)	0.321
		B*46:01	9	107.5	0.01 (SB)	130.58	0.008 (SB)	196.38	0.03 (SB)	0.05 (<-E)	0.52
		C*01:02	9	N/A	N/A	4336.64	1.132 (WB)	2496.64	0.80 (WB)	0.10 (<-E)	0.287

Underline: The binding score was obtained from the DTU Health Tech (http://www.cbs.dtu.dk/). N/A: not applicable; NetMHC: Threshold for strong binder (%Rank) 0.5; Threshold for weak binder (%Rank) 2; NetMHCPan: Threshold for strong binder (%Rank) 0.5; Threshold for weak binder (%Rank) 2.0; NetMHCcons: Threshold for strong binder (%Rank) 0.5; Threshold for strong binder IC50 < 500 nM; Threshold for weak binder (%Rank) 2.0; Threshold for weak binder IC50 < 2000 nM; NetCTLpan: Threshold for epitope identification (%Rank) 1.0; PickPocket: Prediction values IC50 < 0.500 nM are considered; SB: strong binding; WB: weak binding. Grey highlight boxes indicate candidate peptides that fulfilled at least three out of five selection criteria.

Supplementary Table S12. Characterization of *in silico* predicted NCL epitopes restricted to HLA of patient No. P.10.

Peptide sequence	Host	HLA	Peptide length	NetMHC		NetMHCpan		NetMHCcons		NetCTLpan	PickPocket
				Affinity (nM)	%Rank	Affinity (nM)	%Rank	Affinity (nM)	%Rank	%Rank	Affinity (nM)
pNCL-01	MDA-MB-231	A*02:01	9	236.91	1.80 (WB)	109.03	0.047 (SB)	264.44	4.00 (WB)	1.5	0.607
KMAPPPKEV		A*02:17	9	489.96	0.70 (WB)	2058.36	0.111 (SB)	1700.36	3	0.80 (<-E)	0.496
	HCC70	B*15:01	9	7850.23	12	5019	1.391 (WB)	8387.64	16	9	0.177
		B*15:16	9	N/A	N/A	10018.8	1.359 (WB)	4454	32	10	0.199
	P.10	A*11:01	9	24221.97	21	26106.57	23.642	N/A	N/A	16	0.15
		A*11:02	9	N/A	N/A	26106.57	23.642	N/A	N/A	16	0.15
		B*13:01	9	N/A	N/A	4029.38	3.479 (SB)	N/A	N/A	16	0.173
		B*27:10	9	N/A	N/A	28393.78	17.29	N/A	N/A	16	0.199
		C*04:06	9	N/A	N/A	3164.84	0.702 (SB)	N/A	N/A	6	0.2
		C*12:02	9	N/A	N/A	3833.82	3.332 (WB)	N/A	N/A	10	0.169
pNCL-02	MDA-MB-231	A*02:01	9	33.89	0.50 (SB)	65.24	0.822 (WB)	32.24	1.50 (SB)	1.5	0.632
VLSNLSYSA		A*02:17	9	14881.86	8.5	4084.42	1.823 (WB)	3954.23	5	3	0.492
	HCC70	B*15:01	9	3113.83	6.5	3921.46	6.396	N/A	N/A	8	0.183
		B*15:16	9	N/A	N/A	23716.53	2.776	21154.46	50	32	0.065
	P.10	A*11:01	9	12281.48	10	17325.74	13.321	N/A	N/A	16	0.221
		A*11:02	9	N/A	N/A	17325.74	13.321	N/A	N/A	16	0.221
		B*13:01	9	N/A	N/A	5793.42	5.292	N/A	N/A	32	0.145
		B*27:10	9	N/A	N/A	29439.29	19.279	N/A	N/A	32	0.08
		C*04:06	9	N/A	N/A	22162.88	11.183	N/A	N/A	32	0.127
		C*12:02	9	N/A	N/A	12949.47	11.45	N/A	N/A	16	0.081
pNCL-03	MDA-MB-231	A*02:01	9	12252.01	18	18209.68	11.633	21040.32	32	9	0.178
TLVLSNLSY		A*02:17	9	19774.85	12	25913.51	8.828	24481.5	32	8	0.179
	HCC70	B*15:01	9	65.7	0.60 (WB)	42.63	0.111 (SB)	80.87	1.00 (WB)	0.80 (<-E)	0.378
		B*15:16	9	N/A	N/A	6714.67	2.224	2751.98	15	4	0.244
	P.10	A*11:01	9	1241.77	3	1510.81	2.866	N/A	N/A	3	0.246
		A*11:02	9	N/A	N/A	1510.81	2.866	N/A	N/A	3	0.246
		B*13:01	9	N/A	N/A	10794.81	10.888	N/A	N/A	4	0.196

		B*27:10	9	N/A	N/A	30553.02	21.71	N/A	N/A	3	0.147
		C*04:06	9	N/A	N/A	25038.7	14.23	N/A	N/A	9	0.057
		C*12:02	9	N/A	N/A	3101.05	2.742 (WB)	N/A	N/A	4	0.204
pNCL-04	MDA-MB-231	A*02:01	9	5774.27	11	11147.34	14.589	11479.16	32	7	0.232
YAFIEFASF		A*02:17	9	35560.78	28	9786.84	3.892	16316.49	15	3	0.091
	HCC70	B*15:01	9	20.24	0.17 (SB)	25.05	0.460 (SB)	20.03	0.10 (SB)	0.15 (<-E)	0.546
		B*15:16	9	N/A	N/A	103.55	0.156 (SB)	105.42	1.00 (WB)	0.40 (<-E)	0.458
	P.10	A*11:01	9	18826.94	15	20123.87	15.908	N/A	N/A	9	0.247
		A*11:02	9	N/A	N/A	20123.87	15.908	N/A	N/A	9	0.247
		B*13:01	9	N/A	N/A	1442.33	1.090 (WB)	1509.57	1.50 (WB)	0.40 (<-E)	0.334
		B*27:10	9	N/A	N/A	20604.62	7.967	N/A	N/A	3	0.201
		C*04:06	9	N/A	N/A	1072.27	0.185 (SB)	N/A	N/A	0.20 (<-E)	0.255
		C*12:02	9	N/A	N/A	11	0.009 (SB)	36.94	0.25 (SB)	0.01 (<-E)	0.478

Underline: The binding score was obtained from the DTU Health Tech (http://www.cbs.dtu.dk/). N/A: not applicable; NetMHC: Threshold for strong binder (%Rank) 0.5; Threshold for weak binder (%Rank) 2; NetMHCPan: Threshold for strong binder (%Rank) 0.5; Threshold for weak binder (%Rank) 2.0; NetMHCcons: Threshold for strong binder (%Rank) 0.5; Threshold for strong binder IC50 < 500 nM; Threshold for weak binder (%Rank) 2.0; Threshold for weak binder IC50 < 2000 nM; NetCTLpan: Threshold for epitope identification (%Rank) 1.0; PickPocket: Prediction values IC50 < 0.500 nM are considered; SB: strong binding; WB: weak binding. Grey highlight boxes indicate candidate peptides that fulfilled at least three out of five selection criteria.

Supplementary Table S13. Characterization of *in silico* and MD prediction of NCL epitopes restricted to patients' HLA.

Patients	Protein status		HLA	Algorithms						MD						ELISpot assay (number of spot)	
	NCL	PD-L1		peptides	NetMHC	NetMHCpan	NetMHCcons	NetCTLpan	PickPocket	delta G (SIE)	MM/GBSA	delta MM/GBSA	MM/PBSA	delta MM/PBSA	Distance (Å) (P2-P9)[a]	Day 0	Day 21
P.01	8	3	A*03:01	-	-	-	-	-	-	ND	ND	ND	ND	ND	ND	UP: 0	UP: 47
(P.179)	(H)	(H)	A*11:01	-	-	-	-	-	-	ND	ND	ND	ND	ND	ND	pNCL-01: 3	pNCL-01: 112
			B*07:02	-	-	-	-	-	-	ND	ND	ND	ND	ND	ND	pNCL-02: 6	pNCL-02: 207
			B*46:01	pNCL-1	WB	WB	-	-	-	-12.82	-64.1449	-12.8086	-62.5795	-11.2432	18.883	pNCL-03: 10	pNCL-03: 433
				pNCL-4	SB	SB	SB	E	-	-12.47	-61.3335	-3.3747	-51.5548	6.404	20.856	pNCL-04: 3	pNCL-04: 473
			C*01:02	pNCL-4	-	-	WB	E	ü	ND	ND	ND	ND	ND	ND	Pooled: 3	Pooled: 574
			C*07:02	pNCL-4	WB	WB	-	E	ü	ND	ND	ND	ND	ND	ND		
P.02	10.6	6.6	A*11:01	-	-	-	-	-	-	ND	ND	ND	ND	ND	ND	UP: 5	UP: 43
(P.221)	(H)	(H)	A*30:01	-	-	-	-	-	-	ND	ND	ND	ND	ND	ND	pNCL-01: 5	pNCL-01: 156
			B*13:01	pNCL-4	-	WB	WB	E	ü	-9.68	-43.7991	-1.905	-42.9845	-1.0904	18.911	pNCL-02: 6	pNCL-02: 421
			B*13:02	-	-	-	-	-	-	ND	ND	ND	ND	ND	ND	pNCL-03: 9	pNCL-03: 484
			C*03:04	pNCL-4	-	SB	SB	E	ü	ND	ND	ND	ND	ND	ND	pNCL-04: 4	pNCL-04: 494
			C*06:02	pNCL-4	-	WB	-	E	ü	ND	ND	ND	ND	ND	ND	Pooled: 3	Pooled: 592
P.03	3	0	A*24:10	pNCL-4	-	WB	SB	E	ü	-13.37	-56.2396	-9.5703	-60.3737	-13.7033	20.186	UP: 0	UP: 176
(P.240)	(L)	(L)	A*30:01	-	-	-	-	-	-	ND	ND	ND	ND	ND	ND	pNCL-01: 0	pNCL-01: 856
			B*13:02	-	-	-	-	-	-	ND	ND	ND	ND	ND	ND	pNCL-02: 0	pNCL-02: 386
			B*18:02	pNCL-4	-	WB	SB	E	ü	ND	ND	ND	ND	ND	ND	pNCL-03: 0	pNCL-03: 713
			C*06:02	pNCL-4	-	WB	-	E	ü	ND	ND	ND	ND	ND	ND	pNCL-04: 0	pNCL-04: 750
			C*07:04	pNCL-4	-	WB	-	E	ü	ND	ND	ND	ND	ND	ND	Pooled: 0	Pooled: 646
P.04	2.3	10.6	A*02:03	pNCL-1	SB	SB	SB	E	-	-10.08	-44.3967	6.957	-23.9526	27.4011	20.272	UP: 2	UP: 144
(P.229)	(L)	(H)	A*02:03	pNCL-1	WB	WB	SB	-	-	ND	ND	ND	ND	ND	ND	pNCL-01: 10	pNCL-01: 730
			B*38:02	-	-	-	-	-	-	ND	ND	ND	ND	ND	ND	pNCL-02: 6	pNCL-02: 403

Patients	Protein status		HLA	Algorithms						MD						ELISpot assay (number of spot)	
	NCL	PD-L1		peptides	NetMHC	NetMHCpan	NetMHCcons	NetCTLpan	PickPocket	delta G (SIE)	MM/GBSA	delta MM/GBSA	MM/PBSA	delta MM/PBSA	Distance (Å) (P2-P9)[a]	Day 0	Day 21
			C*07:02	pNCL-4	WB	WB	-	E	ü	ND	ND	ND	ND	ND	ND	pNCL-03: 8	pNCL-03: 534
																pNCL-04: 4	pNCL-04: 772
																Pooled: 6	Pooled: 399
P.05	6.3	3.3	A*02:11	pNCL-1	WB	SB	SB	E	-	ND	ND	ND	ND	ND	ND	UP: 3	UP: 28
(P.213)	(H)	(H)		pNCL-2	WB	SB	SB	-	-	ND	ND	ND	ND	ND	ND	pNCL-01: 7	pNCL-01: 260
			A*24:02	pNCL-4	WB	WB	-	E	ü	ND	ND	ND	ND	ND	ND	pNCL-02: 10	pNCL-02: 111
			B*40:06	-	-	-	-	-	-	ND	ND	ND	ND	ND	ND	pNCL-03: 9	pNCL-03: 266
			B*51:01	pNCL-4	WB	SB	WB	E	ü	-14.85	-68.5461	-17.8151	-62.2022	-11.4712	20.133	pNCL-04: 9	pNCL-04: 368
			C*04:01	-	-	-	-	-	-	ND	ND	ND	ND	ND	ND	Pooled: 12	Pooled: 323
			C*15:02	pNCL-4	-	WB	-	E	ü	ND	ND	ND	ND	ND	ND		
P.06	12	4.6	A*02:01	pNCL-1	SB	SB	WB	-	-	ND	ND	ND	ND	ND	ND	UP: 5	UP: 20
(P.140)	(H)	(H)		pNCL-2	SB	WB	SB	-	-	-12.77	-61.8081	-10.6984	-54.6395	-3.5297	21.45	pNCL-01: 15	pNCL-01: 160
			A*24:02	pNCL-4	SB	WB	-	-	ü	ND	ND	ND	ND	ND	ND	pNCL-02: 22	pNCL-02: 533
			B*35:01	pNCL-3	WB	SB	WB	-	ü	ND	ND	ND	ND	ND	ND	pNCL-03: 15	pNCL-03: 323
				pNCL-4	SB	SB	SB	E	-	ND	ND	ND	ND	ND	ND	pNCL-04: 17	pNCL-04: 311
			B*39:06	-	-	-	-	-	-	ND	ND	ND	ND	ND	ND	Pooled: 9	Pooled: 111
			C*03:03	pNCL-1	WB	WB	-	-	ü	ND	ND	ND	ND	ND	ND		
				pNCL-4	SB	SB	SB	E	ü	ND	ND	ND	ND	ND	ND		
			C*07:02	pNCL-4	WB	WB	-	E	ü	ND	ND	ND	ND	ND	ND		
P.07	12	2.3	A*11:01	-	-	-	-	-	-	ND	ND	ND	ND	ND	ND	UP: 3	UP: 30
(P.141)	(H)	(L)	A*24:02	pNCL-4	WB	WB	-	E	ü	ND	ND	ND	ND	ND	ND	pNCL-01: 5	pNCL-01: 144
			B*15:02	pNCL-3	SB	SB	SB	E	ü	-13.91	-79.7834	-19.6986	-57.612	2.4725	18.14	pNCL-02: 5	pNCL-02: 158
				pNCL-4	-	SB	WB	E	ü	-15.77	-78.8475	-20.4822	-62.4038	-4.0386	17.793	pNCL-03: 9	pNCL-03: 432
			B*15:32	pNCL-4	-	SB	SB	E	-	ND	ND	ND	ND	ND	ND	pNCL-04: 12	pNCL-04: 565

Patients	Protein status		HLA	Algorithms						MD						ELISpot assay (number of spot)	
	NCL	PD-L1		peptides	NetMHC	NetMHCpan	NetMHCcons	NetCTLpan	PickPocket	delta G (SIE)	MM/GBSA	delta MM/GBSA	MM/PBSA	delta MM/PBSA	Distance (Å) (P2-P9)[a]	Day 0	Day 21
			C*08:01	pNCL-4	-	SB	WB	E	ü	ND	ND	ND	ND	ND	ND	Pooled: 5	Pooled: 428
			C*12:03	pNCL-1	SB	SB	WB	-	ü	ND	ND	ND	ND	ND	ND		
				pNCL-4	SB	SB	SB	E	ü	ND	ND	ND	ND	ND	ND		
P.08	7	2.6	A*02:03	pNCL-1	SB	SB	SB	E	-	-10.08	-44.3967	6.957	-23.9526	27.4011	20.272	UP: 3	UP: 25
(P.146)	(H)	(L)		pNCL-2	WB	SB	SB	-	-	ND	ND	ND	ND	ND	ND	pNCL-01: 8	pNCL-01: 358
			B*46:01	pNCL-4	SB	SB	SB	E	ü	-12.47	-61.3335	-3.3747	-51.5548	6.404	20.856	pNCL-02: 14	pNCL-02: 441
			C*01:02	pNCL-4	-	WB	WB	E	ü	ND	ND	ND	ND	ND	ND	pNCL-03: 3	pNCL-03: 314
																pNCL-04: 5	pNCL-04: 232
																Pooled: 6	Pooled: 279
P.09	8	5.3	A*02:07	-	-	-	-	-	-	ND	ND	ND	ND	ND	ND	UP: 0	UP: 162
(P.188)	(H)	(H)	A*24:02	pNCL-4	WB	WB	-	E	ü	ND	ND	ND	ND	ND	ND	pNCL-01: 3	pNCL-01: 294
			B*46:01	pNCL-1	WB	WB	-	-	ü	ND	ND	ND	ND	ND	ND	pNCL-02: 5	pNCL-02: 269
				pNCL-4	SB	SB	SB	E	-	-12.47	-61.3335	-3.3747	-51.5548	6.404	20.856	pNCL-03: 3	pNCL-03: 224
			C*01:02	pNCL-4	-	WB	WB	E	ü	ND	ND	ND	ND	ND	ND	pNCL-04: 1	pNCL-04: 389
																Pooled: 2	Pooled: 397
P.10	2	5	A*11:01	-	-	-	-	-	-	ND	ND	ND	ND	ND	ND	UP: 5	UP: 107
(P.168)	(L)	(H)	A*11:02	-	-	-	-	-	-	ND	ND	ND	ND	ND	ND	pNCL-01: 12	pNCL-01: 510
			B*13:01	pNCL-4	-	WB	WB	E	ü	ND	ND	ND	ND	ND	ND	pNCL-02: 8	pNCL-02: 547
			B*27:10	pNCL-4	-	-	-	-	ü	ND	ND	ND	ND	ND	ND	pNCL-03: 11	pNCL-03: 384
			C*04:06	pNCL-4	-	SB	-	E	ü	ND	ND	ND	ND	ND	ND	pNCL-04: 6	pNCL-04: 719
			C*12:02	pNCL-4	-	SB	SB	E	ü	-12.36	-59.9678	-11.5046	-43.62	4.843	18.784	Pooled: 9	Pooled: 372

ND: not done. -: not significance. UP: unpulsed. Underline: The binding score was obtained from the DTU Health Tech (http://www.cbs.dtu.dk/). N/A: not applicable; NetMHC: Threshold for strong binder (%Rank) 0.5; Threshold for weak binder (%Rank) 2; NetMHCPan: Threshold for

strong binder (%Rank) 0.5; Threshold for weak binder (%Rank) 2.0; NetMHCcons: Threshold for strong binder (%Rank) 0.5; Threshold for strong binder IC50 < 500 nM; Threshold for weak binder (%Rank) 2.0; Threshold for weak binder IC50 < 2000 nM; NetCTLpan: Threshold for epitope identification (%Rank) 1.0; PickPocket: Prediction values IC50 < 0.500 nM are considered; SB: strong binding; WB: weak binding. Grey highlight boxes indicate candidate peptides that fulfilled at least three out of five selection criteria. The distance between P2 and P9 and the orientation of the side chain at P9 was determined by the Simulation Method as described in Fig. 1B and 1F. Arrows indicate the orientation of P9 residues with the respect to the floor of the binding groove of HLA-A*02:01. delta G: the binding free energy; MM/GBSA: molecular mechanics with Generalized Born surface area and MM/PBSA: molecular mechanics with Poisson-Boltzmann surface area. ΔG: the binding free energy; SIE: solvated interaction energy, MM/GBSA: molecular mechanics with Generalized Born surface area and MM/PBSA: molecular mechanics with Poisson–Boltzmann surface area.

Supplementary Table S14. Clinical data of breast cancer patients.

TNBC patients	Conditioned	P.01	P.02	P.03	P.04	P.05	P.06	P.07	P.08	P.09	P.10
**Total NCL IHC score**	1s	8	8	8	3	6	12	8	6	12	3
	2nd	8	12	9	3	4	12	8	6	12	3
	3rd	8	8	8	3	6	12	8	8	12	6
	Average	8.0	9.3	8.3	3.0	5.3	12.0	8.0	6.7	12.0	4.0
**NCL level***	Low (L) or High(H)	H	H	H	L	L	H	H	H	H	L
**Total PD-L1 IHC score**	1st	8	4	4	3	1	8	8	1	9	6
	2nd	8	4	4	3	2	8	8	0	6	12
	3rd	8	4	8	2	2	8	8	1	6	9
	Average	8	4	5.3	2.7	1.7	8	8	0.7	7	9
**PD-L1 level**	Low (L) or High (H)	H	H	H	L	L	H	H	L	H	H
**Neoadjuvant chemotherapy**	No (N) or Yes (Y)	N	N	N	N	N	N	N	N	N	N
**Adjuvant therapy**	C (chemotherapy)<br>R (radiation)<br>H (Hormonal)	C+R	C	C+R	C+H	C+R	C	C+R	C	C	C+R
**Adjuvant chemotherapy (C)**		DOX	DOX	DOX	DOX	DOX	DOX	DOX	DOX	DTX	DOX
**IFN-gamma level (Number of ELISpot at day 0)**	UP	0	5	0	2	3	5	3	3	0	5
	pNCL-01	3	5	0	10	7	15	5	8	3	12
	pNCL-02	6	6	0	6	10	22	5	14	5	8
	pNCL-03	10	9	0	8	9	15	9	3	3	11
	pNCL-04	3	4	0	4	12	17	12	5	12	6
	pNCL-pooled	3	3	0	6	12	9	5	6	2	9
**IFN-gamma level (Number of ELISpot at day 21)**	UP	47	43	176	144	28	20	30	25	162	107
	pNCL-01	112	156	856	730	260	160	144	358	294	510
	pNCL-02	207	421	386	403	111	533	158	441	269	547
	pNCL-03	433	484	713	534	266	323	432	314	224	384
	pNCL-04	473	494	750	772	368	311	565	232	398	719
	pNCL-pooled	574	592	646	399	323	111	428	279	397	372
**OS* (y)**		9.1	6.5	11.4	15.7	6.7	9.3	9.3	9.1	10.2	8.3
**DFS (y)**		11.1	8.5	13.3	17.5	8.6	11.1	11.0	10.6	11.7	9.7

TNBC patients	Conditioned	P.01	P.02	P.03	P.04	P.05	P.06	P.07	P.08	P.09	P.10
**OS** (y)**		13.4	10.5	15.4	19.6	10.6	13.1	13.0	12.7	13.7	11.7
**Diagnostic age (y)**		58.4	72.5	27.9	46.0	32.7	52.3	55.5	59.6	51.3	63.0
**T size (cm)**		3.5	2.0	2.5	2.0	10.0	3.5	3.5	1.5	3.2	2.0
**LN metastasis**	No (N) or Yes (Y)	Y	N	N	N	Y	N	Y	N	N	Y
**Total nodal exam**		16	2	6	33	46	4	20	2	19	24
**No of positive node**		3	0	0	0	36	0	3	0	0	6
**Metastasis**	No (N) or Yes (Y)	Y	Y	Y	N	Y	Y	Y	Y	Y	Y
**Staging**		IV	IV	IV	IA	IV	IV	IV	IV	IV	IV
**Tumor type**		IDC	IDC	IDC	IDC	IDC	IDC	IDC	IDC	IDC	IDC
**Histological grade**		PD	PD	PD	MD	PD	PD	PD	PD	PD	PD
**Vascular invasion**		NA	No	No	No	Yes	No	Yes	No	No	Yes
**Lymphatic invasion**		NA	No	No	No	Yes	No	Yes	No	No	Yes
**Angiolymphatic metastasis**		NA	No	No	No	Yes	No	Yes	No	No	Yes
**Perinodal involved**		Yes	No	No	No	Yes	No	Yes	No	No	No

*Cut-off: NCL low<6 / NCL high =>6. *Cut-off: PD-L1 low<4 / NCL high =>4. H: High. L: Low. UP: Unpulsed. OS*: Surgical date to blood collection (y). OS: Overall survival. OS**: was updated on May 1, 2026. DFS: Disease-free survival. Docetaxel: DTX. Doxorubicin: DOX. LN: Lymph node. IDC: Invasive ductal carcinoma. PD: Poorly differentiated. MD: Moderately differentiated. WD: Well differentiated.
